# XXXIV National Congress and XXVII International Congress of the Spanish Implant Society, Vigo, Spain. September 26 - 28, 2024

**DOI:** 10.4317/medoral.1122335667802

**Published:** 2024-09-03

XXXIV National Congress and XXVII International Congress of the Spanish Implant Society, Vigo, Spain. September 26 - 28, 2024 Abstracts of the scientific communications follow.

