# Supplementary material for: XXXIV National Congress and XXVII International Congress of the Spanish Implant Society, Vigo, Spain. September 26 - 28, 2024
Source: Med Oral Patol Oral Cir Bucal. 2024 Sep 3;29(Suppl 2):S1–S169. doi: 10.4317/medoral.1122335667802 (PMC11393832; doi:10.4317/medoral.1122335667802)
Supplement: Related texts

# Abstract: Narrow implants as an alternative for the rehabilitation of alveolar ridges with horizontal bone deficiency in elderly patients

**Authors:** M.ªL. Castro Rodríguez, B. Rivas Mundiña, M. Pérez-Sayans García, A. García García, A. Blanco Carrión, E.M. Otero Rey

**Affiliations:** *Máster de Medicina Oral, Cirugía Oral e Implantología de la Universidad de Santiago de Compostela*

## Objectives

 To evaluate the efficacy of narrow-diameter implants for the rehabilitation of dental arches with horizontal bone deficiency in elderly patients using implant-supported removable prostheses.

## Material and Methods

 We conducted a retrospective review of the clinical records of 10 patients (6 women and 4 men), with a mean age of 70.1 years, treated by the same clinician between February 2021 and July 2024 in our master's program for narrow implant placement. Various parameters were analyzed, including the O'Leary Plaque Index, implant position within the arch, Oral Health Impact Profile (OHIP), and treatment-related complications.

## Results

 A total of 53 Straumann® Mini Implants Tissue Level, with a diameter of 2.4 mm and lengths of 10 mm and 12 mm, were placed 33 in the mandible and 20 in the maxilla. In the maxillary arch, 25% were placed in the premolar region and 75% in the incisor and canine regions, whereas in the mandibular arch, only 9.9% were placed in the premolar region, with the remaining implants positioned in the incisor and canine regions. Among the 10 participants, only 3 presented with an opposing arch. The O'Leary Plaque Index for these patients averaged 8.02%. Regarding OHIP scores, a comparison between pre-surgical and post-rehabilitation values indicated a significant improvement in four out of five patients, demonstrating enhanced quality of life. There was only one complication, observed in a polymedicated patient with systemic diseases.

## Conclusions

 Narrow-diameter implants present a viable solution for patients with compromised alveolar bone levels. The straightforward surgical protocol is minimally invasive, and the treatment markedly enhances the quality of life for these patients.

# Abstract: Evaluating the effect of musical flow during immediate implant placement: a prospective randomized controlled clinical trial

**Authors:** E. Pellicer, A. Conde Villa, E. Casañas Gil

**Affiliations:** *Universidad Europea de Madrid*

## Objectives

 The aim of this research was to test how musical flow using baroque (BM) and classical era (CM) music as a non-pharmacological therapy can reduce anxiety and pain levels in patients undergoing IIP (Immediate Implant Placement).

## Material and Methods

 In this randomized clinical trial, seventy-eight patients in need of IIP were included. Each patient was assigned to one of the three experimental groups with simple randomization: Group I (n=26) listened to BM; Group II (n=27) listened to CM; and Group III (n=25) did not listen to music and was the control group (C). The dependent physiological variables analyzed were systolic blood pressure (SBP), diastolic blood pressure (DBP), heart rate (HR) and oxygen saturation (SpO2). The psychological dependent variable analyzed was the Modified Dental Anxiety Scale (MDAS) and the Visual Analog Scale (VAS), measured before and after the intervention. In all cases, the level of statistical significance was set at p<0.01.

## Results

 Statistically significant differences were found in the SBP decrease in the CM group (p=0.001, CI=1.9716–6.5840) and the BM group (p=0.003, CI=1.4450–6.4396). Anxiety levels during the intervention decreased in both groups that listened to music: BM group (p=0.002, CI=0.645–2.662) and CM group (p=0.000, CI=1.523–3.884).

## Conclusions

 Patients undergoing IIP surgery may record lower levels of SBP when listening to BM and CM than patients who were not exposed to the musical flow, improving their anxiety levels.

# Abstract: Narrow implants for the rehabilitation of alveolar ridges with horizontal bone compromise. Descriptive study

**Authors:** N. Matala Álvarez, L. Álvarez Iglesias, J.M. Somoza Martí, P. Gándara Vila, A. Blanco Carrión, E. Otero Rey

**Affiliations:** *Master of Oral Medicine, Oral Surgery and Implantology, University of Santiago de Compostela*

## Objectives

 Our main objective has been to analyze whether the placement of narrow implants in patients with horizontally compromised bone crests is a good treatment alternative for implant rehabilitation.

## Material and Methods

 For the design of this study we have reviewed the medical records of 10 patients (6 women and 4 men), with an average age of 70,1 years, all patients have been operated between February 2021 and July 2024 in the Master of Oral Medicine, Oral Surgery and Implantology of the University of Santiago de Compostela, always by the same operator and with the Straumann mini implant ® system and we have analyzed the following variables: type of bone, drilling protocol and the need or not of guided bone regeneration.

## Results

 We placed a total of 53 implants (33 in the mandible and 20 in the maxilla), 33 in women and 20 in men. All patients were revised according to our protocol which will be described in the study.

## Conclusions

 Narrow implant placement represents a good treatment alternative for elderly patients with compromised bone crestt width, due to the simplicity of the surgical protocol and the minimally invasive technique.

# Abstract: Different therapeutic options for the implant rehabilitation of dental arches with horizontal bone involvement in geriatric patients. Bibliographic review

**Authors:** P. Rodrigo Cáceres, L. Campo, J.M. Somoza Martín, P. Gándara Vila, A. Blanco Carrión, E.M. Otero Rey

**Affiliations:** *Universidad Santiago de Compostela*

## Objectives

 The purpose of this bibliographic review is to compare different forms of implant rehabilitation in the maxilla and mandible with horizontal bone involvement in geriatric patients. Different techniques will be addressed: guided bone regeneration, block graft, surgical expansion and placement of mini implants.

## Material and Methods

 ClinicalKey , and Cochrane Library databases were searched for studies addressing rehabilitation with mini-implants , guided bone regeneration, surgical expansion, and block grafting. The PICO question: Is the use of mini implants feasible in patients with marginal bone loss compared to other treatments?

## Results

 Of the studies selected for the review, patients whose age was 65 years or older were evaluated, of which the group that was rehabilitated with mini-implants for the retention of overdentures, resulted in a mini-implant survival rate of 90.58%, being the most frequent failures in the upper jaw. Marginal bone loss was similar to that of conventional implants (<1.5mm) and an increase in satisfaction and quality of life was observed after rehabilitation treatment with mini implants

## Conclusions

 From the literature review we can conclude that mini implants prove to be a valid option for patients who do not want or cannot have conventional implants placed, either due to limited bone availability or to avoid more aggressive surgeries. Mini implants offer survival rates comparable to those of conventional implants, acceptable marginal bone loss, good aesthetic and functional results, good quality of life and less economic investment versus conventional implants, making this a good treatment alternative for our geriatric patients.

# Abstract: Prediction using convolutional neural network (CNN) of the Durability of Dental Implants affected by peri-implantitis

**Authors:** A. Boquete Castro, K.D. Mora Barrios

**Affiliations:** *Private practice*

## Objectives

 Develop, using AI, a system that allows predicting the time it will take for an implant affected by peri-implantitis to be lost, if no therapeutic activity is carried out on it.

## Material and Methods

 Patient data including demographic and clinical information was collected, along with radiographic images of their dental implants. The risk factors considered in this study were: diabetes, smoking, age, gender and location of the implant. A convolutional neural network (CNN) model was used to analyze two-dimensional radiographic images and CBCT slices and carry out a predictive model with two possible outputs: the degree of implant involvement and the estimated time until implant loss in case of do not undergo any treatment. The model was trained and validated using a dataset of images annotated with implant loss information. The risk score calculation was performed as follows: Diabetes: Yes (1 point), No (0 points) Smoker: No (0 points), Yes 0-10 per day (1 point), Yes + 10 per day (2 points) Age: 35-50 (0 points), 51-70 (1 point), +71 (2 points) Gender: Male (1 point), Female (0 points) Maxilla: Upper (1 point), Lower (0 points) The total risk score was used to categorize patients into three risk levels: low, moderate, and high.

## Results

 The developed model provides an approximate prediction of the time to implant loss, adjusted according to the risk score. The results are presented with examples of predictions made for a set of test images, highlighting the correlation between the risk score and the estimated time to implant loss.

## Conclusions

 This study shows an advanced predictive model developed using AI to evaluate the durability of dental implants based on individual risk factors. The use of clinical images and basic patient data allows.

# Abstract: Analysis of the accuracy and veracity of the 2D and 3D spatial position of two types of scannable healing abutments. An *in vitro* study

**Authors:** A.F. Stran Lo Giudice, J. Bartolomé Lechuga, M. Santmartí Oliver, C. Cobo, C. Madrigal Martínez-Pereda, G. Pradíes Ramiro

**Affiliations:** *Máster en Cirugía Bucal e Implantología (Universidad Complutense de Madrid)*

## Objectives

 The aim of this study is to analyze the accuracy and veracity of two types of scannable healing abutments when it comes to spatially reproducing the position of the implants to which they are screwed from the use of an intraoral scanner.

## Material and Methods

 Four implants were placed in an edentulous plastic jawbone and two types of scannable abutments were screwed onto them: group 1 = Encode and group 2 = Iphysio. Next, the typodont was subjected to a CBCT to obtain the "ground truth" of the positions of the implants with the abutments in place and then the typodont was scanned with an intraoral scanner. The resulting files, in DICOM and STL format respectively, were aligned and compared using Geomagic Control X software for analysis in terms of their three-dimensional position (microns) and angulation (degrees) in each group.

## Results

 The Student's T tests and multivariate analysis in both the 3D and 2D comparisons offered statistically significant results, therefore, it is not possible to accept the null hypothesis, and nothing prevents considering the alternative hypothesis as valid.

## Discussion

: The results obtained, even rejecting the null hypothesis, are accepted by the literature when establishing a range in which it is possible to perform fixed restorations on implants from the scannable Encode and Iphysio abutments.

## Conclusions

 Scannable abutments are in favor of the one abutment-one time concept, a more efficient digital workflow, support the interest in research on Encode abutments and present a new line of research related to the study of Iphysio abutments.

# Abstract: Are monobloc zirconia implants a predictable medium-term alternative to titanium implants? A systematic review and meta-analysis

**Authors:** M. Santmartí Oliver, E. Ibáñez Prieto, A. González García, C. Pérez López, E. Cáceres Madroño

**Affiliations:** *Postgrado Especialización en Cirugía Bucal e Implantología de la Universidad Complutense de Madrid*

## Objectives

 The aim of this study is to evaluate the overall success and survival rates, marginal bone loss (MBL), and various biological parameters of one-piece zirconia implants (O-PZI) supporting fixed prostheses after a follow-up period of at least three years.

## Material and Methods

 A systematic review and meta-analysis were conducted following PRISMA guidelines. An electronic search in PubMed, Scopus, The Cochrane Library, and Web of Science identified studies with a minimum of 10 patients and/or 20 implants, and a follow-up of three years or more. Data on implant survival, success rates, MBL, probing depth (PD), bleeding index (BI), plaque index (PI), and patient-reported outcome measures (PROMs) were extracted and analyzed.

## Results

 Fourteen studies met the inclusion criteria, encompassing 1,621 O-PZI placed in 1,588 patients. The survival and success rates at the three-year follow-up were 94.4% (95% CI: 90.4%-98.4%) and 91.6% (95% CI: 84.2%-98.9%), respectively. MBL averaged 0.231 mm (95% CI: 0.190-0.272). PD, BI, and PI data indicated good soft tissue health around the implants. PROMs, though evaluated in only three studies, showed high patient satisfaction with the esthetic and functional outcomes of zirconia implants.

## Conclusions

 One-piece zirconia implants demonstrate promising mid-term clinical outcomes with high survival and success rates, comparable to titanium implants. They also show favorable MBL and soft tissue parameters, suggesting their viability as a predictable alternative for dental rehabilitation. However, further long-term studies with larger sample sizes and randomized clinical trials are necessary to fully establish zirconia implants as a standard option in clinical practice.

# Abstract: Root coverage with tunnel technique or coronally advanced flap? A systematic review with meta-analysis

**Authors:** A. González Olazabal, K. López Ortega, V. Ramil Novo, A. Sande Seijas, F. Seijas Naya, A. Pérez González

**Affiliations:** *Oral Medicine, Oral Surgery and Implantology Unit (MedOralRes), Faculty of Medicine and Dentistry, University of Santiago de Compostela, Santiago de Compostela, Spain*

## Introduction

 Gingival recession, characterized by the apical displacement of the gingival margin, presents challenges to oral health. This study compares the effectiveness of coronally advanced flap (CAF) and tunnel technique (TT) for treating gingival recessions.

## Material and Methods

 A systematic review were performed with 26 studies evaluating root coverage outcomes were included. Fourteen articles were included for the meta-analysis. Three groups were analyzed: Group 1 compared TT with connective tissue graft (CTG) versus CAF with CTG; Group 2 examined TT with CTG and/or other biomaterials versus TT with CTG alone; Group 3 compared TT and CAF, regardless of complementary biomaterials. Meta-analysis assessed mean root coverage (MRC), complete root coverage (CRC), and keratinized tissue gain.

## Results

 In Group 1, TT with CTG demonstrated superior MRC compared to CAF with CTG (-8.68 CI95% -17.19 to -0.17; p=0.0457). In Group 2, TT with CTG and/or other biomaterials showed similar MRC (4.17 CI95% -17.91 to 26.26; p=0.7110) and CRC (0.37 CI95% -1.14 to 1.89; p=0.6269) to TT with CTG alone, with variations in keratinized tissue gain. Group 3 indicated higher potential MRC for TT compared to CAF (5.73 CI95% -8.90 to 13.55; p=0.685) but without statistically significant differences.

## Conclusions

 This study suggests that TT with CTG might offer better root coverage than CAF with CTG. However, biomaterial selection requires consideration. Further research is needed to establish the optimal technique for addressing gingival recessions. The diversity of biomaterials used in conjunction with the main surgical techniques could complicate the interpretation of overall conclusions.

# Abstract: Regeneration of periodontal intrabony defects using platelet‐rich fibrin (PRF): a systematic review and network meta‐analysis

**Authors:** L. Álvarez Iglesias, F. França Vieira e Silva, C. López Rodriguez, C. Menéndez Valdés, M. Somoza Martin, E. Otero Rey

**Affiliations:** *Master de Medicina Oral, Cirugía e Implantología Oral. Universidad de Santiago de Compostela*

## Objectives

 One of the most promising approaches to correct periodontal bone defects and achieve periodontal regeneration is platelet- rich fibrin (PRF). This systematic review and meta-analysis aimed to evaluate the regeneration of periodontal bone defects using PRF compared to other regenerative treatments.

## Material and Methods

 The data search and retrieval process followed the PRISMA guidelines. An electronic search of MEDLINE, Cochrane, and PubMed databases was performed, selecting exclusively randomized clinical trials where the following were measured: probing depth reduction (PD), clinical attachment level gain (CAL), and radiographic bone fill (RBF).

## Results

 Out of 284 selected articles, 32 were chosen based on inclusion criteria. The use of platelet- rich fibrin (PRF) + open flap debridement (OFD), PRF + metformin, PRF + platelet-rich plasma (PRP), and PRF + OFD/bone graft (BG) significantly reduced PD and improved CAL and RBF. However, the combination of PRF + BG, PRF + metformin, and PRF + STATINS reduced CAL.

## Conclusions

 This systematic review and meta-analysis concluded that the intervention of PRF combined with different treatments such as metformin, OFD, PRP, BG, and STATINS has a significant impact on improving PD and CAL. The use of PRF significantly improved the regeneration of periodontal bone defects compared to other treatments.

# Abstract: Results of local application and/or surface coating of dental implants with bisphosphonates

**Authors:** M. Ruiz Rincón, A. Montserrat Bauer, A.F. Stran Lo Giudice, F. Pérez González, S. Saiz, C. Meniz García

**Affiliations:** *Facultad de Odontología, Universidad Complutense de Madrid*

## Objectives

 This systematic review aimed to assess the effectiveness of the local application and surface coating of dental implants with bisphosphonates.

## Material and Methods

 This systematic review was conducted according to PRISMA guidelines and the PICO question to evaluate the impact of bisphosphonates on endosseous dental implants. Electronic searches were conducted in the Medline (PubMed), Web of Science, and Cochrane databases for relevant articles published up to June 2024, using specific MeSH terms. The parameters analyzed were marginal bone level and Implant Stability Quotient (ISQ). Quality assessment was performed using the Risk of Bias 2.0 tool for randomized clinical trials.

## Results

 The initial electronic database search yielded 596 articles, from which a total of 4 studies were selected for data extraction. The 4 studies included a total of 105 patients over 18 years old with maxillomandibular edentulous spaces. When comparing implant rehabilitations where bisphosphonates were used with those where they were not, variations in marginal bone loss were observed, but no significant differences were found in the ISQ. Implants treated with bisphosphonates presented survival rates ranging from 93.75% to 100%.

## Conclusions

 No significant differences were observed between the use and non-use of surface coating and local application of bisphosphonates, although further comparative studies are needed to confirm these findings.

# Abstract: Bone regeneration in critical-sized mandibular symphysis defects using bioceramics with or without bone marrow mesenchymal stem cells in healthy, diabetic, osteoporotic, and diabeticosteoporotic rats

**Authors:** A. Pose Miguel, F. Camacho Alonso, A. Fernández Gato, S. Baña Souto, I. Prado Pena, A. Gamallo Moure

**Affiliations:** *Máster Medicina Oral, Cirugía Oral e Implantología Universidad Santiago de Compostela*

## Objectives

 To compare new bone formation in mandibular critical-sized bone defects (CSBDs) in healthy, diabetic, osteoporotic, and diabetic-osteoporotic rats filled with bioceramics (BCs) with or without bone marrow mesenchymal stem cells (BMSCs).

## Material and Methods

 A total of 64 adult female Sprague-Dawley rats were randomized into four groups (n = 16 per group): Group 1 healthy, Group 2 diabetic, Group 3 osteoporotic, and Group 4 diabetic-osteoporotic rats. Streptozotocin was used to induce type 1 diabetes in Group 2 and 4, while bilateral ovariectomy was used to induce osteoporosis in Group 3 and 4. The central portion of the rat mandibular symphysis was used as a physiological CSBD. In each group, eight defects were filled with BC (hydroxypatatite 60% and β-tricalcium phosphate 40%) alone and eight with BMSCs cultured on BC. The animals were sacrificed at 4 and 8 weeks, and the mandibles were processed for micro-computed tomography to analyze radiological union and bone mineral density (BMD); histological analysis of the bone union; and immunohistochemical analysis, which included immunoreactivity of vascular endothelial growth factor (VEGF) and bone morphogenetic protein 2 (BMP-2).

## Results

 In all groups (healthy, diabetics, osteoporotics, and diabetics-osteoporotics), the CSBDs filled with BC + BMSCs showed greater radiological bone union, BMD, histological bone union, and more VEGF and BMP-2 positivity, in comparison with CSBDs treated with BC alone (at 4 and 8 weeks).

## Conclusions

 Application of BMSCs cultured on BCs improves bone regeneration in CSBDs compared to application of BCs alone in healthy, diabetic, osteoporotic, and diabeticosteoporotic rats.

# Abstract: Influence of diferente types of dental implants surfaces on the tribocorrosion behaviour of CpTi/Tialloys in the oral environment

**Authors:** D. Alfaro Aroca, P.C. Vaz Fernandes, A. Alves, J. Gil, M.M. Gallas Torreira

**Affiliations:** *Department of Surgery and Medical-Surgeries Specialities. Stomatology Area. Universidade de Santiago de Compostela. Laeta/INeGi Research Unit Porto University, FMDUP. Prevage and Catevage CEO. Center of MicroElectroMechanical Systems-University of Minho (Portugal)*

## Introduction

 Materials and surfaces developed for dental implants and dental implantsupported restorations need to understand degradation processes that take place in the oral cavity. The oral environment subjects Titanium dental implants to varying conditions like changes in pH, temperature, and saliva contamination leading to chemical corrosion.

## Objectives

 In this study, the combined effect of chemical corrosion and wear (so-called tribocorrosion) in the degradation of four different dental implant surfaces varying pH oral environment was investigated. Therefore, the goal of the study was to analyze the corrosion and tribocorrosion behavior of differents types of implant surfaces.

## Material and Methods

 Titanium (CpTi) discs were subjected to sliding tests in artificial saliva at varying pHs: 3.0 to 7.0. Electrochemical test, tribocorrosion test and topographhy, microstructure an chemical. Commercially pure titanium (CpTi) and Ti-6Al-4V alloy were used as controls.

## Results

 The analysis of the results indicated that pH has no influence on the behavior of surfaces at corrosion rate (ipass). Corrosion kinetics are therefore not altered. However, looking at the Breakdown Potential (Eb) data, it is observed that for pH7 there are changes at very high potentials, above 1.3 Volts. Comparing the HCl and AC treated surfaces with the HCl_C and AC_C, it is concluded that the Contact Ti surface has benefits in clinical behavior, as does the acid treatment, without the disadvantage of accelerating the corrosion of Titanium, which happens on surfaces with acid treatment only. In other words, on acidic surfaces, this treatment improves osseointegration, but worsens the implant's behavior, because corrosion resistance is lost. With the Contact Ti surface, the corrosion behavior is very close to that of Titanium, significantly improving it, without losing the advantages of surface passivation treatments (hydrochemical, acid treatments).

## Conclusions

 Despite reports of CpTi being electrochemically stable down to pH 2.0, this study suggests degradation peaks at near neutral pH

# Abstract: Clinical performance of subperiosteal implants. Systematic review

**Authors:** A. Fuertes Merino, A. García Marqués, P. Benito López, L.M. Sáez Alcaide, C. Madrigal Martínez-Pereda, C. Meniz García

**Affiliations:** *Facultad de Odontología, Universidad Complutense Madrid*

## Objectives

 The aim of this systematic review is to evaluate another possibility to treat the lack of sufficient alveolar bone with subperiosteal implants.

## Material and Methods

 The PICO model was followed to establish selection criteria, encompassing population, intervention, comparison, and outcomes. Studies involving humans, with a minimum follow-up of 6 months and published between 2013 and 2023, were included. The search strategy was conducted across various databases, followed by a selection and data collection process. Methodological quality was assessed using standard tools.

## Results

 Following a selection process, 20 studies were included. The survival rate of implants ranged between 95.8% and 100%, with an average of 98.7%. Complications included untreatable infections (11.1%) and exposure of the metal frame (5 cases), all of which were treatable.

## Conclusions

 Subperiosteal implants demonstrate acceptable survival rates and low complication rates, although further long-term research is needed. Their role in oral rehabilitation in patients with severe bone loss is promising, but more studies are required to support their clinical use.

# Abstract: Torque and Implant stability quotient (ISQ) analysis of dental implants at the bone and tissue level: a randomized clinical trial

**Authors:** A. Elkattan, M. Blázquez, R. Ayuso Montero, X. Roselló Llabrés, J. López-López

**Affiliations:** *Máster Medicina, Cirugía e Implantología - Universitat de Barcelona – Facultad de Medicina y Ciencias de la Salud*

## Objectives

 To analyse the implant torque and (ISQ) on the day of surgery and to evaluate the ISQ of two types of dental implants at the bone and tissue level after 90 days. That have been placed in the Master of Medicine, Surgery & Oral Implant program at the University of Barcelona.

## Material and Methods

 This RCT aimed to measure implant torque and ISQ for implants placed at BL and TL on the day of surgery, and to assess ISQ after 90 days. The study was conducted at the University of Barcelona Dental Hospital and was approved in the HOUB. The study sample included 81 implants placed in 18 patients. Patients were divided into two groups: the BL group (39 implants) and the TL group (42 implants).

## Results

 The patients who received 81 implants experienced a typical postoperative course, free of complications and with a low rate of inflammation, as expected for this type of procedure. The average insertion torque in the BL group was ranging (40.71-35 NCM), while in the TL group it was ranging (40.46 - 37 NCM). Regarding implant stability, measured through the ISQ, BL group showed means of (69.84 - 65.62) for the H, and means of (73.26 - 64.64) for the V. For the TL group, ISQ means were (68.76 - 65.84) for the H, and (68.73 - 69.46) for the V.

## Conclusions

 The findings for implant torque indicated that the TL had a torque > 45 NCM compared to the BL with > 35 NCM, with an average of 37.85 NCM for BL and 38.73 NCM for TL. On the day of surgery, the ISQ had a mean of 68.59 for BL and 68.20 for TL. After 90 days, the TL showed a higher ISQ with a mean of 65.198, compared to 63.31 for the BL.

# Abstract: Dentin as a Regenerative Material in Dentistry. Updated Literature Review

**Authors:** L. Darwish Mateos, J.M. Acuña Pedrosa, A. Boquete Castro

**Affiliations:** *Master de formación permanente en cirugía bucal, implantología yt periodoncia. Septiembre 2023 - noviembre 2024. Jmap formación acca learning. Sevilla*

## Objectives

 To evaluate the use of autologous dentin as a regenerative material in dentistry, comparing it with other biomaterials in order to analyze its applications in alveolar preservation, guided bone regeneration, and maxillary sinus lift procedures.

## Material and Methods

 A literature review was conducted using the search engines PubMed, Medline, and Google Scholar, limiting the search to the last five years and including important previous references. Articles in English, French, and Spanish were included, covering clinical trials in humans and cell culture studies.

## Results

 Autologous dentin shows high biocompatibility and good osteoconductive properties, being effective in bone regeneration. Dentin grafts achieved 70-75% regeneration at 3 months, 80-85% at 6 months, 90-92% at 12 months, and 95% at 24 months. Dentin proved to be effective in alveolar preservation, guided bone regeneration, and maxillary sinus lift. Clinical studies showed bone regeneration exceeding 85% at 6 months in alveolar preservation, 80% at 6 months in guided bone regeneration, and 88% at 12 months in maxillary sinus lift. New devices for dentin processing have facilitated the use of this material.

## Conclusions

 Dentin is a promising regenerative material in dentistry, offering advantages over traditional materials. Although it has limitations, it shows high biocompatibility, mechanical resistance, and osteoconductive properties. This technique requires further investigation as there are not many long-term studies available.

# Abstract: Guided surgery: in dentistry implants

**Authors:** R.A. Garcia Cuenca

**Affiliations:** *CLINICA IMPLANT GRUP*

## Objectives

 This technique has several objectives in which it seeks to improve the precision, efficiency and results of the treatment. Minimizing the deviation of the location of the implants, thus resulting in better integration and stability. Reducing the time necessary for surgery, allowing smaller incisions and less trauma to the soft tissues, thus reducing post-surgical inflammation and discomfort. It also allows a more harmonious integration of the surgical face with the prosthetic phase. Thus providing a less stressful and more comfortable experience for the patient.

## Results

 Guided surgery is an effective and precise technique, with multiple benefits, improving clinical results and the patient experience. The use of personalized surgical guides allows exact placement of the implants. For this technique, detailed planning is necessary, reducing positioning errors compared to conventional guidance, thus avoiding damage to anatomical structures such as nerves and maxillary sinuses. Improving the integration of the implant with the bone, giving greater stability and long-term durability. he precision of this procedure contributes to a more comfortable experience for the patient, reducing postoperative pain and recovery time, allowing patients to return to their normal activities sooner. According to the Journal of Clinical Periodontology, guided surgery significantly reduces implant position errors compared to manual surgery. Research from the International Journal of Oral and Maxillofacial Implants showed that implants placed with guided surgery have a higher success rate and greater long-term stability

## Conclusions

 Guided surgery in dental implantology, through the use of advanced technology such as CBCT and three-dimensional planning software, a superior standard in surgical precision and predictability of results. This method reduces the incidence of anatomical complications and optimizes the primary stability of the implants, and also improves the effectiveness of the procedure and the patient's experience, establishing itself as an innovative model in dental practice.

# Abstract: Implant placement in patients with antiresorptive drugs: Is it a cause for concern?

**Authors:** M. Ouazzani Touhami, M. Ruiz Rincón, A. Montserrat Bauer, C. Meniz García, I. Leco Berrocal

**Affiliations:** *Postgrado de Especialización en Cirugía Bucal e Implantología, Universidad Complutense de Madrid*

## Objectives

 The objective of this study is to evaluate the incidence of drug-associated osteonecrosis in implant treatment and to evaluate the different treatment alternatives according to the stages of osteonecrosis.

## Material and Methods

 A search was carried out in the PubMed database using the following search strategy: "osteonecrosis of the jaw" AND "dental implant" AND "management". We included studies published in the last 10 years that were accessible and were not animal studies.

## Results

 In total, applying the search strategy and the inclusion and exclusion criteria, 9 articles were obtained. These studies showed that the success rate of implant placement in patients with antiresorptive drugs is high. Although certain associated complications were reported, such as delayed healing, osseointegration and post osseointegration failure. These findings underscore the need for careful evaluation and planning in patients receiving antiresorptive medication and requiring dental implants, to minimize the risks and maximize the benefits of treatment, as the functional and psychosocial benefits of this intervention outweigh the associated risks. Treatment is carried out following the guidelines of the American Association of Oral and Maxillofacial Surgeons. In stages I and II, conservative treatment is performed, while in stage III, surgical debridement and bone resection is performed. There are other treatments that do not present high scientific evidence, such as the use of platelet concentrates, teriparatide, laser therapy, hyperbaric oxygen and ozone applications.

## Conclusions

 In summary, the benefits of implant placement in patients with antiresorptive drugs outweigh the associated risks. Treatments for drug-associated osteonecrosis are varied, with non-surgical, surgical and innovative treatments.

# Abstract: Understanding Solid-Based Platelet-Rich Fibrin Matrices in Oral and Maxillofacial Surgery: An Integrative Review of the Critical Protocol Factors and Their Influence on the Final Product

**Authors:** S. Valencia Martínez, A.O. Salgado Peralvo, M. Gallas Torreira, T. García Carnicero, M.J. Rodríguez Blanco, P. Gándara Vila

**Affiliations:** *Máster de Medicina oral, Cirugía oral e Implantología (USC)*

## Objectives

 Platelet-rich fibrin (PRF) is a second-generation platelet concentrate whose use in clinical practice has been widely disseminated. This has led to the development of several commercial protocols, creating great confusion as to the terminology and implications of each of them. This integrative review aims to identify the critical factors of each of the phases of the solid-based PRF matrix protocol and their possible influence on their macro and microscopic characteristics.

## Material and Methods

 An electronic search of the MEDLINE database (via PubMed), Web of Science, Scopus, LILACS, and OpenGrey was carried out. The search was temporarily restricted from 2001 to 2022. After searching, 43 studies were included that met the established criteria.

## Results

 There were numerous factors to consider in the PRF protocol, such as the material of the blood collection tubes, the duration of phlebotomy, the parameters related to blood centrifugation, the time from centrifugation to dehydration of the fibrin clots and their dehydration into membranes, as well as the time to clinical use.

## Conclusions

 These factors influenced the macro- and microscopic characteristics of the PRF and its physical properties, so knowledge of these factors allows for the production of optimised PRF by combining the protocols and materials.

# Abstract: Clinical results of Monoblock Compressive Implants

**Authors:** P. Benito López, A. Fuertes Merino, A. García Marqués, C. Mallagray Montero, C. Meniz García, C. Madrigal Martínez-Pereda

**Affiliations:** *Universidad Complutense de Madrid*

## Objectives

 this review intends to describe the characteristics and qualities of monoblock compressive basal implants, as well as their advantages, disadvantages, indications, and contraindications based on a literature review, with the objective to present relevant clinical results.

## Material and Methods

 A bibliographic review of the last 10 years was carried out based on the PUBMED and COCHRANE® databases, complemented by clinical cases treated with these implants in different indications and the subsequent creation of immediate loading prostheses.

## Results

 Compressive monoblock implants have advantages such as avoiding bone grafts or sinus lift procedures. Its intraosseous part has a special compression thread that causes the creation of a dense layer of bone around the implant, favouring its primary stability and subsequently its osseointegration. As there is no connection between the body of the implant and its abutment, these implants do not present the problems of possible leaks or micromovements, loosening, and/or breakage of screws.

## Conclusions

 Compressive basal implants constitute an alternative for implant treatment, especially in cases of horizontal bone deficiency. In addition, they allow flapless surgeries and immediate loading protocols to be performed predictably, improving patient satisfaction. However, it is necessary to have more scientific work and clinical studies to corroborate these advantages.

# Abstract: Formwork Technique with Mesh in Elevations of Sinus Floors with Large Perforations of the Schneider Membrane. Pilot Case

**Authors:** E.R. Fernández Castellano

**Affiliations:** *Clinica Dr. Fernández*

## Introduction

 Currently, maxillary sinus floor elevation is one of the most common procedures used in implantology practice. Despite its predictability, the technique is not without complications, such as graft material dispersion in the sinus cavity, wound dehiscence, hematoma, fenestrations, oroantral fistulas, epistaxis, acute sinusitis, and Schneider membrane perforations. The treatment of the latter can be complex, and depending on its extent, surgery deferral may be necessary, leading to increased patient morbidity.

## Case Report

 A patient with apical surgery underwent sinus floor elevation with a significant Schneider membrane perforation using a new approach involving titanium mesh, resorbable membrane, and xenograft. This allowed the continuation of surgery, reducing the number of interventions and patient morbidity.

## Conclusions

 Despite limitations due to a small sample size, this case report demonstrates that addressing large Schneider membrane perforations and placing implants is effective and predictable using the technology and approach of mesh formwork with titanium.

# Abstract: Digitally Guided Immediate Implant Placement: A case Study

**Authors:** D. Güler, A. Serra Ristol, E. Willaert, P. Barenblit

**Affiliations:** *Clinical Master's in Implantology and Prothesis Buccal, Barcelona University*

## Introduction

 Immediate implant placement has become popular for its ability to shorten treatment time and reduce bone resorption post-extraction with the right technique. A significant challenge is positioning the implant prosthetically in the anterior sector due to the curvature of the residual palatal bone. Recent technological advancements, including digital guidance, have improved the precision and efficiency of this procedure.

## Case Report

 A 75-year-old male presented with a broken tooth-supported bridge spanning positions 22-24. Tooth 22 was deemed unrestorable, and the patient already had an implant in position 25. Radiographic examination revealed sufficient residual bone and a substantial cortical bone in the vestibule, making the patient a candidate for immediate implant placement in position 22. To enhance precision, we opted to use a digitally guided approach.3D images of the patient's upper right maxilla were obtained using CT (DICOM) and intraoral scanning (STL). These datasets were integrated into virtual planning software to create a prosthetically driven implant planning and a mockup. Using the software, we designed a 3D-printed surgical guide. During surgery, an atraumatic tooth extraction was performed, followed by bone drilling through the guide. The implant was placed, and autogenous bone grafting was done. Lately primary closure has been achieved by using a connective tissue graft from the tuberosity. The patient is scheduled for the second stage in late September.

## Conclusions

 This case illustrates the effectiveness and accuracy of digitally guided immediate implant placement, emphasizing its potential to simplify the surgical process and enhance outcomes in dental restoration.

# Abstract: Surgical technique for correction of bone defects in the jaws using anteromedial tibial grafting

**Authors:** S. García, M. Ouazzani, M. Ruiz, I. Leco, S. Blanco, J. López-Quiles

**Affiliations:** *Máster de Cirugía bucal e Implantología Universidad Complutense de Madrid*

## Introduction

 The tibial diaphyseal block graft, which is extracted from its anteromedial region, was described by Albee in 1911, and first used in oral and maxillofacial surgery by Lebedinsky and Virenque in 1918 for mandibular reconstruction. It is not currently known to be a widely used technique. However, in our Department of Oral and Maxillofacial Surgery, this method has been used for 25 years to reconstruct bone defects in the maxillofacial region. The main aim of the tibial diaphyseal block graft is to restore the pre-existing anatomy. It has shown great potential with qualities of osteogenesis with minimal adverse reactions.

## Case Report

s: Two clinical cases are presented, the first one being a 42-year-old male patient with no previous pathology presenting with a severe maxillary bone defect. He reports having undergone two previous surgeries with different bone grafts in the area to be treated, both of which failed. Clinical radiology shows an arch-shaped bone defect of 1.80 x 1.20 mm with no palatal wall located between 22 and 26. The second one is a 45-year-old male patient with severe atrophy of the left maxilla, who could not be rehabilitated with conventional osteointegrated implants. In both cases, the surgical technique was performed using the patient´s own tibial diaphyseal block graft during the same surgical procedure and under local anaesthesia. This graft was fixed with micro screws and shaped to fit the maxillary region. Bovine hydroxyapatite chips and collagen membrane were placed.

## Conclusions

 The tibial diaphyseal block graft is an easily obtained graft with excellent results, providing large bone volume with low morbidity. Further studies are needed to better understand the microarchitectural changes during remodelling and to determine the optimal timing for implant placement and loading.

# Abstract: Fixed dental prosthesis on six zirconia implants for anterior maxillary rehabilitation with 10-year follow-up: A case report

**Authors:** P. Revuelta Cortés, S. García Rodríguez, M. Ouazzani Touhami, J. López-Quiles Martínez, B. Cortés-Bretón

**Affiliations:** *Postgrado de Especialización en Cirugía Bucal e Implantología, Universidad Complutense de Madrid*

## Introduction

 In vitro and in vivo studies have shown zirconia implants to offer a viable alternative to titanium implants. Bacterial plaque exhibits little affinity with zirconia and so provokes less inflammatory infiltrate, so it may be supposed that this characteristic will reduce the frequency of peri-implant disease. Moreover, several studies have observed that a minority of patients present clinical signs of titanium allergy. Another advantage of zirconia implants could be better esthetics when placed in anterior areas with a thin gingival biotype. For these reasons, when selecting an alternative to titanium dental implants, ytrium oxide stabilized zirconia (y-TZP) has become the material of choice. Despite all the properties that make it a material of great interest, clinical research involving.long term follow-up prosthodontic restorations supported by zirconia implants is limited.

## Case Report

 The following clinical case is presented as a maxillary rehabilitation with implant-supported fixed dental prostheses on six zirconia implants. A woman with impacted maxillary canines attended the dental clinic seeking a metal-free maxillary restoration. After the extraction of both impacted maxillary canines and the placement of autogenous bone graft, six two-piece zirconia implants with straight abutments were placed in the anterior maxilla. Two zirconia fixed dental prostheses with porcelain veneering, separated at the midline, were placed on these zirconia implants.

## Conclusions

 The 10-year follow-up monitored the adequate evolution of both implants and restorations with no signs of peri-implant disease.

# Abstract: Apical Surgery Versus Tooth Extraction and Implant Placement: A case report

**Authors:** D. Sánchez Cousiño, C. Arasanz Àvila, S. Sardañés Martínez, A. Torrejon Moya, J. López-López

**Affiliations:** *Faculty of Medicine and Health Sciences - Master in Medicine, Surgery and Oral Implantology - University of Barcelona*

## Introduction

 Nowadays, implant treatments are on the increase. Although correct osseointegration of implants in infected sockets has been observed after adequate premedication and decontamination of the surgical bed, the incidence of complications associated with this procedure has also increased.

## Case Report

 A 33-year-old female patient with no relevant medical history or known allergies comes referred from the Endodontics Masters to the Master of Medicine, Surgery and Oral Implantology to perform periapical surgery in 1.1. A chronic radiolucent apical lesion was observed, compatible with periapical granuloma in 1.1 endodontically treated. Reendodontics were performed and after 3 months it was observed that the lesion persisted with the associated fistula and it was decided to perform periapical surgery. The aim of the technique is to eliminate the infectious focus through curettage, apicoectomy, and retrograde obturation. The indications include endodontic failure and lesions larger than 1cm, among others. Survival of periapical surgery is between 43-93% with complications such as failure of periapical healing and coronary or root fracture. In contrast, it has been observed that survival of immediate implant placement in an infected socket shows no difference with implant placement in a healthy socket, with a survival between 92-100% and shows complications such as mucositis in more than 50% of patients, fractures or peri-implantitis, which affect 28-56% of patients.

## Conclusions

 Although the survival rate of implants seems to exceed that of periapical surgery it is essential to consider the possible complications and assess periapical surgery to preserve the tooth.

# Abstract: Comparison of two procedures for the management of soft tissues around dental implants. A purpose of two cases

**Authors:** S. Llanos Chamorro, A. Serra Ristol, M. Fuentes Ortiz, E. Willaert Jiménez, P. Barenblit Schenin

**Affiliations:** *Máster Clínico en Implantología y Prótesis Oral, Universidad de Barcelona*

## Introduction

 The presence of keratinized tissue around dental implants increases long term stability while preventing peri-implant diseases. When considering an implant rehabilitation, periodontal conditions must be evaluated. The preservation or restauration of keratinized tissue must be considered, as well as knowing which technique is the most suitable for each case and which is the timing to perform it. Two surgical approaches were performed were the gain of keratinized tissues, the volume of soft tissues and the increase of buccal depth were assessed.

## Case Report

 Two patients with dental implants with a lack of keratinized tissue and presence of alveolar mucosa tension were treated using two surgical procedures. One case is treated with an apical repositioning flap and in the other case the apical repositioning flap is combined with a free gingival graft.

## Conclusions

 There is no evidence to recommend a specific technique for increasing the amount of keratinized mucosa. Both surgical interventions showed similar efficacy in terms of buccal tissue release and buccal depth. The combination of apical displacement flap and free gingival graft showed greater gain of keratinized around dental implants.

# Abstract: Surgical management of sinus pseudocystic pathology: a case report

**Authors:** A. González García, K. Ivaylova Serkedzhieva, D. Sahli, S. De la Vega Buró, L.M. Sáez Alcaide

**Affiliations:** *Departamento de Cirugía e Implantología, Facultad de Odontología, Universidad Complutense de Madrid*

## Introduction

 Sinus pathologies can significantly impact the outcomes of implant treatments. These conditions are quite common, and addressing them often requires the involvement of additional healthcare professionals, resulting in increased costs and delays in implant procedures. This article not only reviews the current scientific literature but also presents a clinical case of a maxillary sinus pseudocyst and explores an alternative method for its concurrent surgical management during the bone regeneration process necessary for implant rehabilitation.

## Case Report

 A 46-year-old patient presented with a pseudocystic lesion in the maxillary sinus was referred for implant rehabilitation in the regions of teeth 1.4 and 1.7. The patient experienced mobility and pain associated with a tooth-supported fixed prosthesis in the right maxilla. The surgical intervention, which included tooth extractions, maxillary sinus lift, and removal of the pseudocystic lesion, was performed in a single session.

## Discussion

: The timing of sinus surgery in relation to dental procedures remains as controversial matter of discussion. Some research supports the efficacy of simultaneous endoscopic surgery and removal of the odontogenic source, while other studies suggest that the sequence of sinus surgery first or dental treatment first does not affect the cure rate.

## Conclusions

 Effective diagnosis and surgical planning must consider both the specific characteristics of the pathology and the patient's treatment requirements. Managing sinus pathologies concurrently with bone regeneration procedures offers a safe and advantageous approach for patients undergoing implant rehabilitation.

# Abstract: Immediate Implant Placement and Provisionalization in the Anterior Sector. A Case Study

**Authors:** P. Martinez, N. Manzanares Mayandia, E. Willaert Jiménez, P. Barenblit Schenin

**Affiliations:** *Universidad de Barcelona*

## Introduction

 The placement of dental implants to restore natural aesthetics in the anterior region after tooth loss has become one of the greatest challenges in restorative dentistry. The fundamental requirement leading to the birth of this procedure is associated with the possibility of shortening treatment times and, above all, the need to preserve alveolar structures, which can undergo varying degrees of atrophy. The success of immediate provisionalization depends on careful case selection, a deep understanding of the biological principles of soft tissues and precision in the surgical manoeuvres involving them, and knowledge of the stages of bone repair.

## Case Report

 A 70-year-old female patient presented with trauma to tooth 22, leaving only the cervical third intact, with extraction being the only alternative. The general clinical condition of the soft and hard tissues was evaluated for the possible placement of an implant and immediate provisionalization. Radiographically, a deficient vestibular and palatal wall was observed, with a width of 6.40 mm from mesial to distal and sufficient height of 14 mm for three-dimensional implant planning.

## Conclusions

 The subsequent results after implant placement and immediate provisionalization in the aesthetic anterior sector of the upper jaw favour various aspects. On one hand, the maintenance of alveolar closure and the protection of the biomaterials placed inside result in predictability regarding the degree of success. Therefore, we can conclude that this is a technique that can be applied and recommended. We will continue to conduct long-term studies.

# Abstract: LPRF in the Treatment of Medication-Related Osteonecrosis of the Jaw around dental implants: A Case Series

**Authors:** A. Medeiros Monzón, C. Barrero López, P. Cea Arestín, F. Otero Pose, S. Rodríguez Zorrilla, A. Blanco Carrión

**Affiliations:** *Unidad de Medicina Oral (medoralres). Universidade de Santiago de Compostela*

## Introduction

 Medication-related Osteonecrosis of the Jaw (MRONJ) is a serious post-implantation complication in patients undergoing treatment with bone-modifying agents. LPRF (leukocyte- and platelet-rich plasma), promotes tissue and bone regeneration, improves healing, and reduces inflammation. This case series presents three patients with osseointegrated dental implants and MRONJ who were treated with surgery and LPRF.

## Case Report

 CASE 1: A 68-year-old woman treated with oral alendronic acid for osteoporosis developed peri-implantitis, which progressed to MRONJ in the third and fourth quadrants, stage II. Two surgeries were performed, including sequestrectomy and explantation of four implants, followed by LPRF application. Follow-ups showed good healing, though complications such as suppuration and bone exposure in the third and fourth quadrants persisted. Successive debridements and LPRF applications were carried out, resulting in complete healing. CASE 2: A woman treated with zoledronic acid for six years for multiple myeloma presented with stage II MRONJ in the third quadrant, associated with an implant. Explantation, sequestrectomy and LPRF application were performed, and subsequent follow-ups showed favorable progress. CASE 3: A woman presented with stage II MRONJ in the second quadrant due to prior use of zoledronic acid and pertuzumab for metastatic breast carcinoma. Sequestrectomy associated with the explantation of two implants in the second quadrant was performed, followed by LPRF application. Follow-ups showed favorable progress with progressive improvement in inflammation and graft vascularization, culminating in complete recovery at six months.

## Conclusions

 The treatment with LPRF for MRONJ around implants showed promising results as a coadjuvant to surgery. In the three cases, the use of LPRF post-sequestrectomy enhanced healing, minimized inflammation, and stimulated bone regeneration, leading to full recovery.

# Abstract: Importance of immediate implant provisionalization in the esthetic sector: a case report

**Authors:** C. Arasanz Àvila, L. Dumanova, R. Aceves Argemi, A. Mari Roig, M.L. Santaló, J. López-López

**Affiliations:** *Master's in Oral Medicine, Surgery, and Implantology. Faculty of Medicine and Health Sciences. University of Barcelona*

## Introduction

 Osseointegration in dental implants has become a reliable procedure; therefore, the objective has now shifted from ensuring osseointegration to achieving an appropriate aesthetic appearance. The aim of immediate provisionalization is to maintain initial aesthetics by preserving the architecture of soft tissues.

## Case Report

 Female patient, 42 years old, with a history of latex allergy and no other relevant medical history, has been referred from the endodontics department for the extraction of tooth 1.2 due to a perforation. Tooth 1.2 was extracted and a, 3.4 x 10mm BioHorizons® implant was immediately placed using the "eggshell" provisional technique . The emergence profile was created following the design of the 3 zones; E (aesthetic), B (bounded zone), C (crestal zone), using Protemp and Flow composite. It was adjusted to leave it in infraocclusion, and the gap was filled with xenograft. The patient was scheduled for follow-up appointments at one week, one month, and two months, during which the maintenance of the soft tissues was observed.

## Conclusions

 The emergence profile in immediate implants is crucial for maintaining the natural shape and contour of the gums, ensuring aesthetic integration with adjacent teeth. Finally, a well-designed emergence profile enhances patient satisfaction by meeting their aesthetic expectations.

# Abstract: Use of guided surgery for bone regularization and immediate implant placement.: A case Study

**Authors:** A. Sevilla Goytia, M. Fuentes Ortiz, X. Anton Lopez, E. Willaert Jiménez, P. Barenblit Schenin

**Affiliations:** *Clinical Master's in Implantology and Prothesis Buccal, Barcelona University*

## Introduction

 The guided surgery is a surgical planning procedure in which the patient is studied using radiographic technologies and advanced software, allowing for the planning and execution of procedures with millimetric precision. This reduces risks, enables less invasive procedures, and shortens treatment time

## Case Report

 A 55-year-old female patient presents for complete rehabilitation. She exhibits RR 13, 15, 16, 26, 27, in addition to lower partial edentulism with dental fractures from 34 to 44. Radiographic examination shows sufficient residual bone at the mandibular level for implant placement. The treatment plan involves a 100% guided intervention for greater efficiency. Three surgical guides were created: the first surgical guide for the positioning and extractions of teeth 34 to 44; the second surgical guide for bone regularization; and finally, the surgical guide for implant placement. To perform this procedure, 3D images of the patient's jaw (CT, DICOM) and an intraoral scan (STL) were obtained and processed with virtual planning software, allowing us to create these three surgical guides tailored to our need for mandibular bone regularization for subsequent immediate implant placement in the ideal position.

## Conclusions

 Performing 100% guided surgical procedures allows for a thorough preliminary study of the case, enabling precise planning and thereby executing the intervention with greater accuracy, efficiency, and simplification of the surgical procedure.

# Abstract: Osseodensification in implantology using densah burs: indications and protocols

**Authors:** A. García Marqués, P. Benito López, A. Fuertes Merino, F. Pérez González, C. Meniz García, C. Madrigal Martínez-Pereda

**Affiliations:** *Facultad de Odontología, Universidad Complutense de Madrid*

## Objectives

 The objetive of this case is to analyze the available scientific evidence regarding the advantages and disadvantages of using Densah burs to carry out different surgical protocols.

## Material and Methods

 a bibliographic search was conducted through PubMed, Cochrane, and Google Scholar. Systematic reviews and clinical studies published in the last 10 years on the osseodensification technique were included. The keywords used for the search were "osseodensification," "densah bur," "sinus lift," "split crest," and "systematic review."

## Results

 after thoroughly reviewing the articles found, the indications, protocols, advantages, and disadvantages of using Densah burs are presented, illustrated with clinical cases.

## Conclusions

 primary stability is an essential factor for the success of implant placement. This has been shown to improve thanks to the osseodensification technique, specifically through the use of Densah burs, as well as the bone height in atraumatic sinus lifts and the width of the ridge in crest expansions. In conclusion the osseodensification technique is a valid alternative for increasing bone tissue in terms of density, width, and height.

# Abstract: Oral rehabilitation with dental implants, into microvascular free radiated fibula flaps, after ablative squamous cell carcinoma surgery. Case report

**Authors:** J. Sousa, F. Guerra, S. Rocha, P. Dias, I. Cabo, T. Lopes

**Affiliations:** *Universidade de Medicina de Coimbra*

## Introduction

 Oral cavity squamous cell carcinoma is the most common malignancy of the oral cavity and includes subsites buccal mucosa, floor of mouth, anterior tongue, alveolar ridges, retromolar trigone, hard palate, and inner part of lipsis, being categorized under head and neck cancer. Reconstruction of large defects and complete oral rehabilitation for an optimal quality of life, mainly after ablative oncologic surgery in the maxillofacial region, assisted with radiotherapy and chemotherapy, is a desirable but challenging and difficult goal to achieve. Oral rehabilitation in these complex cases requires a multidisciplinary approach, planning and execution, in order to obtain the best possible outcome, in favor of the Patient's interest and quality of life.

## Case Report

 In this clinical case, due to the existing conditions, the therapeutic option of choice was a mandibular overdenture supported by a dolder bar on dental implants, placed into free autologous fibula transplants, and rehabilitation of edentulous spaces in the maxilla using implant-supported ceramic crowns.. 70-year-old caucasian male patient, retired, currently medicated with provastatin and acetylsalicylic acid. In 2017 suffered a stroke and simultaneously was diagnosed with oral cavity squamous cell carcinoma. in 2018 ablative oncologic surgery was performed in the affected maxillofacial region, assisted with radiotherapy and chemotherapy at the Portuguese Institute of Oncology, and reconstructed by the Maxillofacial Surgery team of the Coimbra University Hospital (Drª Teresa Lopes), replacing mandibular bone by free autologous fibula flat autogenous transplants and microvascular anastomosis, and soft tissues rehabilitated with free tissue autogenous grafts. Because the stomatognathic system has a variety of complex functions including mastication, speech, and deglutition, surgical resection of critical areas resulted in deleterious effects on this anatomy and physiology affecting functional patient outcomes, and due to the complexity of the case, extensive tissue destruction as well as biological limitations to surgical rehabilitation, it was not possible to reestablish the left lip closure, neither the reproduction of the vestibule and buccal mucosa, being unable to seal and close the lips and consequent difficulty in containing solids and liquids when chewing and swallowing, beside phonetic difficulties and compromised aesthetics. Due to these conditions, the Patient's oral rehabilitation was achieved in close collaboration between the Postgraduate Department in Oral Rehabilitation of the Faculty of Medicine of Coimbra (Professor Doctor Fernando Guerra; Professor Dr. Salomão Rocha; Dr. Jorge Sousa), jointly with the HUC Stomatology Service (Dr. Ivan Cabo) in order to optimize oral prosthetic rehabilitation to compensate for deficient situations, focusing on the best interests of the Patient and their quality of life. In 2023, after clinical evaluation and radiological analysis, the therapeutic option and clinical plan consisted of rehabilitation using an implant-tooth supported metalloacrylic lower overdenture, retained in the 3rd quadrant by a Dolder bar on two implants.

# Abstract: Guided bone regeneration with cortical lamina technique: A case report

**Authors:** B.J. Cárdenas Parada, V. Schiavo, A. Torrejón Moya, X. Roselló Llabrés, A. Marí Roig, J. López-López

**Affiliations:** *Master of Medicine, Surgery and Oral Implantology. University of Barcelona - Campus Bellvitge, C/ Feixa Llarga, s/n, 08907 L'Hospitalet de Llobregat, Barcelona*

## Introduction

 In implant rehabilitation, adequate alveolar bone integrity and volume are essential to achieve success in any clinical situation. Various grafting techniques and materials have been described to achieve favorable situations for rehabilitation. Grafts can be autologous, homologous or heterologous. Autologous grafts have osteoinductive, osteogenic and osteoconductive capacity; however, they require a donor site. Heterologous ones require a single surgical access, reducing postoperative discomfort and morbidity for the patient, but only present osteoconductive activity; therefore, the combination of both types has been proposed, with the simultaneous use of resorbable or non-resorbable membranes, to give stability to the graft. Collagenized porcine cortical bone lamina has favorable biological and mechanical properties to successfully achieve hard tissue augmentation. The objective of this clinical case is to describe the technique with cortical lamina as a predictable technique prior to rehabilitation treatment.

## Case Report

 62-year-old woman, no allergies, with a history of osteoporosis, medicated with Teriparatide®, smoker of 4 cigarettes/day. She came for evaluation of implant-supported rehabilitation in the second (2Q) and fourth quadrant (4Q). In 2Q with horizontal and vertical bone defect (2.28mm); given the low bone availability, it was decided to elevate the maxillary sinus floor with lateral window, simultaneously with guided bone regeneration technique (GBR) using cortical lamina technique (Osteogenos®) with 50% autologous bone filling and xenograft (50% Apatos®) and deferred implant placement. Sinus elevation and GBR were performed, and after 6 months the patient was rehabilitated with implants in position 2.5, 2.6, 2.7, 4.7.

## Conclusions

 GBR with autologous/heterologous bone substitute combined with cortical lamina represents a predictable option to increase bone volume in edentulous ridges with atrophic defects, with great biocompatibility, stability and reduced postoperative morbidity, allowing the recreation of sufficient bone volume to place and restore implants.

# Abstract: Immediate implantation with use of ctg for defect closure: a case report

**Authors:** K. Pineda Medina, M. Fuentes Ortiz, E. Willaert Jimenez, P. Barenblit Schenin

**Affiliations:** *Master of Implantology and Oral Prosthetics, University of Barcelona*

## Introduction

 Current studies show that the immediate placement of implants after dental extraction obtains better long-term results. Among its advantages are reducing bone resorption of the post-extraction socket, shortening the rehabilitation treatment time and avoiding a second surgery. of implementation. On the other hand, drawbacks include generally requiring membrane-guided bone regeneration techniques, with the risk of exposure and infection; and the need for mucogingival grafts to close the socket and/or cover the membranes.

## Case Report

 A 73-year-old male patient comes to the clinic with a coronal fracture of the 15th leg and pain. His medical history includes renal failure and type II diabetes mellitus, controlled with his specialist doctor, who underwent atraumatic tooth extraction of OD#15 with immediate placement of a dental implant, ROG and connective tissue graft taken from the edentulous area of the tuberosity for defect closure; whose procedure was carried out without complications and with correct position and parallelism.

## Conclusions

 It should be an important factor to consider that premature exposure of the membrane can lead to complications, such as infection, bone loss or loss of the implant, compromising the predictability of immediate implants. In these cases, it is necessary to ensure a stable tissue, sufficiently thick and well vascularized, sutured without tension, completely covering the membrane and completely closing the soft tissue defect.

# Abstract: Inmediate implant placement: when less is more

**Authors:** B. López López, V. Casanova Martínez, L. Kathleen Bragança, C. Ayala Paz, J. Pato Mourelo, E. Velasco Ortega

**Affiliations:** *Máster de Implantología Oral. Universidad de Sevilla*

## Introduction

 Endosseous dental implants have excellent long-term success rates. In addition to restoring function and intraoral biology, esthetic considerations are key when replacing teeth in the anterior region. The ultimate goal is to replace missing teeth with implantl restorations indistinguishable from the surrounding teeth in terms of shape, morphology and color, but also to restore and maintain a natural hard and soft tissue, which is considered the framework for any restoration.

## Case Report

 A male patient, smoker but with good general health, with a significant economic limitation, comes to the consultation wanting to replace a fractured 21. After initial radiographic testing including orthopantomography and CBCT, sufficient bone availability is observed for immediate implant placement. Aware of the economic limitation, a treatment plan is proposed where the minimum essential will be carried out to obtain an optimal result. The extraction is performed and a Galimplant IPX 4010 implant is inserted; The socket gap is filled with a beta-tricalcium phosphate (Osteoblast) and a Galimplant MUSA 04030 anti-rotational abutment. Digital impressions are taken using the AoralScan (Shining 3D) intraoral scanner. A provisional is digitally designed and printed. It was screwed to the abutment and the implant and after 3 months, new digital impressions were taken to create a piece of stratified monolithic zirconia. Six months after definitive rehabilitation, no complications were observed.

# Abstract: Treatment of gingival recessions with connective tissue graft vs tissue substitutes

**Authors:** H. Lobaina Panero, J. Espinosa Gonzalez, A. Peciña Melgosa, M. Del Canto Diaz, R. Pérez Bryan

**Affiliations:** *Master de Cirugía Bucal, Implantología y Periodoncia de la Universidad de León*

## Objectives

 This bibliographic review evaluated the effectiveness of different alternatives to connective tissue in surgical procedures of covering root, in the treatment of gingival recessions. MATERIAL AND 

## Material and Methods

 A search was carried out in PUBMED in the last 5 years. 1st search with keywords gingival recession + connective tissue graft + substitutes matrix; 2nd search with gingival recession + connective tissue + acellular dermic matrix; 3rd search with gingival recession + connective tissue or dermic acellular matrix; 4th search with gingival recession + dental esthetic + matrix; 5th search acellular dermal matrix + connective tissue graft + gingival recession + tooth cervix + dental esthetics + patient satisfaction.

## Results

 We included 25 randomized controlled trials (RCTs), most of 3, 6, and 12 months in duration, that evaluated RT1 and RT2 gingival recession treated by root coverage procedures. The results indicated greater success in root coverage with the connective tissue graft (CTG), followed by allogeneic acellular dermal matrix with an average root coverage rate of up to 95%; followed by xenogenic dermal matrix with RRM rate of up to 92.6%; and xenogenic collagen matrix with RRM rate of up to 60%.

## Conclusions

 Connective tissue grafts are the gold standard for treatment of cover gingival recessions; with gain of keratinized gingiva and aesthetic level. Although "It is not without complications". We must not forget certain drawbacks, such as postoperative discomfort, having two surgical areas, longer clinical work time, and limited donor tissues.However, allogenic acellular dermal matrix is a good alternative when autologous tissue is not available.

# Abstract: Bioactive surfaces in dental implants. Systematic review of the literature

**Authors:** P. Ruiz García, H. Álvarez Álvarez, V. Laguna Tisaire

**Affiliations:** *Private dental clinic in Oviedo (Asturias)*

## Introduction

 Bioactive surfaces are those with the ability to interact with the peri-implant bone to accelerate osseointegration enableling prosthetic rehabilitations as short as possible.

## Objectives

 Evaluate the different types of bioactive surfaces comparing with conventional titanium.

## Material and Methods

 The PUBMED database has been used with the following keywords: "dental implants", "osseointegration", "bioactive surfaces", "coating techniques", including articles published in the last 5 years written in English. From the 103 articles found, 43 were less than 5 years old. Finally, after reviewing the relevance for this study and its impact, we selected 8 articles.

## Results

 Significant differences in bone-implant contact (BIC) were found between SLA Implants and SLA modified with bioactive peptide SLA (43.62+- 10.79) and RGA-SLA (61.68+-4.21). In another study the new bone formation at 8 and 12 weeks in implants with polished and sandblasted surface (34.96+-1.23 vs 42.04+-1.24, p<0.05. No significant differences were observed in BIC between sandblasted and calcium phosphate-coated titanium surfaces (Lopez-Velarde et al). Pabst et al compared 3 surface treatments, finding that hydrophilic etching (ANU) presented a higher BIC in the first week (99.91). compared to etching-sandblasting (SA); (95.85) and etching (AN).

## Discussion

: We can distinguish, on the one hand, bioactive coatings, such as growth factor concentrate, calcium phosphate and hydroxyapatite, and on the other hand, physical and chemical treatments which modify the topographic structure of the implants to increase roughness and BIC. In most of the articles reviewed, bioactive surfaces in dental implants significantly improve and accelerate the osseointegration and stability of the implants, although there is no consensus about which has a higher level of osseointegration, which should be the subject of future research. 

## Conclusions

 The studies reviewed confirm that bioactive surfaces can reduce osseointegration times and be an alternative to conventional titanium surfaces.

# Abstract: Effectiveness of Platelet-Rich Fibrin in Maxillary Sinus Augmentation

**Authors:** R. Rodríguez Lezcano, P. Portela Monteagudo, B. Shafi Akhtar

**Affiliations:** *Master de Implantología y Periodoncia Universidad Isabel I Escuela Internacional de Postgrado Odontología*

## Objectives

 This poster aims to assess the effectiveness of the use of Platelet-Rich Fibrin (PRF) in maxillary sinus lift in dentistry

## Material and Methods

 The PubMed database was used, performing a systematic search, using an appropriate search equation, within the eligibility criteria.

## Results

 After searching through the Mesh terms, a total of 69 articles were obtained, of which 9 were selected to be included in this poster.

## Conclusions

 There is a majority consensus that PRF is beneficial for the regeneration of hard tissues in dental procedures, as well as the favorable behavior of clinical parameters after its application, however, several contradictory results among the authors do not show it to be far superior to other regenerative therapies, so more studies on the subject would be necessary.

# Abstract: Platform switching VS Platform matching. A systematic review

**Authors:** P. Rullo Fernández, M. Petralanda Lopez, J.M. Saborido Martín, C. Barrilero Martín, M.A. Alobera Gracia

**Affiliations:** *Máster de cirugía bucal, implantología y periodoncia de la Universidad de León*

## Introduction

 In the 1990s, dental implants with wide platforms of 5-6 mm in diameter emerged. However, the prosthetic abutments used until now were smaller in diameter. Subsequent clinical and radiographic control demonstrated that the use of this form of connection better preserved the crestal bone around the implants.

## Objectives

 To carry out a bibliographic review of studies that compare the peri-implant radiographic bone level in patients with implants without platform reduction (Platform Matching) and implants with platform reduction (Platform Switching).

## Material and Methods

 A bibliographic search on the use of implants with reduced platform was carried out in the Pubmed and Google Scholar database, selecting articles that met the selection criteria and published in English. Clinical cases, experimental studies, meta-analysis and literature review were included in the search.

## Results

 The results of the studies show that there is less bone resorption in implants with a reduced platform compared to implants with a conventional platform.

## Conclusions

 It has been shown that the use of platform change prevents peri-implant-crestal bone loss. However, more long-term studies are needed to confirm these results.

# Abstract: Use of PRF membranes in the osseointegration of dental implants

**Authors:** Y. Garcia Sanjuan, C. Benet Salido, S. De Dios Corcoles, M. Arabi Tihlili, A. Boquete, S. Cacciacane

**Affiliations:** *Master in oral surgery, implantology and oral rehabilitation Esiro Barcelona, UEMC*

## Objectives

 The objective of the systematic review is to describe the influence of the use of PRF membranes on the osseointegration of dental implants placed in adult patients.

## Material and Methods

 A systematic review has been carried out through an information search using keywords and the identification of studies in Pubmed, Scopus, Web of Science and Biblioteca Virtual de Salud. Terms have been used for the search strategy and the inclusion and exclusion criteria of the studies have been defined.

## Results

 6 articles were identified that met the inclusion and exclusion criteria.

## Conclusions

 Crucial to achieving successful bone regeneration is bone filling in the gap between the implant and the bone using PRF membranes in conjunction with particulate bone grafts or bone substitutes or a combination of both; the migration of pluripotent and osteogenic cells, such as osteoblasts, towards the bone defect; the exclusion of cells that could prevent bone formation such as epithelial cells and fibroblasts. The success of PRF membranes is also due to their high content of growth factors, such as the autologous fibrin matrix rich in leukocytes and platelets, and their three-dimensional structure containing cytokines and stem cells. Cytokines have important defense capabilities against infections, they have a crucial anti-inflammatory and antibacterial function during the process of immune regulation and angiogenesis, therefore, they are essential in bone regeneration processes in dental implants.

# Abstract: Artificial intelligence in implantology, a systematic review

**Authors:** M. Castañal Pérez, G. Banchero Svetlich, R. Señor García, R. Mateos Rodríguez, J. Pesquero Velasco

**Affiliations:** *Máster Universitario en Cirugía Bucal, Implantología y Periodoncia de la Universidad de León*

## Objectives

 To determine whether AI is a valid and reliable method for identifying the brand and type of implant from a conventional panoramic radiograph or digital periapical radiographs.

## Material and Methods

 A systematic review was conducted in three different databases: MEDLINE/PubMed, Google Scholar and Scopus. The inclusion criteria were articles from journals included in the JCR since 2020 whose topic was artificial intelligence and implants. As exclusion criteria, letters or expert opinions on the applications of AI in dentistry were eliminated.

## Results

 9 articles were included in this review after eliminating those that did not satisfy the criteria. All of them have shown that AI is an effective and useful method to detect both the brand and type of implant from panoramic radiographs or periapical radiographs. However, we need to carry out further research to keep developing and evaluating the clinical accuracy of AI.

## Conclusions

 AI seems to be an efficient method to determine implant type and brand from previous radiographs, but further studies to confirm this and further development of its clinical accuracy are required.

# Abstract: Comparative Analysis of Mechanical Complications, Color Stability, and Patient Perception of Polyoxymethylene (POM) versus Polymethylmethacrylate (PMMA) for Screw-Retained Temporaries on Implants: A Systematic Review

**Authors:** J. Salazar Cantero, P. Truchuelo, E. Regidor, A. Ortiz-Vigón, M. Alobera, M. Del Canto

**Affiliations:** *Máster en Cirugía Oral, Implantología y Peridoncia. Universidad de Léon*

## Objectives

 The present systematic review aims to compare the mechanical complications, color stability, and patient perception between the use of polyoxymethylene (POM) and polymethylmethacrylate (PMMA) for screw-retained temporaries on implants.

## Material and Methods

 The review was carried out following the PRISMA (Preferred Reporting Items for Systematic Reviews and Meta-Analyses) guidelines. The PubMed, Scopus, Cochrane Library, and Clinical Trials databases were searched, covering the period up until June 15th, 2024.

## Results

 Three studies fulfilled the inclusion criteria. Among these, only one was a randomized clinical trial. Fracture rates were observed in both materials, reaching up to 28% for PMMA and 12.5% for POM. The differences in mechanical properties and color stability did not reach statistical significance (Chi-Square Test, P=0.178),(Mann-Whitney test, P=0.595). No study analyzed patient perception.

## Conclusions

 Within the limitations of this review, no statistically significant differences were observed in mechanical complications and color stability. The certainty of the evidence for these outcomes was very low, and there is no available evidence on the impact of the materials on patient perception.

# Abstract: Iatrogenic Sinusitis Secondary to Dental Implant Treatment

**Authors:** C. Salido Benet, S. Cacciacane, A. Boquete Castro, Y. García Sanjuan

**Affiliations:** *Esiro, Universidad Europea Miguel de Cervantes*

## Objectives

 The purpose of this systematic review, following the PRISMA methodology, was to analyze the scientific evidence on the treatment, prevention methods, and factors involved in iatrogenic sinusitis.

## Material and Methods

 A total of 131 documents were collected in 3 search collections in the PubMed, Science Direct, and Scielo databases, of which 3 documents were selected according to the inclusion and exclusion criteria. Additionally, one document that does not meet the eligibility criteria but provides relevant information for the research was included.

## Results

 The results indicate that within the management protocols for iatrogenic sinusitis, the determination of the absence of an oroantral fistula is essential before making any prosthetic rehabilitation decisions. The authors found that sinusitis secondary to dental implant treatment had an iatrogenic origin in 65.7% of cases, 25.1% resulted from apical periodontitis, and 8.3% resulted from marginal periodontitis. The data also identified other etiological factors: chronic polypoid rhinosinusitis, benign tumors, and allergic fungal sinusitis.

## Conclusions

 Sinusitis secondary to dental implant treatment is differentiated from others in terms of microbiology and pathophysiology, making it crucial to manage them therapeutically different from those of rhinosinusitis origin. The authors indicate that treatments typically involve the removal of implants and subsequent restoration of the bone plane using an autologous bone graft from the chin in certain cases. However, the treatment must be interdisciplinary and include functional endoscopic surgery. The management of iatrogenic sinusitis secondary to dental implant treatment involves both the maxillofacial surgery team and the otolaryngology team, making their collaboration essential. This cooperation facilitates the handling of the pathology and produces favorable outcomes.

# Abstract: Evaluation of Bone Gain and Implant Survival Rates after Guided Bone Regeneration with Prefabricated Titanium Foils versus Customized Titanium Membranes: A Systematic Review

**Authors:** A. Serrano, S. Cacciacane, A. Boquete

**Affiliations:** *Escuela Superior de Implantología y Rehabilitación*

## Introduction

 Adequate bone volume is an essential requirement for attaining functional and aesthetic outcomes in oral implantology. Guided bone regeneration(GBR) techniques are widely used to treat localized jawbone defects because of their low technical demands and substantial osteogenic effect. Barrier membranes prevent soft tissue invasion from the mucosa and create a space underneath to facilitate such bone growth.Commercial titanium foils have been traditionally used due to their biological properties and favorable bone regeneration outcomes,however,they often fall short on clinical requirements such as space preservation. With the advent of digitalization and three-dimensional printing,technology has allowed the creation of customized titanium barrier membranes that offer a more accurate bone graft. This systematic review aims to compare the success rates of prefabricated meshes and customized membranes in terms of bone gain and the survival rate of the implants placed afterwards in the regenerated area.

## Material and Methods

 A digital literature search was performed in two databases-PubMed and Scopus-for randomized clinical trials (RCT),case reports and case series,that included GBR techniques using commercial titanium meshes or customized titanium foils and a minimum of a six-months follow-up.Using the PICOS principles to help determine inclusion criteria,articles published in English or Spanish in the last 15 years were included.The Strenght of Recommendation Taxonomy (SORT) and Cochrane Collaboration's tool (RoB 2) criteria were used to assess de quality and risk of bias of the articles.

## Results

 A total of fifteen articles that measured horizontal and/or vertical bone augmentation were finally included in the qualitative analysis.In eight of the studies,implants were placed in the regenerated bone and their survival rate was reported.

## Conclusions

 Bone gain seems to be slightly greater when using prefabricated meshes rather than customized foils.The survival rate of the implants placed in the regenerated area is similar to the rate of implants located in native bone.

# Abstract: Maxillary sinusitis secondary to treatment with zygomatic implants

**Authors:** F. Martínez Cano, B. Vila Regueira, I. de la Osa Castrillo, M. Sevilla Hernández, M. del Canto Pingarrón

**Affiliations:** *Máster en Cirugía Bucal, Implantología y Periodoncia. Universidad de León*

## Introduction

 The placement of zygomatic implants is a technique described in 1988 by Bränemark and since then different techniques have emerged to address the treatment. Currently, it is a consolidated technique in the treatment of patients with large bone resorptions, but like any surgical technique it presents a series of complications that must be taken into account.

## Objectives

 The objective is to review the existing literature on the prevalence and causes of maxillary sinusitis in patients treated with zygomatic implants. Material and methods: A systematic bibliographic search of articles published in the last 5 years was carried out in the PubMed and Medline databases following the PRISMA recommendations. The key words used were: zygomatic implants, zygoma and maxillary sinusitis.

## Results

 Studies cite that maxillary sinusitis is the most common complication with a prevalence of 8%, although it is much more common in the intrasinus technique than in extrasinus techniques.

## Discussion

: The perforation of Schneider's membrane, the surface of the implants and the existence of previous sinusitis are factors that influence the appearance of sinusitis, so to avoid this complication, extrasinus placement of the implants is preferable.

## Conclusions

 Sinusitis is the most common complication in treatment with zygomatic implants, and although its prevalence is relatively low, a detailed study of each case must be carried out to try to avoid its appearance.

# Abstract: Peri-Implant Tissue Challenge in the Anterior Sector

**Authors:** C.A. Sánchez Pena, L. Rivero Mourelle, F. Sanclemente Reina, C. Jacobo Orea, M.A. Alobera Gracia, M. del Canto Pingarrón

**Affiliations:** *Master's in Oral Surgery, Implantology, and Periodontics, University of León*

## Introduction

 The use of implants in the anterior sector is a highly reliable treatment option. Immediate implants reduce healing time while maintaining high success rates. Factors such as dimensions, insufficient keratinized mucosa (<2mm), gingival biotype, and papilla formation can be risk indicators.

## Objectives

 To determine the appropriate management of peri-implant soft tissues to prevent complications in the anterior sector.

## Material and Methods

 A systematic literature search was conducted in the PubMed database. Keywords: dental implant, dental implants, aesthetic zone, anterior zone, soft tissue management in English, from 2014 to 2024. Initially, a total of 104 studies were obtained, from which, after applying inclusion and exclusion criteria, 10 were selected for our literature review.

## Discussion

 and 

## Results

 Immediate placement with provisionalization offers excellent results. Less distal papillary recession was observed when provisionalized. However, more randomized clinical trials are needed to confirm this. Connective tissue grafts (CTG) are frequently used in combination with immediate implant placement and provisionalization to enhance the outcomes. Even under ideal conditions, there is a risk of recession when a ITC is not performed, so the presence of keratinized gingiva is crucial.

## Conclusions

 The placement of an immediate provisional prosthesis along with a ITC enhances both aesthetics and the prevention of soft tissue complications. Nonetheless, more long-term studies are necessary to definitively determine the treatment plan.

# Abstract: Free gingival graft to increase attached gingiva and keratinized mucosa

**Authors:** J. España Guerrero, A. España Guerrero, E. España Guerrero, G. Moreu Burgos, A. España López

**Affiliations:** *Graduate in Odontology. UEM*

## Introduction

 Free gingival graft is one of the mucogingival surgery techniques to increase the amount of inserted and keratinized gingiva, improve biological sealing and provide tissue stability that favors the maintenance of the health of the tissues around teeth and implants.

## Objectives

 To evaluate whether free gingival grafts achieve a significant increase of attached gingiva in teeth and dental implants.

## Material and Methods

 A review has been carried out in the main electronic bibliographic databases MEDLINE (Pudmed), EBSCOhost analyzing studies in which free gingival grafts were performed on teeth or implants. A total of 241 articles and publications were found in the search, which have been included in the PRISMA workflow diagram to make the selection and all these articles were reviewed and 205 articles were excluded, and from the rest, the 18 most relevant articles were selected.

## Results

 The analysis of the literature review shows that most authors consider the free gingival graft technique as the Gold Standard for obtaining keratinized gingiva in teeth and implants. In the reviewed articles we have analyzed the comparisons that were established by the different researchers to evaluate variants or alternatives to this technique of Free Gingival Graft (FGG) as with maintenance-only control groups, comparison with the use of xenogeneic collagen membranes (XCM), and in other groups of studies analyzed adjuvant techniques such as botulinum toxin (BTA-X) or platelet-rich fibrin (PRF).

## Conclusions

 The free gingival graft is one of the most used methods to gain attached gingiva, obtaining a high percentage of success, considered the Gold Standard, maintaining its characteristics during a long follow-up period, with predictable results.

# Abstract: Guided bone regeneration versus sandwich technique

**Authors:** B. Cabrera Galeano, C. Espinoza Mendoza, C. Méndez Guerrero, J. Calatayud, A. Bowen

**Affiliations:** *Master´s Program in Implantology and Periodontology, Universidad Isabel I*

## Introduction

 Beside the horizontal bone loss following extraction, height is also reduced. Nowadays, various surgical procedures are available to correct these deficiencies for posterior implantrehabilitation. The aim of the present study is to compare the success rate of Segmental Sandwich Osteotomy (SSO) vs guided bone regeneration with PTF membrane in pre-implant surgery, focusing on the survival of theregenerated area in vertical loss cases.

## Material and Methods

 The search strategy involved searching theelectronic databases of MEDLINE, Pubmed, Embase, Scopus, Web of Science, Trip, Cochrane Oral HealthGroup's Trials Register, Cochrane Central Register of Controlled Trials y ProQuest Dissertations & Theses from2006 to the present day.

## Results

 Despite the extensive possibilities of boneregeneration, we should aim to perfectionate one or twotechniques. GBR appears to be the surgically easiesttechnique of the two studied in this review, even if it has more post-op complications. SSO is a sensitive techniquethat depending on the area could have unpleasant long-term complications.

## Conclusions

 When the aim is a vertical increase of alveolar bone, both procedures have stable and predictableresults. However, to choose between the two of them, willrely on thebuccal conditions and the clinical level of expertise.

# Abstract: Effectiveness of Platelet-Rich Fibrin in Maxillary Sinus Augmentation

**Authors:** R. Rodríguez Lezcano, P. Portela Monteagudo, B. Shafi Akhtar, E. Bueno, A. Bowen Antolín

**Affiliations:** *Master´s Program in Implantology and Periodontology, Universidad Isabel I *

## Objectives

 This poster aims to assess the effectiveness of the use of Platelet-Rich Fibrin (PRF) in maxillary sinus lift in dentistry.

## Material and Methods

 The PubMed database was used, performing a systematic search, using an appropriate search equation, within the eligibility criteria.

## Results

 After searching through the Mesh terms, a total of 69 articles were obtained, of which 9 were selected to be included in this poster.

## Conclusions

 There is a majority consensus that PRF is beneficial for the regeneration of hard tissues in dental procedures, as well as the favorable behavior of clinical parameters after its application, however, several contradictory results among the authors do not show it to be far superior to other regenerative therapies, so more studies on the subject would be necessary.

# Abstract: Dental implant stability and post-surgical healing and inflammation with Photobiomodulation

**Authors:** P. Akbari, G.C. Camolesi, J.M. Somoza Martín, M.D. Reboiras López, F. Camacho Alonso, A. Blanco Carrión, M. Pérez-Sayáns, P. Gandara Vila

**Affiliations:** *Grupo OralResMed.USC 2.(IDIS) (ORALRES) 3.Department of Oral Surgery, University of Murcia*

## Objectives

 Low-level laser therapy (LLLT) or photobiomodulation (PBM) is used to enhance the bone cicatrization process by reducing the healing time for osseointegration of dental implants (DI) in preclinical studies. This clinical trial aimed to evaluate the effect of 808 nm diode laser PBM on implant stability and the effect of 630 nm diode laser PBM on inflammation and post-surgical healing.

## Material and Methods

 Forty DI were inserted into 13 patients. The implants were randomly divided into two groups. The test group (PBM+) underwent two sessions of PBM (combined diode laser of 630 and 808 nm), first after surgery, and the second, 7 days after the surgical procedure. The control group (PBM−) received simulated laser treatment. The implant stability quotient (ISQ) was determined immediately after the surgical procedure, and 7 days, 4 and 8 weeks later. Post-surgical inflammation was assessed following the criteria described by Bloemen and Cols. Healing was calculated using the healing index (HI).

## Results

 No differences were found in terms of the mean values of implant stability between the test and control group. Two DI (18.2%) from the PBM− group were classified with the maximum HI(5), whereas in the PBM+ group were nine implants (45%). It was determined by logistic regression that the non-application of the laser in the PBM− group caused an OR of 4.333 times of presenting inflammation.

## Conclusions

 The application of 808 nm infra-red laser for bone tissue, and 630 nm for mucosal tissue in two sessions is considered to be an effective way of reducing inflammation and improving early healing.

# Abstract: Intelligent System for Bruxism

**Authors:** J. Espirito Santo, J. Dias, M. Seabra

**Affiliations:** *Medical Art Center*

## Objectives

 Bruxism is the term used to describe the repetitive activity of the masticatory muscles, characterised by teeth grinding or clenching. This study aimed to combine the positive aspects of two therapeutic techniques: Occlusal Plates and Biofeedback for Temporomandibular Dysfunction (TMD). Specifically, it sought to develop a bite guard capable of monitoring and alerting patients suffering from bruxism associated with TMD.

## Material and Methods

 The involuntary act of teeth grinding is detected using pressure sensors that convert movements into electrical voltage. This voltage is then sent to a microcontroller, where it is processed, filtered and stored, and then transferred to a PC for graphical analysis of the data.A significant innovation in this project is the inclusion of a digital patient diary (ddp). This diary makes it possible to record daily events associated with increased stress, such as meetings, work commitments or other challenging situations. The system cross-references this data with the biofeedback acquired by the smart bite guard, which monitors bruxism episodes. By analysing the correlation between events and teeth grinding activity, the patient can identify patterns and learn to control the urge to grind their teeth in stressful situations. This personalised feedback aims to educate the patient about the triggers of bruxism and promote behavioural strategies to reduce the occurrence of the problem.This study adopts an interdisciplinary approach that integrates concepts from dentistry and electronic engineering.

## Results and Conclusions

 The results of this prototype demonstrate the feasibility of the system developed. The pressure sensors inserted into the occlusal stabilisation plate were able to accurately and consistently detect bruxism episodes. Analysing the stored data revealed reasonable sensitivity and specificity parameters for the system, validating its effectiveness.

# Abstract: Minimally invasive techniques in implantology and bone loss

**Authors:** R. Frioui

**Affiliations:** *Centro odontológico en Paris*

## Introduction

 Dental implantology has significantly advanced in the recent decades. The use of implants for the rehabilitation of edentulous or partially edentulous patients is done regularly and has safe and predictable successful results. However, implant surgery continues to be an invasive and biologically aggressive surgical act, always causing inflammation at the surgical site and causing bone resorption around the implant. Bone loss was for a long time accepted and normalized until 2mm but more techniques and studies are made to preserve the most bone possible and implant success. Since the sixties, full-thickness mucoperiosteal flaps have been made to access the bone and place the implant and peri-implant bone loss and post-operative discomfort were related . Minimally invasive techniques emerged such as flapless surgery, opening the mucosa with only a minimal incision or with a punch drill appropriate to the diameter of the implant, or directly drilling the site, guided surgery

## Objectives

 Study the minimally invasives techniques in dental implantology study the effects of these techniques on bone loss Bibliographic review using data bases such as PubMed, Cochrane Library, Google Scholar

## Results

 Some studies have not proven a significant difference between using minimally invasive techniques and using the traditional techniques, some studies have proven that using minimally invasive techniques can prevent losing more bone after implants. Most of the studies found a lesser bone loss whether its marginal or due to bone resorption when these techniques were used.

## Conclusions

 Factors such as the surgeon's skill and experience, selecting the patient for the use of these techniques , quality and quantity of the bone have to be examined first, age, systemic diseases , oral hygiene, patient's economic abilities and cooperation of the treatment are influential to the outcome.

# Abstract: Effect of chlorhexidine mouthwashes on the microbiome and in vitro biofilm associated with peri-implantitis lesions

**Authors:** F. Pose Otero, A. Parga, P. Otero Casa, J.M. Pose Rodríguez, A. Otero Casal, M. Gallas Torreira

**Affiliations:** *Department of Surgery and medical surgical specialities. Stomatology Area .Universidade de Santiago de Compostela Department of microbiology and parasitology Universidade de Santiago de Compostela*

## Introduction

 Among the chemical methods used for the treatment of peri-implantitis (PI), the use of chlorhexidine (CHX) mouthwashes is the most frequently applied approach, although the effect of CHX on the subgingival microbiome of PI-lesions is not well characterized and needs to be assessed due to the undesirable side-effects derived of the prolonged use of this antiseptic.

## Objectives

 In order to evaluate the effect of CHX mouthwashes on the PI-associated microbiome.

## Material and Methods

 The samples obtained from PI lesions from 22 patients were analyzed before and after the use of 0.05% CHX (Perio-aid® maintenance, n=11), or 0.12% CHX, (Perio-aid® treatment, n=11) mouthwashes three times a day for a period of 15 days. microbiomes were analyzed by Illumina MiSeq sequencing of 16S rRNA genes.

## Results

 Despite the effect of the use of the commercial CHX mouthwashes on the bacterial diversity was clearly dependent on the patient, none of the CHX mouthwashes caused statistically significant changes in the PI-associated microbiome. The 0.12% CHX treatment caused a slight but not statistically significant reduction in the number of genus and species in the PI pocket in comparison with the 0.05% CHX mouthwash. A high prevalence of periodontopathogens including Aggregatibacter, Fusobacterium, Porphyromonas, Tannerella, Prevotella and Treponema was observed in the PI samples.

## Conclusions

 Although the analysis of a higher number of samples would be needed in order to achieve more robust conclusions, the 0.05% CHX mouthwash seems to be more effective in reducing the relative abundance of oral pathogens in the PI pocket. In the view of the limited effect of both CHX treatments on the peri-implantar microbiome and its indiscriminate antimicrobial activity and undesired secondary effects, its generalized use for the treatment of PI should be limited in the clinical practice.

# Abstract: Influence of loading and drilling on marginal bone loss around implants with a Dynamic Bone Management design: A single-blind, randomised, 12-month clinical trial

**Authors:** B.J. Pinzón Rodríguez, F. Seijas Naya, G.C. Vianna Camolesi, F. França Vieira e Silva, J.M. Somoza Martin, M. Pérez-Sayáns

**Affiliations:** *Máster Medicina Oral, Cirugía Oral e Implantología de la Universidad de Santiago de Compostela*

## Introduction

 Osseointegration is defined as the direct structural and functional connection between living bone and the surface of an implant. Primary stability is paramount to ensure proper osseointegration. Therefore, most companies in the industry are striving to design implants that ensure greater retention in the bone substrate, and have also developed drilling protocols capable of effectively achieving this primary retention.

## Objectives

 To evaluate marginal bone loss at 6 and 12 months after prosthetic loading of implants with Dynamic Bone Management (Straumann, Basel, Switzerland) by applying different drilling protocols.

## Material and Methods

 A single-blind, randomized, balanced, clinical trial was performed with four parallel experimental arms: immediate loading and underdressing, immediate loading and full drilling, early loading and underdressing, and early loading and full drilling. Forty-four implants were placed with a Dynamic Bone Management design with a diameter of 3.75 mm and a length of 10.00 mm in mature healed bone.

## Results

 The mean primary stability achieved was 60.6 ± 12.2 implant stability quotient, with a range of 21 to 75, and no differences were observed when considering the drilling protocol used, bone type or location. Early loading resulted in a bone loss of 0.728 mm, while immediate loading reflected no bone loss. When the interaction between loading and reaming protocols was studied, it was found that performing the full reaming protocol in conjunction with early implant loading resulted in less marginal bone loss, with a marginal bone gain effect of 0.814 mm.

## Conclusions

 Use of the full reaming protocol in conjunction with early implant loading resulted in the least marginal bone loss at 12 months.

# Abstract: Survival Rate of Implants Placed with Sinus Lift, Impact of Schneiderian Membrane Perforation: Systematic Review and Meta-Analysis

**Authors:** M. Arguimbau Coll, V. Schiavo Di Flaviano, S. Egido Moreno, B. González Navarro, E. Velasco Ortega, J. López-López, L. Monsalve Guil

**Affiliations:** *Master in Oral Medicine, Surgery and Implantology – Faculty of Medicine and Health Sciences – University of Barcelona*

## Objectives

 The maxillary sinus lift procedure using the lateral window technique for the rehabilitation of the upper posterior region is considered highly predictable. However, the perforation of the Schneiderian membrane is the most common intraoperative complication, occurring in 10-60% of cases. This study aimed to evaluate whether the survival rate of dental implants is affected by the perforation and repair of the sinus membrane during surgery, compared to cases where no perforation occurs.

## Material and Methods

 In May 2024, a systematic review was conducted using the electronic databases Medline/PubMed, Scopus, and Web of Science, employing the MeSH term "Sinus floor Augmentation." Additionally, a meta-analysis was performed to assess the survival rate of implants placed using the sinus lift technique with a lateral window approach, in cases where the Schneiderian membrane was perforated. The odds ratio was calculated to compare implants placed in perforated versus non-perforated sinuses.

## Results

 A total of 283 articles were reviewed, from which 10 were selected for systematic review and 7 for meta-analysis. The average follow-up of the patients was 25.15 months. In total, 1666 patients received 2229 sinus lifts and 5052 implants. The average incidence of membrane perforation was 29.42%. Postoperative complications included sinusitis, infection of the surgical wound, rhinorrhea, and graft necrosis, although not all articles specified these complications. In the studies included in the meta-analysis, 1224 sinus lifts were performed without membrane perforation, placing 2725 implants, with 62 failures (97.7% survival rate). In 480 lifts with perforation, 1044 implants were placed, with 30 failures (97.1% survival rate).

## Conclusions

 The difference in implant survival rates between perforated and non-perforated sinuses was not statistically significant. Intraoperative repair of the perforation did not negatively affect implant survival. Therefore, membrane perforation should not be a reason to abort the procedure or an absolute contraindication for implant placement.

# Abstract: Survival of dental implants in patients with chronic renal failure - Systematic review

**Authors:** E. Carrasco Núñez, D. Sánchez Gimeno, E. Mauri Obradors, M. Sabater Recolons, J. López-López

**Affiliations:** *University of Barcelona UB, Department of Odontostomatology*

## Objectives

 Identify the survival of dental implants in patients with chronic kidney disease (CKD) based on the current literature.

## Material and Methods

 A literature search was carried out in PUBMED database. In the screening we obtained 64 articles, and finally obtained 6 by applying the exclusion and inclusion criteria. The articles chosen included systematic reviews and prospective case-control and long-term studies.

## Results

 Chronic kidney disease (CKD) is a public health problem associated with serious complications with an overall prevalence of 8% to 16%. It is based on markers of renal damage or the measurement of a glomerular filtration rate of less than 60 ml min per 1.73 m2 for at least 3 months. One of its complications is systemic dysfunction of bone and mineral metabolism, which may involve growth abnormalities, bone turnover and vascular or soft tissue calcifications. The results obtained in this study showed high implant survival success rates in patients with CKD relative to healthy patients. Implant survival rates of 98% after 10 years of follow-up and 90.9% after 15 years of follow-up were found.

## Conclusions

 CKD represents severe complications and comorbidities that can affect bone quality and the healing process. The placement of dental implants is not a contraindication in this type of patient, but due to the high percentage of mucositis and peri-implantitis found, periodic medical monitoring is recommended, as well as maintenance of periodontal health status in order to ensure high survival rates.

# Abstract: Management of the anticoagulated patient in implant surgery, an updated bibliographic review

**Authors:** G. Vaca Quirola, S. Cacciacane, A. Boquete Castro

**Affiliations:** *UEMC de Máster de Formación Permanente en Implantología y Rehabilitación Oral, Barcelona*

## Introduction

 The rise in life expectancy leads to the frequent treatment of elderly patients who are on long-term anticoagulant medication due to their medical conditions; this situation raises questions about whether or not to request a modification of their medication.

## Objectives

 To conduct an updated literature review on the approach to implantologic surgery in anticoagulated patients in order to determine whether there is a standard strategy regarding the continuation or withdrawal of anticoagulant medication. 

## Material and Methods

 A literature search was conducted in the PubMed database using the following search strategy: "Dental Implants"[Majr]) AND "Anticoagulants"[Mesh]; bleeding risk; DOAC. The filters applied were: Clinical Trial, Meta-Analysis Randomized Controlled Trial, from the last 10 years, in English. A total of 65 articles were obtained; with a more specific search strategy, 23 articles were retrieved. After reading the abstracts, 11 open-access articles were reviewed, excluding those that did not refer to implant surgery. Finally, 5 articles were included in the review: 3 Systematic reviews, 1 RCT, and 1 retrospective study.

## Results

 For anticoagulant therapy with Warfarin and acenocoumarol, neither withdrawal nor dose modification of the anticoagulant is required, but it is necessary to establish the INR 24 hours before the procedure. Regarding DOACs (rivaroxaban, dabigatran, apixaban, edoxaban), studies indicate that medication should not be withdrawn for low-risk surgery, but the surgery should be scheduled away from the last dose; in cases of high-risk surgery, it is suggested to delay or skip the last dose. All articles recommend post-surgical hemostatic measures.

## Conclusions

 There is a lack of studies on anticoagulant effects in implant surgery, so no definitive guidelines exist for DOAC cessation. The bleeding risk in implant surgery can be managed with hemostatic measures; however, we cannot control the risk of thrombotic events that can be fatal if the medication iswithdrawn.

# Abstract: Impact of Platform Switching on Dental Implants

**Authors:** D.F. Gallardo Loayza, J.M. Acuña Pedrosa, A. Boquete Castro

**Affiliations:** *Máster en formación permanente en cirugía oral, implantología y periodoncia. JMAP Sevilla UEMC*

## Objectives

 To evaluate the impact of platform switching on dental implants.

## Material and Methods

 A search was conducted in the MEDLINE-PubMed databases and a literature review was performed on seven articles from 2014 to 2024 that assessed crestal bone loss in implants with platform switching. To be included, the studies had to specifically mention platform switching.

## Results

 Some studies, such as Linkevicius's, suggest that platform switching alone might not be sufficient, particularly in cases of reduced mucosal tissue thickness. The variability in bone loss across studies highlights the complexity of the topic and the influence of multiple biological and biomechanical factors. It was observed that prostheses with platform switching showed less bone resorption around the implants compared to compatible platform prostheses. Statistical analyses demonstrated significant differences in bone resorption under different conditions, such as implant placement, implant-abutment diameter, and follow-up period. Characteristics like implant length and diameter, and type of prosthesis (screw-retained or cemented) did not have a significant effect on bone resorption. The use of platform switching components appears to be an effective strategy to reduce marginal peri-implant bone loss, enhancing implant stability and contributing to the longevity of implant treatment. Combining platform switching with a conical connection seems to be a promising strategy to promote osseointegration and implant stability.

## Conclusions

 Platform switching does not prevent crestal bone loss in thin mucosal tissues but does minimize bone loss in thicker tissues. Further long-term research is needed to confirm the results of this study over a longer observation period.

# Abstract: Comparison between low speed drilling without irrigation or biological and conventional drilling. Bibliographic review

**Authors:** E. Aramburu Carudel

**Affiliations:** *Master de cirugía, periodoncia e implantología. Universidad de León*

## Objectives

 The general objective of this article is to compare low-speed non-irrigated or biological drilling with conventional drilling for the preparation of dental implant osteotomies.

## Material and Methods

 To develop this bibliographic review, searches were carried out in three databases (Pubmed, Mendeley and Google Scholar). The variables were the duration of the study, the temperature reached during drilling, the drilling technique, the analysis method used, the quantity and quality of bone extracted, bone loss, and survival or success of the implant. The following inclusion criteria were followed: articles less than 10 years old since their publication, with the exception of the article by Anitua et al. 2007, articles reported in English and clinical studies and in vitro studies conducted in humans.

## Results and Discussion

: A comparative analysis was performed based on all existing data in the 10 studies included in this review. Information on a total of 415 implants. All studies are divided into two groups according to the drilling method used; low speed milling without irrigation and conventional milling. Four of the studies evaluate the success and survival of the implants, finding some controversy in the results. Regarding bone loss, there were no significant differences in any of the studies. Others compared the quality and quantity of bone extracted during osteotomy, finding greater osteogenic efficacy in bone extracted by low-speed drilling. In terms of patient comfort, low-speed drilling showed less postoperative pain and inflammation.

## Conclusions

 We can show that low speed drilling is more effective for obtaining autologous bone. This technique is comparable to the conventional drilling technique for implants. However, more clinical studies with a larger number of samples are needed.

# Abstract: Socket shield bibliographic review

**Authors:** J.G. Salazar Sanchez

**Affiliations:** *ADESLAS DENTAL and ASISA DENTAL BADAJOZ*

## Objectives

 The key objective of this bibliographic review is to analyze this new technique, in order to see the indications, contraindications, advantages, disadvantages and complications of the technique.

## Material and Methods

 A bibliographic search has been carried out in the Pubmed database, tracing the last 10 years. The keywords used were: "Dental implants", "Socket shield technique", "Alveolar ridge preservation", "Root submergence technique".

## Results

 The socket-shield technique, based on the root immersion technique (RST), in which a partial buccal root fragment was retained around the root simultaneous with the immediate implant placement. The desired effect was to maintain the healthy periodontium, thereby maintaining the gingival tissues and maintaining the hidral bone at its original level. The roots were sectioned in a mesial-distal direction using a long shank carbide on a high speed turbine, In order to obtain a facial and lingual segment, following which, the lingual fragment was carefully extracted using periotomes and luxators. The remaining root fragment was now reduced apico-coronally to about 2- 2.5mm below the free gingival margin and thinned bucco-lingually to about 1.5mm thickness. An osteotomy was then sequentially prepared and implants wend inserted palatal to the Socket shield via a prosthodontically planned surgical guide with the implant table 2 mm below the facial crest. Any jump gap of >=4mm was grafted with CPS putty.

## Conclusions

 The socket-shield technique has demonstrated histological and clinical results that contribute to the improvement of aesthetics in implant treatment. At the same time, this technique preserves the buccal cortical plate and healthy peri-implant tissues can be observed, proving to be one of the most conservative ridge preservation techniques.

# Abstract: Rehabilitation using custom 3d subperiosteal structures as a reconstructive option for atrophic jaws

**Authors:** I. Eguzkiagirre Martínez, L. Horcajo De Cabo, A. Garcia Hernandez, C.B. Jacobo Orea

**Affiliations:** *Master in Oral Surgery, Implantology and Periodontology at the University of León*

## Introduction

 The rehabilitation of atrophic jaws is complex due to significant bone loss, which affects chewing, speech, and aesthetics. Although endosseous dental implants are effective through osseointegration, in cases of severe atrophy, the available bone quantity is insufficient, necessitating complicated and costly procedures. Subperiosteal implants, introduced by Dahl in 1938, have resurfaced thanks to technologies such as 3D printing and digital scanning, allowing for custom designs that improve clinical outcomes and reduce complications, although they are not without risks.

## Objectives

 To analyze the design of subperiosteal implants using new 3D technologies and their complications.

## Material and Methods

 A search was conducted in databases such as PubMed, Medline, and Scielo, reviewing articles from the past decade. Studies that were not relevant or not in English or Spanish and focused on jaws were excluded.

## Results

 The reviewed studies, mostly retrospective case series, report complications such as pain, swelling, and edema, generally resolved within a few days, with a higher incidence in soft tissues. In a follow-up of 8 months to 3 years on 215 implants, 10 failed, indicating a success rate of 95.43%.

## Conclusions

 Subperiosteal implants fabricated using 3D structures face certain challenges; however, they provide good long-term outcomes with a high success rate and good survival in most cases. Nonetheless, studies evaluating long-term clinical behavior are needed.

# Abstract: Biomaterials for the prevention of dental bone resorption: efficacy and limitations

**Authors:** R. González Chiriboga, S. Cacciacane, A. Boquete Castro

**Affiliations:** *Universidad Europea Miguel de Cervantes (UEMC)*

## Introduction

 The aim of this study was to describe different techniques to help prevent alveolar ridge resorption, evaluating the changes produced after tooth extraction compared to extraction alone or among various materials.

## Material and Methods

 The following electronic databases were used: MEDLINE/PubMed, Cochrane Oral Health's Trials Register, and Cochrane Central Register of Controlled Trials (CENTRAL). The current bibliographic search included controlled and randomized trials, systematic reviews, and meta-analyses. Restrictions were applied regarding language, including only articles in English, studied groups (human or animal), and date of publication. Finally, eleven journal articles with a relevant impact factor were selected to compare different materials.

## Results

 Significant results were found when observing horizontal changes (in mm) in the 11 articles included. However, vertical changes were not significant. The studies that compared more than one alveolar ridge preservation technique were not statistically relevant. The use of membranes with alveolar ridge preservation techniques obtained better results. The most studied materials were grafts of non-bone origin, followed by xenografts.

## Conclusions

 The use of biomaterials after extraction helps minimize resorption of the alveolar ridge and reduces the need for additional grafts or bone expansion procedures for implant placement. The combined use of biomaterials with resorbable membranes significantly improves outcomes. Xenografts and allografts have been the most studied materials in this review. Autologous bone grafts are the most predictable option in terms of results and effectiveness. Despite the use of biomaterials, some loss of both vertical and horizontal alveolar ridge dimensions is expected, with the latter being more significant.

# Abstract: Melatonin and growth hormone in the reduction of osseointegration times of dental implants

**Authors:** O.C. Mancusi Rivas

**Affiliations:** *Centro odontológico de formación integral de León*

## Introduction

 In the field of implantology, the phenomena of both osseointegration and primary stability are fundamental, which depend on the bone and the remodeling phenomenon. The ultimate goal of remodeling is to eliminate old bone to create new bone and this is regulated not only by growth factors but also by hormones such as growth hormone and melatonin

## Objectives

 Detail the current evidence of the benefits of the application of melatonin and growth hormone in reducing osseointegration times of dental implants, focusing on the ISQ and the BIC.

## Material and Methods

 A systematic search was carried out in the PubMed, Cochrane and Google Scholar databases. The strategy included keywords "Melatonin and dental implant", "melatonin and oral implantology", "growth hormone and dental implant" and "growth hormone and oral implantology" applying as a filter articles published from 2012, and written in English or Spanish.

## Results

 A total of 9 articles were selected, all of them being case-control studies. Of the included articles, 2 were about growth hormone, 6 about melatonin, and 1 about both. Furthermore, 7 of the included articles assess the BIC and 4 the ISQ.

## Conclusions

 The application of melatonin and growth hormone when placing dental implants turned out to favor osseointegration, bone formation and their stability, thereby improving and speeding up our treatments, being a promising option to take into account in its application in clinical, although more studies are required to corroborate these results.

# Abstract: Management of soft tissues in the prevention of aesthetic complications in implantology

**Authors:** V. Hoyas Fernánde, J.M. Acuña Pedrosa, A. Boquete Castro

**Affiliations:** *JMAP Formación SEVILLA . Universidad Europea Miguel de Cervantes*

## Introduction

 The presence of soft tissues is key for the prevention and reduction of esthetic and biological complications in implant dentistry, such as inflammation, recession and disease. The aim of this work is to analyze the relationship between soft tissue treatment, esthetics and prevention of complications in implant dentistry.

## Material and Methods

 A literature review was performed in Pubmed using the keywords: Esthetic, Soft tissue, implantology, in a combined manner. The following inclusion criteria were introduced: articles published in the last 10 years, systematic reviews, both in English and Spanish and performed in humans, and all those that did not meet the established criteria were excluded.

## Results

 The scientific literature has evaluated soft tissue augmentation procedures to increase the width of keratinized tissue in peri-implant health or disease. Reductions in gingival and plaque indices are found after mucosal augmentation procedures. However, there is no difference in bleeding on probing, less marginal bone loss with the use of connective tissue grafting in the esthetic zone.Other authors have evaluated how increasing the soft tissues reports positive data at the esthetic level, stable tissues and prevention of recession.

## Conclusions

 Based on the hypothesis proposed in the present work, the relationship between soft tissue management and esthetics in the results of the implants has become evident, and the following conclusions have been reached: The regeneration of soft tissues during implant treatment is important to obtain stable peri-implant tissues, as well as to prevent the appearance of recession and inflammation of the implant.

# Abstract: Implant Management in Patients Treated with Radiotherapy. A Literature Review

**Authors:** E. Pardo, P. Egido, A.I. Gordo, R. Mateos, M.A. Alobera, M. del Canto

**Affiliations:** *Máster en Cirugía Bucal, Implantología y Periodoncia de la Universidad de León*

## Introduction

 Nowadays radiotherapy is a frequent treatment, either primary or combined, for patients with head and neck cancer. This treatment can cause side effects on oral health that can influence implants already in place or their subsequent placement.

## Objectives

 To analyze the survival rate and success of dental implants in irradiated patients with various doses and types compared to those not treated, and to identify the variable factors to achieve greater success.

## Material and Methods

 A literature search was performed in PubMed database using the keywords "dental implant", "radiotherapy" y "osteoradionecrosis".

## Results

 Statistically significant differences are observed in the implant failure rate according to the total radiation dose, as well as the frequency of its fractionation, without distinguishing between one type or another. There are no significant differences regarding the occurrence of osteoradionecrosis between the maxilla and the mandible; however, when additional factors (tobacco, alcohol, diabetes, and osteoporosis) are included, there is a higher likelihood of developing this complication. The waiting time for implant placement after radiation is crucial, recommending a minimum of 6 months to 2 years or more to significantly increase the success rate.

## Conclusions

 Despite the high survival and success rate of dental implants in patients treated with radiotherapy, it is crucial to consider the possibility of complications. Further long-term studies are required to evaluate the impact of radiotherapy on oral health and how to improve the success rate, thus achieving a better quality of life after cancer.

# Abstract: Artificial intelligence and its application in implantology

**Authors:** M.C. Ocaña Ramos, J. Acuña Pedrosa, A. Boquete Castro

**Affiliations:** *Master's degree in continuing education in Oral Surgery, Implantology, and Periodontics. European University Miguel de Cervantes. JMAP FORMATION*

## Objectives

 Artificial intelligence (AI) has revolutionized dentistry, offering tools that improve the precision and efficiency in the planning and placement of implants, as well as reducing postoperative complications. The goal is to explore the applications of AI in implant planning and delve into the use of AI as a tool for implant surgery through medical robotics and robotic assistants.

## Material and Methods

 Bibliographic searches in databases: Pubmed Central, Scopus, and Science Direct, using the keywords: "artificial intelligence" AND "oral surgery", "artificial intelligence" AND "dental implants". The inclusion criteria were articles in English language, clinical studies in humans, in vitro studies, articles that include diagnosis through AI in dental surgery and dentistry in the last 10 years. The exclusion criteria are those articles that do not apply AI as material or method.

## Results

 A higher precision in the location and planning of implants using AI in simulations is determined compared to traditional systems. Highlighting the interventions carried out by robotic assistants that reduce surgical trauma, shorten work times, patient recovery, and operator fatigue. Not showing statistically significant results. Resulting from variants such as: limited clinical studies, little evidence of long-term outcomes, the need to create standardized guidelines or protocols, and the refinement of robotic technology.

## Conclusions

 AI is an innovative tool in dental implant surgery. Its application in planning, design, robotic-assisted placement, and complication detection improves the accuracy and efficiency of procedures, providing better results for patients. Predicting a promising future in the field of oral implantology and the use of surgical robotics. Further research and development are needed in this field to advance the integration of AI in dentistry.

# Abstract: Guided surgery in oral implantology. Updated bibliographic review

**Authors:** L. Benítez Ariza, J.M. Acuña Pedrosa, A. Boquete Castro

**Affiliations:** *Master of continuing education in oral surgery, implantology and periodontology. September 2023/November 2024. JAMP formacion acca learning. Seville*

## Objectives

 The aim of this study is to review and update the latest knowledge in the field of guided surgery and its application in oral implantology.

## Material and Methods

 A literature search was carried out in the following databases: Medline, PubMed, Scielo, and articles published in English and Spanish in the last 5 years and applied to humans were selected as inclusion criteria, using the keywords "guided surgery", "immediate loading", "surgical splints", "CBTC".

## Results

 Digital imaging allows dentists to operate with surgical splints and perform precise immediate loading that will increase patient comfort compared to conventional implant surgery. Several studies have shown that guided surgery has a mean coronal error of 0.99mm and 1.24mm at the apex and a mean angular deviation of 3.81º, so there are significant differences in favour of the parameters of guided surgery versus conventional surgery. Guided surgery offers numerous benefits as it allows for exact precision in the placement and parallelisation of implants, which facilitates correct insertion of the prosthesis and reduces surgical time. It is a minimally invasive surgery in which in many cases it is not necessary to make a flap and significantly reduces inflammation, discomfort and bleeding. It also allows us to avoid invasion of important anatomical structures such as the maxillary sinus, the dental nerve or any blood plexus.

## Conclusions

 Guided surgery allows an exhaustive planning when placing implants in such a way that both the surgical phase and the future prosthodontic phase can be coordinated in an exquisite way, which provides the opportunity to obtain great functional and aesthetic results.

# Abstract: Behaviour of Dental Implants in Oncology Patients

**Authors:** L.E. Guilbeaux Urgelles, S. Cacciacane, A. Boquete

**Affiliations:** *Centro Medico Dental Guayaquil . Calle Valencia 632 Barcelona, España*

## Objectives

 The objective of this bibliografic review is to analizethe effects of radiotherapy and chemotherapy on oral tissues, the complications that may appear in treatmentwith dental implants ,as well as the recommendations and protocols to follow to try to avoid them.

## Material and Methods

 Bibliographic search with the keys: " dental ", " implants ", " radiotherapy ", " radiation ", "biphosphonates ", and " osteonecrosis ". Inclusion criteria: By the year of the articles

## Results and Discussion

: 60%-80% of patients with head and neck cancer are treated with radiotherapy. The degree of involvement will depend on the dose, the radiation field and the treatment scheme. Complications include: Xerostomia, mucositis, fibrosis, demineralizacion, caries, sensory dysfunction, increased risk of infection, and avascular bone necrosis ( osteoradionecrosis). Other complications such as pain, swelling, infection, radiographic changes. ONR occurs spontaneously or in response to an injury with doses greater than 60 Gy, more frecuent in the mandible and anterior maxillary region. Treatment for these patients would be: Antibiotic prophylaxis (amoxicillin 500 mg for 14 days starting one day before the intervention), surgical field as aseptic as posible, wait 9-12 months from the last doses less than 50 Gy, do not perform inmediate or early loading , implant-supported prostheses. Use hyperbaric oxygen therapy as an adjunct. The effects of BMPs and OGP as adjuvants are being studied.

## Conclusions

 Radiotherapy and Chemotherapyhave negative effects on oral tissues, the main complication being osteoradionecrosis and osteonecrosis, which can cause treatment failure with dental implants. Therefore, it is necesary a series of protocols and recommendations to avoid these complications.

# Abstract: Decontamination of implants with periimplantitis

**Authors:** S. de Dios, S. Cacciacane, A. Boquete, S. Udina, Y. Garcia

**Affiliations:** *Máster de implantología y rehabilitación oral - Universidad Europea Miguel de Cervantes y ESIRO*

## Objectives

 To review the indexed literature regarding the various therapeutic interventions proposed for the decontamination of implants with peri-implantitis.

## Material and Methods

 This study was conducted by performing a bibliographic review based on information obtained from six studies found in the following databases: PubMed, Cochrane, World Health Organization, and Scielo. The aim was to synthesize the available scientific information to enable professionals to acquire and update their knowledge regarding peri-implantitis treatments. The inclusion criteria were: articles published in the last ten years, studies conducted on humans of any age group, articles in English or Spanish, and all types of Materials and Methods.

## Results

 Studies on peri-implantitis report various protocols for mechanical, chemical, and physical decontamination. Treatments are divided into non-surgical (mechanical, antiseptic, and antibiotic), surface decontamination (chemical, such as citric acid and chlorhexidine, and laser), and surgical (abrasive powder, resective, and regenerative). These methods, alone or combined, improve clinical parameters. The protocol of mechanical decontamination, chemical detoxification, and bone regeneration is effective and reproducible, showing improvements and stability in clinical parameters and radiographic bone levels over one year. Systemic antibiotics, Er:YAG and CO2 lasers, mechanical debridement with sodium bicarbonate, and surgical treatment offer short-term clinical benefits.

## Conclusions

 No method demonstrated clear superiority. Treatment selection should be based on the extent of the disease, with non-surgical mechanical therapy recommended as the initial approach. There is no consensus on the best treatment protocol for peri-implant diseases. Non-surgical mechanical therapy is beneficial for peri-implantitis and should be the initial treatment. Well-designed studies are needed to identify the most effective decontamination method and to evaluate complementary measures.

# Abstract: Exploring the Similarity of Submucosal and Subgingival Plaque Microbiomes in Peri-Implant and Periodontal Conditions

**Authors:** A. Sánchez Barco, A. Regueira Iglesias, B. Suárez Rodríguez, T. Blanco Pintos, N. Seijas Otero, I. Tomás

**Affiliations:** *Oral Sciences Research Group, Special Needs Unit, Department of Surgery and Medical-Surgical Specialties, School of Medicine and Dentistry, Universidade de Santiago de Compostela, Instituto de Investigación Sanitaria de Santiago (IDIS), Santiago de Compostela, Spain*

## Objectives

 To compare the submucosal plaque microbiome in peri-implant health and peri-implantitis with the subgingival plaque microbiome in periodontal health and periodontitis at the amplicon sequence variant (ASV) level. This was done regarding alpha-diversity, community structure, and differential abundance under a compositional data analysis approach.

## Material and Methods

 Searches were performed in several databases to identify Illumina V3-V4 investigations on the subgingival/submucosal microbiome in periodontal health (Sub_x0HHx), periodontitis (Sub_x0PDx), peri-implant health (Imp_x0IHx), and peri-implantitis (Imp_xIDx). Studies that met predefined criteria were included in our analysis (Sub_x0HHx=45; Sub_x0PDx=45; Imp_x0IHx=36; Imp_xIDx=41). Sequences were processed under the mothur pipeline for ASVs, and an oral-specific database was used for taxonomic assignment. Statistical analyses were conducted in the R environment.

## Results

 More ASVs were observed in Imp_x0IHx than Imp_xIDx (421.00 vs. 288.00; p<0.01). "Sub" plaque of healthy implants had lower 95% coverage than healthy teeth (97.50 vs. 178.00; p<0.05) and less diversity and evenness than diseased teeth (Shannon=3.44 vs. 4.14; Pielou=0.55 vs. 0.68; p<0.01). In peri-implantitis, alpha-diversity was lower than in perio-health (ASVs=288.00 vs. 461.00; Coverage=83.00 vs. 178.00; Shannon=3.59 vs. 3.87; p<0.01) and periodontitis (Coverage=132.00; Shannon=4.14; Pielou=0.62 vs. 0.68; p<0.01). In the PCA, "sub" samples were clustered according to the group to which they belonged (p<0.01). Finally, the highest number of ASVs with differential abundance between groups was obtained with Imp_x0IDx vs. Sub_x0HHx (139; 1.31%). This was followed by: Imp_x0IHx vs. Imp_x0IDx (87; 0.82%), Sub_x0PDx (86; 0.81%), Sub_x0HHx (76; 0.72%), and Imp_x0IDx vs. Sub_x0PDx (29; 0.27%).

## Conclusions

 The healthy submucosal plaque is richer than the diseased. Overall, submucosal plaque is less alpha-diverse than subgingival in health and disease. The structure of the microbial communities is different for all study groups. Regarding differential abundance, the plaque microbiome was more distinct between opposing health conditions than among submucosal and subgingival niches with the same condition.

# Abstract: The role of oral fluid biomarkers in diagnosing peri-implantitis

**Authors:** A. Hermo Carmona, S. Cacciacane Entreríos, A. Boquete Castro

**Affiliations:** *Escuela Superior de Implantologia (ESIRO), Universidad Europea Miguel de Cervantes*

## Objectives

 Despite the prevalence, diagnosing peri-implant disease remains challenging as common diagnostic methods of periodontal probing and radiographs may be inaccurate. These methods only document pre-existing destruction rather than current disease activity. Biomarkers in peri-implant crevicular fluid show promising results in regard to their diagnostic and prognostic value. The aim of this review is to analyze biomarkers in gingival crevicular fluid for the diagnosis of peri-implant pockets.

## Material and Methods

 The search for articles was conducted in two electronic databases, Medline (PubMed) and Cochrane. Eligible articles were those that evaluated any biomarker in gingival crevicular fluid in individuals clinically diagnosed with peri-implant disease. The methodological quality was assessed using the Newcastle- Ottawa Scale (NOS) for cohort studies and the modified NOS for cross-sectional studies.

## Results

 17 studies that met the inclusion criteria were included, with sample sizes ranging from 10 to 60 subjects. Among these papers, 49 biomarkers were identified for the detection of peri-implant disease. Our primary focus lies on IL-1β, IL-6, IL-10, TNF-a, and MMP-8, which are extensively investigated cytokines and showed significantly increased in both salivary and peri- implant sulcular fluid samples. Additionally, alterations in bone loss markers have shown potential as indicators of disease progression and treatment response.

## Conclusions

 Several proinflammatory cytokines have been identified as promising biomarkers of gingival crevicular fluid for the diagnosis and monitoring peri-implant disease. IL-1β, IL-6, and MMP-8 are some of the most reliable biomarkers that can be used as complementary tools to clinical parameters. Future studies should focus on establishing standardized protocols and conducting well-designed clinical trials to validate the diagnostic accuracy and clinical relevance of these biomarkers.

# Abstract: Simultaneous Bone Regeneration with Implant Placement in Areas with Minor Bone Defects

**Authors:** C. Calizaya Rodriguez, A. Boquete Castro, S. Cacciacane

**Affiliations:** *Master formacion permanente en implantologia y rehabilitacion oral internacional*

## Introduction

 After tooth extraction, there is a progressive loss of alveolar bone, mainly affecting the buccal bone wall. Techniques that preserve the alveolar ridge can lead to successful outcomes by maintaining the ideal width and height necessary for implant placement. Bone defects such as fenestrations or dehiscences, which compromise the primary stability and integration of implants, can be addressed through Guided Bone Regeneration (GBR).

## Objectives

 To review the scientific literature to determine if minor peri-implant defects affect the primary stability of implants and to analyse which regeneration procedures have demonstrated the best results in correcting bone defects.

## Results

 For vertical defects, simultaneous GBR is feasible if the defect is ≤4.1 mm, while prior GBR is performed when the defect is 4.7 mm. A 2 mm bone wall is required to ensure adequate support of soft tissues and to prevent complete resorption of the buccal bone wall. In small fenestrations and dehiscences forming cavities around the implant, a resorbable barrier combined with particulate bone is preferred, likely representing the treatment of choice.

## Conclusions

 Vertical peri-implant defects ≤4.1 mm have shown no effect on the primary stability of the implant. If the minimum of 2 mm around the implant is lacking during placement, simultaneous GBR can be performed to generate sufficient bone volume to support the implant and mucosa. The greatest reduction in defects was achieved when a barrier membrane was combined with bone graft material.

# Abstract: Prevalence of benign tumors of the oral mucosa

**Authors:** O. Navas Coscojuela, S. Cacciacane, A. Boquete

**Affiliations:** *Master Internacional implantología y Rehabilitación Oral, ESIRO Barcelona*

## Objectives

 To know what the prevalence of benign tumors of the oral mucosa is. To know what their etiology is and what factors are directly associated; study what are its clinical characteristics and most frequent location; and determine how their diagnosis and treatment is implemented.

## Material and Methods

 A bibliographic search was performed in the main databases Pubmed, Dentistry and Oral Sciences Source (DOSS) and Google Scholar (GA). Inclusion criteria: free full text, books and documents, clinical trial, meta-analysis, randomized controlled trial, review, systematic review, in humans. Languages of the articles: english, spanish or french. Exclusion criteria: articles over 5 years old and articles with restricted access. A total of 33 articles were obtained in Pubmed, 69 in GA and 45 in DOSS; finally, a total of 27 articles were selected.

## Results

 The prevalence of benign tumors of the oral mucosa ranges between 6.9% and 12.5%. The most prevalent one is the fibroma with a certain predilection for the female sex, and a higher incidence between the 3rd and 5th decades of life.

## Conclusions

 The etiology of these tumors is unknown for the majority. In addition to their common clinical characteristics, each one presents certain individualities. Their most frequent locations are: buccal mucosa, palate, tongue, lips and gingiva. The diagnosis is fundamentally based on the clinical appearance and histology. Treatment is commonly surgical excision.

# Abstract: Correlation between periodontal disease and COVID 19

**Authors:** J. Gutiérrez Rubio, M.J. Gutiérrez González, I. Goiti Loizaga

**Affiliations:** *Centro Odontológico de Formación Integral de León*

## Objectives

 The literature review carried out attempts to understand the current status of the various studies carried out that postulate a hypothetical relationship between periodontal disease and COVID 19 and to clarify as far as possible the existence or not of a consensus between their results.

## Material and Methods

 The search for articles was carried out on the PUBMED platform. The keywords used were: COVID 19, Periodontitis, peri-implantitis and association.

## Results

 Periodontal disease is one of the most prevalent in the world population, affecting millions of patients to a greater or lesser degree. It is produced by an alteration in the oral microbiota that produces an inflammatory response that causes the destruction of the supporting tissues of the teeth. At the same time, the appearance of the COVID 19 disease caused by the SARS-CoV-2 virus has represented the greatest health emergency in history, causing a planetary pandemic. This virus presents symptoms similar to those of the flu, but sometimes serious complications appear, such as pneumonia, which can lead to the death of the infected person. Scientific evidence indicates that the most severe cases are affected by the activation of an inflammatory cascade mediated by angiotensin-converting enzyme 2 (ACE2).

## Conclusions

 As the two diseases are individually related to inflammatory processes, the study of the current scientific literature suggests that possibly advanced periodontal disease is a risk factor for the individual affected by COVID 19 to suffer serious complications. But methodological limitations suggest that the results should be taken with caution, hoping that future studies with standardized methodology will help clarify the possible relationship between the two diseases.

# Abstract: Short dental implants: an innovative option for oral rehabilitation

**Authors:** A. Tapia Aranda

**Affiliations:** *Máster Propio de Formación Permanente en Cirugía Bucal, Implantología y Periodoncia. Promoción 2023/2025 JMAP Formación*

## Objectives

 To evaluate the effectiveness and advances in the use of short dental implants (less than 8 mm in length), where short dental implants are the best option in the absence of adequate bone height for a conventional implant, less invasive for the patient, with effective short-term and fully functional results. For this purpose, an updated review of the literature was carried out, reflecting the use of short implants, their advances in improving materials and designs, their use techniques and their effectiveness.

## Material and Methods

 This study was based on scientific research articles published from 2015 to the present, journals such as implant Dialogue Edition 1 year 2015, Extra-short implants (≤6.5 mm in length) in atrophic and non-atrophic sites to support screw -retained full-arch restoration: a retrospective clinical study. PubMed, Scielo. In which articles were selected by the impact index of the journals. Keywords: short dental implants, atrophic jaws, alternatives to conventional implants. Inclusion Criteria: Clinical studies and systematic reviews on short dental implants from 2014 to 2024. Analyzed Data: Success rates, implant survival, complications, alternative such as less invasive surgery and technological advances.

## Results

 Scientific evidence reports success rates comparable to longer/conventional implants, this supports the use of short implants, with a success rate of 95% in 5 years. Regarding the rough treated surfaces with titanium, improvements in the geometry, anatomy of the implant, the use of bioactive surfaces, digital planning and guided surgery have allowed us to increase the success rates and the predictability and confidence of the implants. shorts. It is a proven alternative for classic rehabilitation with bone grafts and the risks that it entails, such as resorption by the graft used, mainly due to the poor vascularization of the recipient bed, common in these patient cases.

## Conclusions

 Short dental implants are an effective and reliable alternative for patients with limited bone height. Technological evolution and advanced surgical techniques have significantly improved the success and survival rates of these implants, therefore, it is an excellent alternative for rehabilitating jaws where there is not enough height to correctly submerge a standard length implant, where it will be reflected a reduction in treatment time and morbidity, which translates into a high patient acceptance rate. However, longer-term studies are necessary to confirm durability.

# Abstract: Penicillin allergy as a risk factor for dental implants: A narrative literature review

**Authors:** S. Udina Silvestre, A. Castro Boquete, S. Cacciacane, S. De Dios, M. Arabi Tihilili

**Affiliations:** *Universidad Europea Miguel de Cervantes*

## Introduction

 Individuals allergic to penicillin constitute between 10% and 20% of the total population, representing a significant proportion of the patients we treat in dental practice. Currently, the placement of dental implants is one of the most commonly performed treatments, with very high success and survival rates. However, in this group of individuals, there is a higher risk of failure. The causes of this increased risk are not fully defined, but It seems to have a relationship involving genetic factors, such as the HLA gene polymorphism, and the use of alternative antibiotics when treating these individuals.

## Objectives

 This narrative literature review aims to analyze systematic reviews on dental implant failure in individuals allergic to penicillin. The objective is to understand the causes, statistics, and parameters that have been studied.

## Material and Methods

 A search was conducted in the PubMed and Cochrane databases using the following keywords: "penicillin allergy" and "dental implants". The inclusion criteria when selecting the papers were: "systematic review" and "penicillin allergy as a study parameter". Ultimately, only 4 of the 12 articles were included in the review.

## Results and Conclusions

 Penicillin allergy alone for itself does not seem to be a risk factor for dental implant failure. However, the administration of clindamycin is associated with a higher rate of implant failure compared to the use of amoxicillin. Therefore, the use of other antibiotics such as azithromycin, metronidazole, or clarithromycin is recommended.

# Abstract: Functionalisation of sutures with nanomaterials in oral surgery: a review

**Authors:** A. Artieda Estanga, A. Quintanilla Cortés, L. Pastor, C. Barrilero Martín, M.A. Alobera Gracia

**Affiliations:** *Máster en cirugía bucal, implantología y periodoncia. Universidad de León*

## Objectives

 The aim of this review was to investigate the development of functionalised antimicrobial sutures in the past decade to avoid SSIs.

## Material and Methods

 A review was conducted (Pubmed) from January 2014 up to May 2024. In vivo and in vitro studies evaluating the development of antimicrobial sutures were eligible. The related key words included (suture AND oral surgery), (suture AND nanomaterial) and (suture AND bacterial infection). Non-English language literature was excluded.

## Results

 From 2592 titles identified by the search strategy, 52 were included. These studies show that in the past decade a variety of novel antimicrobial sutures have been developed with new formulations that are able to minimise the risk of SSIs maintaining or improving their mechanical properties.

## Conclusions

 Among the new formulations, the use of metallic nanoparticles stands out. The newly designed sutures may be good alternatives to conventional sutures. More clinical trials are needed to ensure that the discoveries are going to be relevant on patients undergoing oral surgery.

# Abstract: Comparison of the physical and chemical properties of filling materials in the perioral area

**Authors:** L. García, S. Cacciacane, A. Boquete

**Affiliations:** *Máster internacional de implantología y rehabilitación oral, ESIRO Barcelona*

## Objectives

 To carry out an updated literature review of the temporary filler materials currently approved in Spain and compare them according to their physicochemical and rheological properties.

## Material and Methods

 A literature review was carried out using the pubmed search engine with the keywords: temporary filler materials, hyaluronic acid, physicochemical properties, rheology; the inclusion criteria were: articles written in English, articles with full text and articles written in the last 10 years.

## Results

 Scientific evidence classifies filler materials according to rheological and physicochemical properties. The most important are HA (hyalironic acid) concentration and cross-linking technology and the main parameters among them are the elastic modulus or G' factor, the tan δ (G''/G') and the complex modulus or G factor. Of these, the G' factor is the most important. There are very few current studies on temporary filler products other than HA, due to the large commercialisation and use of HA in recent years. HA fillers show remarkable cosmetic efficacy and efficacy in the treatment of various skin volume defects.

## Conclusions

 differentiate products by their rheological and physicochemical rheological and physicochemical properties can serve as a useful way to select which products are most suitable for a given clinical need, as the rheological evaluation of HA fillers is a prerequisite for the selection of the most suitable product for a given clinical need.

# Abstract: Coronectomy as an alternative treatment to exodontics of included lower third molars

**Authors:** A. Carracedo Álvarez, G. Díaz Bastos, L. Varela Estévez, J. De Elío Oliveros, M. Del Canto Pingarrón, M.A. Alobera Gracia

**Affiliations:** *Master in Oral Surgery, Implantology and Periodontics from the University of León*

## Introduction

 Extraction of the lower third molar is usually the most common procedure in the field of oral surgery. In addition to being a piece with a greater probability of inclusion, its roots can be in close relationship with the lower dental nerve, which can lead to numerous post-surgical complications, such as paresthesias. As an alternative to reduce the risk of complications, coronectomy arises, a technique that consists of extracting only the coronal part of the tooth, maintaining the roots in the alveolar bone. Several authors have described precise indications for using this technique and they must be strictly followed.

## Objectives

 To know the effectiveness of the coronectomy technique and to assess whether there are fewer complications.

## Material and Methods

 Literature search using the PubMed database.

## Conclusions

 It has been observed that coronectomy in lower third molars is a safe, effective, predictable technique with few complications compared to the complex extraction technique and thus avoids damage to the lower dental nerve. It should only be applied to vital teeth, in patients in good health and with the consent of the patient, who must be aware of the possibility of additional surgery in the future to extract the root. There are absolute contraindications, such as the existence of an active infection that affects the root of the tooth, roots with mobility or when the tooth presents with horizontal impaction along the mandibular canal, which would greatly increase the risk of section injury.

# Abstract: Peri-implant diseases. Clinical and biological criteria and their treatment: systematic review

**Authors:** J. Martins de Campos Nascimento dos Santos, S. Cacciacane, A. Boquete Castro

**Affiliations:** *UEMC de Máster de Formación Permanente en Implantologia y Rehabilitación Oral, Barcelona*

## Introduction

 One of the most used techniques for rehabilitation of edentulous spaces is the placement of dental implants. Peri-implant diseases represent a great challenge in the long-term survival of oral implants. These are inflammatory conditions associated with a change in the oral microbiota and we can differentiate them into: peri-implant mucositis and peri-implantitis. Mucositis is considered a precursor to peri-implantitis. Both present with inflammation of the peri-implant tissue, which is reversible in mucositis, while in peri-implantitis there is loss of peri-implant bone tissue. Its etiology is multifactorial and bacteria play a fundamental role in the initiation and appearance of these diseases.

## Objectives

 The aim of this bibliographic review is to clarify the clinical parameters that define peri-implant diseases and the treatment to be carried out in each of the conditions.

## Material and Methods

 A bibliographic search of the last 5 years has been carried out in the PubMed database, the following key words have been used: "peri-implant", "diseases", "mucositis", "treatment" and "peri-implantitis".

## Results

and discussion. Peri-implant mucositis is marked by bleeding on probing without bone loss, while peri-implantitis involves radiographic bone loss. Biofilm accumulation is the main cause, and its removal improves mucosal health. Risk factors include smoking, lack of keratinized gingiva, systemic diseases like diabetes, and residual cement. Prevention and treatment focus on controlling bacterial plaque using manual or electric brushes, interdental brushes, floss, and oral irrigators. Recommended treatments include mechanical and chemical methods, such as scaling, curettage, chemical agents, photodynamic therapy, and antibiotics. Moderate to advanced peri-implantitis may require resective or regenerative surgery.

## Conclusions

. Peri-implant diseases have multifactorial origin. Early identification and treatment of peri-implant diseases with a multidisciplinary approach, including regular clinical evaluation, plaque control, and therapeutic interventions, are crucial for implant survival. The continued research of biomarkers and new technologies, including the development of artificial intelligence.

# Abstract: A review of the literature on apical implant lesions

**Authors:** A. Costas Soto, Z. Segovia García, D. Rodríguez Betancort, B. Vega Braga, M. Del Canto Pingarron

**Affiliations:** *Master de cirugía, periodoncia e implantes (Universidad de León)*

## Objectives

 A review of the literature on apical lesions in dental implants reveals a multifactorial etiology, including contamination during placement, poor bone quality, and pre-existing infection.

## Material and Methods

 A review of the literature on apical implant lesions

## Results

 These lesions can be classified as inactive or infected, with the latter requiring surgical intervention and potential implant removal. Clinical manifestations include pain, inflammation, and radiographic evidence of infection. Early diagnosis and treatment are crucial for implant survival. Management options include periapical surgery with curettage and irrigation.

## Conclusions

 Some authors propose a histopathological classification and treatment decision tree for implant periapical lesions, highlighting the need for a comprehensive approach to managing this condition.

# Abstract: Guided Bone Regeneration Surgery

**Authors:** C. Méndez, B. Cabrera, C. Espinoza

**Affiliations:** *Master de Implantologia y Periodoncia de la Escuela Internacional de Posgrado*

## Objectives

 Beside the horizontal bone loss following extraction, height is also reduced. Nowadays, various surgical procedures are available to correct these deficiencies for posterior implant rehabilitation. The aim of the present study is to compare the success rate of Segmental Sandwich Osteotomy (SSO) vs guided bone regeneration with PTF membrane in pre-implant surgery, focusing on the survival of the regenerated area in vertical loss cases. The aim of the present study is to compare the success rate of Segmental Sandwich Osteotomy (SSO) vs guided bone regeneration with PTF membrane in pre-implant surgery, focusing on the survival of the regenerated area in vertical loss cases.

## Material and Methods

 The search strategy involved searching the electronic databases of MEDLINE, Pubmed, Embase, Scopus, Web of Science, Trip, Cochrane Oral Health Group's Trials Register, Cochrane Central Register of Controlled Trials y ProQuest Dissertations & Theses from 2006 to the present day.

## Results

 Despite the extensive possibilities of bone regeneration, we should aim to perfectionate one or two techniques. GBR appears to be the surgically easiest technique of the two studied in this review, even if it has more post-op complications. SSO is a sensitive technique that depending on the area could have unpleasant long-term complications.

## Conclusions

 When the aim is a vertical increase of alveolar bone, both procedures have stable and predictable results. However, to choose between the two of them, will rely on the buccal conditions and the clinical level of expertise.

# Abstract: Ceramic implants

**Authors:** N. Villarreal Medina Sandra, A. Salas García, E. Bueno, A. Bowen Antolín

**Affiliations:** *Master's Program in Implantology and Periodontology, Universidad Isabel I*

## Objectives

 The aim of this review is to provide a comprehensive synthesis about ceramic implants and how it works.

## Material and Methods

 An electronic search is performed in Pubmed until June 2024 using the following key words and MeSH terms without time periods: "zirconia implants" or "osseointegration" or "biological properties" or "titanium implants".

## Results

 We have selected 8 different articles, including a systematic review and one metanalisys. Zirconia in recent years has emerged as a dental material with excellent aesthetic and mechanical properties. In addition to its surface, the adhesion of bacteria is significantly reduced, which is a fundamental point in implantology

## Conclusions

 Studies have reported the influence of zirconia as a much more aesthetic alternative to titanium implants. Previous studies also disclosed that these.types of implant have more fracture resistance and mechanical stability. Besides significantly less bacterial accumulation leading to less peri-implantitis wich is linked to better osseointegration

# Abstract: Denosumab and osseointegration in postmenopausal osteoporosis in oral implantology

**Authors:** R. Perdomo Estupiñán, L. Cabrera Pérez, J. Calatayud Sierra, A. Bowen Antolín

**Affiliations:** *International Graduate School Isabel I University*

## Objectives

 The objective of the study is the evaluation of the role of Denosumab in osseointegration of dental implants in patient with postmenopausal osteoporosis. Denosumab is a human monoclonal antibody (IgG2) that targets and binds with high affinity and specificity to RANKL (receptor activating ligand for nuclear factor kappa B), preventing the activation of its receptor, RANK, on the surface of the precursors of osteoclasts and in osteoclasts. Preventing the RANKL/RANK interaction inhibits formation, function and survival of osteoclasts, in bone replacement causes decreased resorption in trabecular and cortical bone.

## Material and Methods

 Narrative bibliographic review of scientific articles in indexed journals and systematic reviews providing a detailed and solid vision of the impact of Denosumab on the osseointegration of dental implants

## Discussion

: Denosumab has a positive effect on the osseointegration of dental implants, especially in postmenopausal osteoporosis patients. However, the risk of osteonecrosis of the jaws is a significant factor that must be considered always.

## Conclusions

 Denosumab is a promising alternative to improve osseointegration in patients with significant bone loss. It is crucial to take into account the potential benefits and risks, performing close long-term follow-up of these patients. Future research should focus on strategies to mitigate side effects of Denosumab in order to optimize its use in clinical practice. Therefore, Denosumab not only provides substantial benefits in osteoporosis treatments, also improves stability and success of dental implants, outperforming bisphosphonates in fractures prevention and quality of osseointegration.

# Abstract: Immediate Placement and Loading of Implants in The Esthetic Zone

**Authors:** A. Batres Navarro, S. Cacciacane, A. Boquete

**Affiliations:** *ESIRO Barcelona UECM*

## Objectives

 The aim of this study is to determine and analyze different parameters such as PES, implant success rate, buccal bone resorption in immediate vs delayed implant placement in the maxillary esthetic zone, as well as immediate provisionalization vs delayed loading.

## Material and Methods

 Electronic Medline PubMed search with inclusive criteria of immediate implants" "Esthetic zone", meta analysis and systematic reviews were evaluated. Exclusion of more than five years and immediate implants in other areas. The focus was on immediate implant success with and without immediate loading at intervals of one to five years, PES, buccal bone resorption.

## Results

 3 meta analysis reports were reviewed. Immediate provisionalization demonstrated a statistically significant difference [MD] =1.54, 95% confidence interval (CI): 0.82–2.27], P < 0.0001). Mesial papillary recession resulted in a non significant difference, however distal papillary recession showed a significant difference(MD = −0.32, [95% CI: −0.50–−0.13], P = 0.0007). Survival rate of immediately placed implants in the esthetic zone resulted in 98.9 (97.8–99.5) % after 1 year, 96.8 (93.6–98.4) % after 2 years, and 94.8 (89.6–97.4) % after 5 years. Papilla height showed an effect size of -0.71(−1.25, −0.1) mm, mid-facial recession change of −0.15 (−0.66, 0.36) mm, and a 0.82 (0.37, 1.28) gain in PES.

## Conclusions

 Immediate implants accompanied by immediate provisionalization in the esthetic zone have become a great alternative to delayed implants. Although, the results have shown that after the first 12 months there are significant changes in the bone, results are not clinically noticeable. Survival rates of immediately placed and loaded implants have a high survival rate for up to five years, however future research must be conducted to establish baseline esthetic parameters in order to be able to have an evidence based protocol for immediate placement and loading of implants in the esthetic zone.

# Abstract: What options do we have for the emergency profile? Custom vs Standard Abutment

**Authors:** E. Ibáñez Prieto, A. González García, K. Ivaylova Serkedzhieva, L.M. Saez Alcaide, C. Pérez López

**Affiliations:** *Máster en Cirugía Bucal e Implantología UCM*

## Objectives

1. Evaluate the differences between custom and standard abutments available in the literature. 2. Study the different material options available to produce customized abutments.

## Material and Methods

 A bibliographic search was done in the Pub-Med/Medline, Science Direct, Google Scholar, and Scopus databases. It included articles in English and Spanish related to the topic to be treated. The keywords used were: "dental implant-abutment design" OR "implant abutment" OR "customized healing abutment" AND "wound healing" OR "emergence profile" OR "soft tissue management". Studies on custom abutment design, materials used for custom abutments, and comparison of custom abutments versus standard abutments were included.

## Results

 Several studies including materials such as PEEK, PMMA, zirconia, resin composite, and titanium were selected, mainly for the fabrication of custom abutments, which allowed us to evaluate several of their characteristics to decide on their fabrication. In addition, comparisons were sought in the literature between customized and standardized abutments.

## Conclusions

 According to the articles reviewed, custom abutments promote healing and preserve the soft tissue and its contours allowing a natural and aesthetic emergence profile surrounding dental implants. More studies are needed to provide the best clinical outcome and a better understanding by dentists.

# Abstract: Comparative study of antibiotic use in implant dentistry versus placebo

**Authors:** P. Maguregui Ortiz, P. Revuelta Cortés, S. Garcia Rodriguez, J. Cortés Bretón, S. Saiz Carrasco

**Affiliations:** *Universidad Complutense de Madrid*

## Introduction

 The use of antibiotics in dentistry for the treatment of infections is commonly used. However, the abuse or incorrect use of antibiotics can lead to bacterial resistance in the body. Due to the success of dental implants, they are increasingly used for the rehabilitation of lost teeth. Medication was introduced with the aim of decreasing the risk of postoperative complications and increasing the success rate of implants (Adell and Branemark, 1985). Different variants of antibiotic regimens have been proposed over the years, but there is a lack of standardisation in implant dentistry by dental professionals (Rodríguez Sánchez et al, 2020). Therefore, a current and valid antibiotic regimen in implant dentistry is essential.

## Objectives

 The aim of this study was to compare the success of antibiotic therapy after implant placement with a placebo.

## Material and Methods

 A literature search was carried out in the PUBMED database with the following keywords: "Dental Implants" AND "Antibiotics" AND "Placebo". We included studies carried out with antibiotics and placebo in healthy patients over 15 years of age who needed to be rehabilitated with dental implants.

## Results

 83 articles were obtained and following the inclusion and exclusion criteria, 7 articles were finally selected for the study.

## Conclusions

 The use of postoperative antibiotics does not improve the usual complications (pain, mucosal appearance, trismus, etc.) nor does it affect the variation in peri-implant marginal bone loss. There is no scientific evidence for a specific antibiotic use and the use of postoperative antibiotics is not related to successful survival of dental implants. Further clinical studies are needed to reach an agreement regarding postoperative medication after implant placement.

# Abstract: Intraoperative complications in conventional dental implantology. Bibliographic review

**Authors:** J. Bartolomé Lechuga, M. Santmartí Oliver, E. Ibáñez Prieto, E. Cáceres Madroño, C. Cobo Vázquez

**Affiliations:** *Máster en Cirugía Bucal e Implantología. Universidad Complutense de Madrid*

## Objectives

 The aim of this work is to record the intraoperative complications described in the literature in dental implantology in the last 5 years.

## Material and Methods

. For this purpose, we proposed a literature review in the pubmed database for the last 5 years, with the words "Intraoperative complications" AND "Dental implants", for which a total of 92 results were obtained, of which 16 were accepted after passing the inclusion criteria.

## Results

 The most frequent complication described was implant loss, as well as donor site morbidity or maxillary sinus injury, in addition to loss of sensation of donor tissues and lower dental nerve injury.

## Conclusions

 The complications published in scientific articles represent a limited part of those actually produced in daily practice, as few adverse events are described in the literature for the large number of oral surgical treatments performed. It is recommended to establish an adequate registry of complications in oral surgery and implantology that would allow the professional to be aware of and be prepared for complications in daily practice.

# Abstract: New approaches in the management of peri-implant marginal bone loss

**Authors:** A. Díaz Rodríguez, C. Chamorro Petronacci, G. Vianna Camolesi, J. Medina, A. García García, M. Pérez Sayáns

**Affiliations:** *Master in oral medicine, oral surgery and implantology. University of Santiago de Compostela*

## Objectives

 The objective of this work is to describe, from a reflexive point of view, the implication of all these factors in the control of MBL.

## Material and Methods

 General and systemic factors of the patient have been described, surgical factors associated with the actual placement of the implant or the surrounding hard and soft tissues, but also prosthodontic and occlusion factors. The control of the position of the implant and the volume of the tissues, of the primary stability, of the postoperative and late inflammation, as well as the moment of the load, the type of prosthesis or the shape and height of the abutment, are some of the factors currently identified as key.

## Results

 Peri-implant marginal bone loss (MBL) is a non-infectious remodelling process that occurs during the first year after the placement of a dental implant and even today, it remains a complex process that continues to raise questions.

## Conclusions

 The association between MBL and peri-implant diseases is still not entirely clear, however, some authors have established it. For this reason, early control of MBL may be a key factor in the prevention of peri-implantitis.

# Abstract: Current Micromorphological Characteristics of Dental Implants: A Literature Review

**Authors:** A. Báguena Pérez-Crespo, J.C. Báguena Gómez, A.I. Nicolás Silvente

**Affiliations:** *Servicio de Cirugía Maxilofacial Hospital Universitario Virgen de la Arrixaca de Murcia, Unidad de Patología y Terapéutica Dental. Facultad de Medicina y Odontología. Universidad de Murcia*

## Objectives

 It has been demonstrated that the characteristics of the implant surface play a fundamental role in the process of osseointegration. There is growing interest in studying and creating new surfaces that promote rapid osseointegration. The ability of body cells to adhere to the implant surface is low, so to increase the biocompatibility of the materials and thereby improve the healing and osseointegration process, modifications must be made to the chemical composition, surface roughness, and topographic characteristics. Recently the objective of numerous studies has been to establish the optimal degree of roughness to promote implant osseointegration by increasing the osteoblasts' ability to form bone. The objective of this review is to analyse the recent literature on the main micromorphological characteristics of current dental implants.

## Material and Methods

 Bibliographic review

## Results

 Some authors report that microroughness values between 1 and 100 µm favor osteoblast activity compared to smaller roughness. Currently, there are various methods to treat the implant surface to achieve this optimal roughness, including machining processes, micro-sandblasting with metals or metal oxides, etching (acid or non-acid), processes that combine sandblasting and etching, anodizing, surface coating with spherical microparticles like hydroxyapatite or metallic compounds (such as titanium), or biomimetic coatings.

## Conclusions

 Recent research primarily analyses the role of surface characteristics in promoting osseointegration. However, determining if these effects are due to surface topography or chemical composition is challenging, as they are correlated. Most implants currently on the market have a roughness between 1 and 2 µm. Therefore, surface characteristics significantly influence the success and survival rate of the implant. Given the various surface treatments for implants to achieve specific topographical characteristics, it is crucial to study the surface's specific features and chemical composition after each treatment. This enables accurate characterization and the selection of the most beneficial treatment.

# Abstract: How Interimplant Distance Affects Bone Ridge Height Using Platform-Switched Implants

**Authors:** J. Barrantes, B. Tejedor, A. Torrejon, V. Schiavo, J. López

**Affiliations:** *Máster Medicina, Cirugía e Implantología - Universitat de Barcelona – Facultad de Medicina y Ciencias de la Salud [Odontología]. C/ Feixa LLarga s/n. Hospitalet de Llobregat, Barcelona, 00907*

## Objectives

 To review existing literature to evaluate how interimplant distance influences bone crest height when using platform-switched implants.

## Material and Methods

 PUBMED, SCIENCE DIRECT, SCOPUS and SCIELO databases were consulted, using keywords such as: "distance between implants", "height of the bone crest" and "changed platform".

## Results

 20 articles available in indexed journals were reviewed that talk about the distance between implants and the use of platform-changed implants in relation to the height of the bone crest.

## Conclusions

- It is important to consider the distance between implants for bone preservation as it leads to better support for soft tissues and improves the crown-implant relationship. - Implants restored using the platform-switched concept show lesscrestal bone loss compared to implants restored with a standard protocol. - The literature review suggests that the distance between implants plays a crucial role in preserving bone crest height when platform-shifted implants are used.

# Abstract: Use of short implants for rehabilitation of atrophic jaws: literature review

**Authors:** H.M. Cardoso Gonçalves, S. Cacciacane, A. Boquete Castro

**Affiliations:** *UEMC de Máster de Formación Permanente en Implantología y Rehabilitación Oral, Barcelona*

## Objectives

 The aim of this bibliographic review is to analyze rehabilitation with short implants, their advantages and disadvantages, as well as surgical and prosthetic protocols.

## Material and Methods

 A bibliographical search was carried out over the last 6 years in the PUBMED database, using the following keywords: "atrophic maxilla", "short implant", "standard implant" and "survival".

## Results and Discussion

: There is currently no consensus to define a short implant, but according to the authors it can vary between 6mm and less than 10mm. Short implants present a survival rate very similar to wide implants after five years. Furthermore, the use of short implants avoids bone injection surgeries in atrophic areas, reduces treatment time, economic costs and intraoperative morbidity, compared to bone volume increase techniques. The disadvantages are more sensitive to bone loss and torsional strength and obtain worse results in cases of type IV. From a surgical point of view, it is essential to obtain primary stability and the use of larger implants is recommended to increase the implant-to-bone contact surface, as is the use of implants with a treated surface. From a prosthodontic point of view, it is recommended to use short implants with internal connection, with a change in platform. Correct inclusion avoids lateral forces and recommends fertilization of implants whenever possible to reduce tensions exerted on the peri-implant bone.

## Conclusions

 Short implants are presented as a predictable treatment alternative and with similar success rates to wide implants. It constitutes the best alternative in cases of need for bone regeneration techniques in atrophic jaws. Future research is necessary to explore the potential of short implants, in order to optimize the techniques and maximize the benefits of their application.

# Abstract: Peri-implant diseases. Clinical and biological criteria and their treatment: bibliographic review

**Authors:** J. Martins De Campos Nascimento Dos Santos, S. Cacciacane, A. Boquete Castro

**Affiliations:** *UEMC de Máster de Formación Permanente en Implantologia y Rehabilitación Oral, Barcelona*

## Objectives

 The aim of this bibliographic review is to clarify the clinical parameters that define peri-implant diseases and the treatment to be carried out in each of the conditions.

## Material and Methods

. A bibliographic search of the last 5 years has been carried out in the PubMed database, the following key words have been used: "peri-implant", "diseases", "mucositis", "treatment" and "peri-implantitis".

## Results and Discussion

 Peri-implant mucositis is clinically characterized by the presence of bleeding on probing, without loss of bone support, while in peri-implantitis the loss of supporting bone is observed radiographically. Biofilm accumulation is the main etiological factor and its removal improves the health of the peri-implant mucosa. Other risk factors are smoking, the amount of keratinized gingiva, the presence of systemic diseases such as diabetes and the presence of remaining cement. For its prevention and treatment, correct control of bacterial plaque is essential, eliminating the biofilm without damaging the surface and the abutment of the implant. To do this, the patient will use manual or electric brushes, interdental brushes, dental floss and/or oral irrigators. Clinically, mechanical and chemical treatments are recommended, such as scaling and curettage, use of chemical agents, photodynamic therapies, systemic and local antibiotic therapy. In cases of moderate and advanced peri-implantitis it will be necessary to use resective, regenerative surgery techniques or a combination of both.

## Conclusions

 Peri-implant diseases have multifactorial origin. Early identification and treatment of peri-implant diseases with a multidisciplinary approach, including regular clinical evaluation, plaque control, and therapeutic interventions, are crucial for implant survival. The continued research of biomarkers and new technologies, including the development of artificial intelligence, represent fundamental tools for changing the survival of implants.

# Abstract: Customized healing abutments for dental implants

**Authors:** F. Salelles Roman, S. Cacciacane, A. Boquete

**Affiliations:** *ESIRO BARCELONA-UEMC*

## Objectives

 The aim of this narrative review is to determine the clinical and radiological impact of the placement of a customised healing abutment on a dental implant.

## Material and Methods

 A narrative review was carried out using the keywords: "abutments", "healing", "customised", "implants" and "dental" in the PubMed database of the scientific literature published in the last 10 years on customised healing abutments in dental implants and their effects on the prevention of hard and soft tissue collapse and bone regeneration.

## Results

 The use of a temporary customised healing abutment helps prevent hard and soft tissue collapse and promotes bone regeneration. The placement of a customised healing abutment will in turn allow for an optimal aesthetic restoration of the implant.

## Conclusions

 This narrative review shows the different uses of custom healing abutments and the favourable results compared to standard abutments. Both prefabricated and in-office customised abutments are available, allowing the soft tissue to mature more naturally and producing aesthetic and functional implant-supported restorations.

# Abstract: Rehabilitation with implants in maxillary anatomical buttresses

**Authors:** U. Alonso Uribarri, M. Bandera Ortiz, E. Garcia Callejas, E. Nuñez Marquez, J.L. Rondón Romero, A. Hernández Suarez

**Affiliations:** *Master of Oral Implantology. University of Seville*

## Objectives

 Review rehabilitation with implants in maxillary anatomical buttresses

## Material and Methods

 A search was conducted in databases such as PubMed, Medline, and Scielo. They are discarded studies that were not relevant or not in English or Spanish and or they did not focus on the topic.

## Results

 Maxillary buttress-supported implant rehabilitation is an advanced technique in dentistry aimed at restoring functionality and aesthetics in patients with severe tooth loss. Maxillary buttresses, anatomical structures of the upper jaw, are utilized as anchorage points for dental implants, particularly beneficial in cases of significant bone loss. The technique involves placing implants in specific areas of the upper jaw, leveraging the bone density of regions such as the maxillary tuberosity, and zygomatic process. This approach ensures proper distribution of masticatory forces, enhancing the stability and durability of the prostheses. The advantages include increased retention and stability of prostheses, improved masticatory function and aesthetics, and a long-term solution for tooth loss. Additionally, this technique can obviate the need for extensive bone grafting procedures, thereby reducing treatment time and patient discomfort.

## Conclusions

 In summary, maxillary buttress-supported implants represent an effective and less invasive option for oral rehabilitation in patients with tooth loss and bone deficiencies in the maxilla.

# Abstract: Zygomatic implants: indications and clinical considerations

**Authors:** B. Buceta Martin, N. Marquez Bautista, M. Romero Barquilla, J.L. Rondón Romero, N. Matos Garrido, I. Ortiz Garcia

**Affiliations:** *Master of Oral Implantology. University of Seville*

## Objectives

 Review the indications of zygomatic implants

## Material and Methods

 A search was conducted in databases such as PubMed, Medline, and Scielo. They are discarded studies that were not relevant or not in English or Spanish and or they did not focus on the topic.

## Results

 Conventional grafting with autogenous bone has been considered the "gold standard" in the treatment of extremely atrophic jaws, but due to high failure rates (10-30%), additional time and higher costs, the development and introduction of new protocols with similar clinical results is warranted. The introduction of implants in the zygomatic bone as an anchorage point for prosthetic rehabilitation in patients with maxillary defects has been successfully developed. These implants are machined, self-tapping surface screws in commercially pure titanium that feature a 45° angled prosthetic head to compensate for the angulation between the zygomatic bone and the alveolus. These implants, by increasing their length with ranges from 30 to 52.5 mm, are indicated in cases with maxillary bone atrophy or deficiency, previous failed treatments with grafts and/or implants, avoidance of staged bone grafting procedures and conditions that may complicate traditional bone grafting procedures, such as benign cysts and trauma.

## Conclusions

 In any case, the choice of treatment will depend on the patient's characteristics, the amount of residual bone, the overall risks and the patient's wishes.

# Abstract: Particulate dentin graft as a bone substitute

**Authors:** I.A. Bueno Bianchi, I. Mora Izquierdo, I. Ortiz Garcia, A. Jiménez Guerra, L. Monsalve Guil, E. Velasco Ortega

**Affiliations:** *Máster de Implantologia Oral. Universidad de Sevilla*

## Objectives

 Review the use of particulate dentin graft as a bone substitute.

## Material and Methods

 A search was conducted in databases such as PubMed, Medline, and Scielo. They are discarded studies that were not relevant or not in English or Spanish and or they did not focus on the topic.

## Results

 Autologous dentin grafting involves using extracted teeth from the patient as a material for bone regeneration. This process includes cleaning, grinding, and demineralizing the teeth, removing hydroxyapatite crystals to release essential growth factors. Demineralized dentin contains type I collagen and bone morphogenetic proteins (BMP-2), which promote new bone formation. The advantages of using autologous dentin include high cellular compatibility, rapid bone regeneration, and the absence of rejection or disease transmission risk. Additionally, it is an economical procedure with minimal morbidity, as it does not require a second surgery to obtain graft material. Clinical studies have shown that demineralized dentin grafts are effective for alveolar preservation and ridge regeneration, showing comparable results to materials like mineralized bovine bone. Autologous dentin grafting is a promising technique in regenerative dentistry, leveraging the patient's own biological resources to improve clinical outcomes and optimize bone regeneration.

## Conclusions

 This technique offers an effective and biocompatible solution for various dental procedures, standing out for its simplicity and effectiveness.

# Abstract: New advances in the use of tricalcium betaphosphate in oral implantology

**Authors:** K. Cano Vitokhina, M. Díaz Perez, J. Moreno Muñoz, E. Nuñez Marquez, J.L. Rondón, A. Jiménez Guerra

**Affiliations:** *Máster de Implantología Oral. Universidad de Sevilla*

## Objectives

 The objective of this study is to evaluate the clinical results of the use of beta-tricalcium phosphate for the regeneration of alveolar locations prior to implant surgery and its subsequent prosthodontic rehabilitation.

## Material and Methods

 A bibliographic search has been carried out in Pubmed during the last 5 years.

## Results

 The results of this study demonstrate that dental implants can be inserted into areas regenerated with tricalcium betaphosphate with similar success to conventional surgery and obtain adequate osseointegration for their functional load. Likewise, the use of tricalcium betaphosphate has shown good clinical results in surgery, periodontics and oral implantology. Various studies have demonstrated the advantages of using beta-tricalcium phosphate as a biomaterial in those patients who needed maxillary sinus elevation for the subsequent insertion of dental implants.

## Conclusions

 The surgical approach in various clinical situations (postextraction sockets, crest expansion and transalveolar/lateral elevation of the maxillary sinus) with areas regenerated with tricalcium betaphosphate allows the insertion of the implants in a safe and predictable manner with a low prevalence of complications, achieving a high rate of success.

# Abstract: The sausage technique for horizontal augmentation of the alveolar ridge

**Authors:** M. Cerra González, B. Buceta Martin, M.D. Corona Pineda, L. Monsalve Guil, L. Matos Garrido, J. Moreno Muñoz

**Affiliations:** *Master of Oral Implantology. University of Seville*

## Objectives

 Review the sausage technique for horizontal augmentation of the alveolar ridge

## Material and Methods

 A search was conducted in databases such as PubMed, Medline, and Scielo. They are discarded studies that were not relevant or not in English or Spanish and or they did not focus on the topic

## Results

 Rehabilitation using implants is one of the most used techniques to restore the patient's aesthetics and functionality lost due to the total or partial absence of teeth. In some cases, this absence maintained over time or other associated factors causes a decrease in the dimensions of alveolar ridge, that is not sufficient for the insertion of implants. In recent years, new efficient techniques have been implemented to control this lack of bone and have an adequate bed. One of them is known as the "sausage technique", with which we achieve a alveolar increase that will prepare the edentulous area as ideal to achieve adequate stability, in addition to being able to achieve a decrease in morbidity since bone substitutes are used so there is no donor area, this causes better patient acceptance and recovery

## Conclusions

 The sausage technique is useful for bone augmentation if the case is appropriate.

# Abstract: Differences between immediate implant placement technique with immediate loading vs. delayed implants

**Authors:** M.D. Corona Pineda, N. Márquez Bautista, P. Sánchez Climent, N. Matos Garrido, L. Monsalve Guil, J. Moreno Muñoz

**Affiliations:** *Master of Oral Implantology. University of Seville*

## Objectives

 This communication will present a review of the literature on the comparison between the two techniques, showing the benefits and differences between them.

## Material and Methods

 A search was conducted in databases such as PubMed, Medline, and Scielo. They are discarded studies that were not relevant or not in English or Spanish and or they did not focus on the topic

## Results

 The immediate implant placement technique was introduced as an alternative to the classic delayed or deferred placement of implants in the healed alveolar bone.Thanks to this technique, the time intervals have been shortened, allowing the implants to be placed immediately after the implants to be placed immediately after extraction, in order to reduce the dimensional changes of the alveolus.

## Conclusions

 The results of some studies suggest that delayed implant placement may result in a slightly higher survival rate, however, it may reduce esthetic results and increase treatment time and therefore patient discomfort.

# Abstract: New trends in the bone regeneration in implant dentistry

**Authors:** M. Díaz Perez, K. Cano Vitokhina, A. Hernández Suarez, I. Ortiz Garcia, L. Monsalve Guil, E. Velasco Ortega

**Affiliations:** *Máster de Implantología Oral. Universidad de Sevilla*

## Introduction

 The success of treatment with dental implants will depend on an adequate quantity and quality of bone. Tooth loss, periodontal disease, periapical infections, cysts, trauma, etc., can lead to a lack of alveolar bone, making it necessary to resort to techniques to increase bone volume such as the use of bone regeneration techniques with membranes. and biomaterials.

## Objectives

 The objective of the present study is to assess the scientific evidence of bone regeneration through the use of different materials.

## Material and Methods

 A bibliographic search was carried out in Pubmed relating to the last five years.

## Results

 Autologous grafts usually show the greatest bone gain, but are usually associated with more adverse effects and complications at the donor site. Platelet-rich materials may also be useful. Grafts of animal origin and synthetics are widely used today. More recently, 3D printed materials are being incorporated to improve the effectiveness and safety of guided bone regeneration.

## Conclusions

 The treatment of bone defects is one of the most important challenges in the field of implantology. The various biomaterials aim to treat bone defects and stimulate the formation of new bone due to their osteoconductive capacity; however, bone regeneration will depend greatly on the skill of the professional and the management of soft tissues.

# Abstract: Challenges and strategies in the treatment of peri-implant soft tissue recessions: classification and surgical techniques

**Authors:** M. Garcia Gallego, D.M. Lozano Fraile, M. Pérez Chaves, A. Jiménez Guerra, J.L. Rondón Romero, I. Ortiz Garcia

**Affiliations:** *Master's Degree in Oral Implantology. University of Seville*

## Objectives

 The following poster discusses the specific challenges of treating peri-implant soft tissues recessions (PSTD) on dental implants compared to the efficacy of root coverage procedures for the treatment of gingival recessions in natural teeth.

## Material and Methods

 A search was conducted in databases such as PubMed, Medline, and Scielo.

## Results

 While in natural teeth root coverage is limited by the position of the interproximal insertion levels, PSTDs on implants present additional complications such as improper implant placement, lack of buccal bone, and connective tissue vascularization that resembles healing tissue. A classification of PSTD based on the bucco-lingual position of the implant and the dimension of the interproximal papillae is proposed, with four classes and three subcategories. For each class, specific surgical techniques such as the coronal advanced flap and the use of connective tissue grafts are recommended to increase soft tissue thickness and improve aesthetics. The study underlines the need for further studies to validate the recommendations and improve treatment predictability considering the aesthetics demanded by patients, emphasizing patient satisfaction, long-term implant survival along with functionality, tissue stability and patient satisfaction. In addition, it highlights a study that found a significant prevalence of PSTD dehiscence, identifying risk factors such as, buccal malposition of the implant and a thin peri-implant phenotype.

## Conclusions

 It is essential to develop effective protocols to manage aesthetic defects in implants, ensuring both functionality and aesthetic satisfaction of patients.

# Abstract: The Importance of the Peri-Implant Phenotype for Achieving Stable Aesthetics and Volume in Soft Tissue Regeneration and Grafts

**Authors:** I. Izquierdo Mora, A.I. Bueno Bianchi, S. Tristán Carbonell, J.L. Rondón Romero, A. Hernández Suárez, N. Matos Garrido

**Affiliations:** *Master's in Oral Implantology. University of Seville*

## Objectives

 review The Importance of the Peri-Implant Phenotype for Achieving Stable Aesthetics and Volume in Soft Tissue Regeneration and Grafts.

## Material and Methods

 A search was conducted in databases such as PubMed, Medline, and Scielo. They are discarded studies that were not relevant or not in English or Spanish and or they did not focus on the topic

## Results

 A crucially important situation in implant rehabilitation is the management of the peri-implant phenotype, from the moment of diagnosis and case planning to the handling of grafts and soft tissues during surgery. It is necessary to assess the possible techniques and biomaterials to achieve the desired regeneration, aiming for functional and aesthetic results. It should not be forgotten that soft tissues are essential to maintaining and ensuring the success of the implant. The immediate post-extraction implant technique helps to maintain the patient’s natural gingival structure, although it requires a thorough surgical protocol and good execution with the grafts, as well as prosthodontic restoration with attachments that have correct emergence profiles to ensure long-term stability. Furthermore, it is important to raise awareness and secure the patient's participation.

## Conclusions

 The patient must understand that it is not advisable to chew hard foods during the first few weeks, that they should not smoke, and that brushing in the operated area should be postponed for 15 days to avoid moving the graft, while maintaining proper hygiene using chlorhexidine mouthwash.

# Abstract: The socket shield technique in implant dentistry

**Authors:** D. Joaquin Vilanova, M. Bandera Ortiz, J.J. Sauco Carballo, A. Jiménez Guerra, J.L. Rondón Romero, A. Hernández Suarez

**Affiliations:** *Master of Oral Implantology. University of Seville*

## Introduction

 The socket shield technique is a method of alveolar preservation within partial extraction therapies, used in the immediate placement of implants. It is characterized by preserving a vestibular portion of the tooth root when placing an immediate implant.

## Objectives

 The purpose of this review was to examine the current scientific literature to highlight the characteristics and success criteria of the technique.

## Material and Methods

 A literature review was performed in PubMed using the search terms "Socket Shield and immediate implant placement".

## Results

 It is crucial for the technique to prepare the shield with the appropriate length and thickness. A round bur is used to follow the root canal to the apex, followed by a 2-3 mm reduction with a small Lindemann bur to avoid inflammatory complications.

## Conclusions

 It was concluded that the following parameters should be followed for the success of the technique: careful case selection and execution by a specialist, evaluation by CBCT to visualize the precise root anatomy and detect possible problems such as infections. To ensure the integrity of the shield, a minimum thickness of 1.5 mm is recommended to prevent fractures and to completely remove the root apex to ensure stability during the treatment. Tapered implants with tapered threads offer superior stability. In addition, screw-retained provisional restorations are preferred, as they improve the emergence profile of the final result.

# Abstract: Short implants vs standar implants, in areas with severe bone atrophy

**Authors:** M.S. Labrass Zarouali, N. Afoumpan Poni, E. Garcia Callejas, J.L. Rondón Romero, I. Ortiz Garcia, E. Velasco Ortega

**Affiliations:** *Máster de Implantología oral. Universidad de Sevilla*

## Objectives

 Review the Short implants vs standard implants, in areas with severe bone atrophy.

## Material and Methods

 A search was conducted in databases such as PubMed, Medline, and Scielo. They are discarded studies that were not relevant or not in English or Spanish and or they did not focus on the topic

## Results

 The goal of this study is not to debate the success of short dental implants versus standard/long dental implants, but to compare short dental implants with standard/long dental implants in situations where additional bone grafting or augmentation procedures would have been necessary, and how these can avoid the need for advanced surgical procedures and their associated risks.

## Conclusions

 It can be concluded that short dental implants are a viable option in areas that would have required complex and costly augmentation procedures. Short dental implants demonstrated comparable survival and success rates, with faster, less expensive treatment, and fewer surgical complications and morbidity.

# Abstract: The use of autogenous dentin as osteogenic material

**Authors:** D.M. Lozano Fraile, S. Haidar Wehbe, B. Ramirez Ruiz, I. Ortiz Garcia, A. Jiménez Guerra, N. Matos Garrido

**Affiliations:** *Máster de Implantología Oral. Universidad de Sevilla*

## Objectives

 Review the use of autogenous dentin as osteogenic material.

## Material and Methods

 For this communication, a review of the available literature will be carried out on the use of autogenous dentin as osteogenesis material.

## Results

 In the field of oral surgery and implantology, numerous bone regeneration materials have been utilized for multiple procedures, such as alveolar preservation or maxillary sinus elevations. These materials range from the use of bone xenografts, allografts or autografts to the use of platelet-rich plasma extracted directly from the patient's blood. Currently, several studies talk about the use of autogenous dentin, obtained after performing a dental extraction on the patient and subsequent treatment of the tooth to extract the greatest amount of dentin possible.

## Conclusions

 The results of these studies affirm that autogenous dentin has osteoconductive and osteoinductive capacity, which makes it a possible option to take into account when performing this type of procedures.

# Abstract: Single implants as a therapeutic option in oral implantology

**Authors:** D. Marcolino Cruz, I. Ortiz Garcia, E. Nuñez Marquez, J. Moreno Muñoz, L. Monsalve Guil, E. Velasco Ortega

**Affiliations:** *Máster de Implantología Oral. Facultad de Odontología. Universidad de Sevilla*

## Introduction

 Single dental implants can be successfully inserted and obtain adequate osseointegration for their prosthodontic functional load, demonstrating very high survival and success after an average clinical follow-up period of several years.

## Objectives

 The objective is to analyze the existing literature on treatment in oral implantology with single implants.

## Material and Methods

 A bibliographic review of the last 5 years obtained from Pubmed, Sciencedirect and Google academic was carried out. The following keywords have been introduced: dental implants, oral implantology, internal connection, external connection, esthetics, single implants.

## Results

 Clinical experience has shown that the clinical protocol of single implants represents a type of treatment with an excellent functional and aesthetic component and a high degree of acceptance by the patient. Dental treatment with single implants can be carried out and maintained successfully. Both immediate loading and early loading of the implants, in addition to reducing surgical phases, shortening the total treatment time and reducing the loss of soft tissue and hard; These protocols allow the patient to perform all oral functions and favor the aesthetic result.

## Conclusions

 Treatment with single implants represents a predictable therapeutic option, allowing adequate osseointegration to be obtained for its prosthodontic functional load, with aesthetic and functional results.

# Abstract: Alveolar preservation in immediate post-extraction implants

**Authors:** P. Méndez Hernández, U. Alonso Uribarri, J.J. Sauco Carballo, J. Muñoz Moreno, E. Nuñez Márquez, J.L. Rondón Romero

**Affiliations:** *Master's Degree in Oral Implantology. University of Seville*

## Objectives

 Review the alveolar preservation in immediate post-extraction implants.

## Material and Methods

 A search was conducted in databases such as PubMed, Medline, and Scielo. They are discarded studies that were not relevant or not in English or Spanish and or they did not focus on the topic

## Results

 In implant restorations, the preservation of peri-implant tissues has a vital importance in order to achieve both good aesthetics and long-term stability of the peri-implant tissues. There are many cases in which the professional decides to place an implant immediately after the extraction of a tooth affected by any irreversible pathology in order to reduce the changes because remodelation in the alveolus post-extraction. In these cases there are several options to preserve the tissues and thus achieve the most natural rehabilitation possible, with the best aesthetics and more stable over time.

## Conclusions

 The use of certain biomaterials and membranes can help to achieve greater bone volume and a better soft tissue response.

# Abstract: Uncontrolled diabetes mellitus as a risk factor for peri-implantitis

**Authors:** P. Sánchez Climent, D. Corona Pineda, M. Garcia Gallego, N. Matos Garrido, L. Monsalve Guil, J. Moreno Muñoz

**Affiliations:** *Máster de Implantología Oral. Universidad de Sevilla*

## Objectives

 Review the uncontrolled diabetes mellitus as a risk factor for peri-implantitis

## Material and Methods

 A search was conducted in databases such as PubMed, Medline, and Scielo. They are discarded studies that were not relevant or not in English or Spanish and or they did not focus on the topic

## Results

 Oral rehabilitation with dental implants is the treatment of choice in the most patients with partial and total edentulism. Peri-implantitis is considered as a biological complication of dental implants that can cause their failure. Diabetes mellitus is a disease with systemic impact that has a high prevalence today. Patients with uncontrolled diabetes mellitus may have a higher risk of suffering peri-implant diseases and failure of the osteointegration of dental implants.

## Conclusions

 Several studies show that the prevalence of biological complications, such as mucositis and peri-implantitis, is higher in patients with uncontrolled diabetes and usually have more advanced stages of peri-implant pathology.

# Abstract: Dynamic navigated surgery system in oral implantology

**Authors:** E. Sanz Sanz, B. Buceta Martin, M. Cerra Gonzalez, J. Moreno Muñoz, A. Hernández Suarez, E. Velasco Ortega

**Affiliations:** *Master of Oral Implantology from the University of Seville*

## Objectives

 Review the uncontrolled diabetes mellitus as a risk factor for peri-implantitis

## Material and Methods

 A search was conducted in databases such as PubMed, Medline, and Scielo. They are discarded studies that were not relevant or not in English or Spanish and or they did not focus on the topic

## Results

 The dynamic guided navigation system, also called guided optoelectronics, allows you to perform computer-assisted surgery with optical tracking and allowing corrections in the treatment plan during the execution of the same. There are two types of optical tracking: active and passive tracking system. The active tracking system emits infrared light that is transmitted to the stereoscopic cameras, assisting 3D vision, while the passive tracing system uses reflective spheres to the infra-red light projected from light diodes to a tracking camera.

## Conclusions

 Planning is the most important part of ensuring predictability and accuracy of surgery, with the same preoperative tests, the same planning and digital design processes required.

# Abstract: Primary stability of implant placement and loading related to dental implant materials and designs: a literature review

**Authors:** D.M. Tejada Medina, E. Sanz Sanz, M. Cerra González, E. Nuñez Marquez, A. Hernandez Suarez, A. Jiménez Guerra

**Affiliations:** *Máster de Implantología Oral. Universidad de Sevilla*

## Objectives

 Review the primary stability of implant placement and loading related to dental implant materials and designs: a literature review

## Material and Methods

 A search was conducted in databases such as PubMed, Medline, and Scielo. They are discarded studies that were not relevant or not in English or Spanish and or they did not focus on the topic.

## Results

 Often, the waiting or healing period of the implant loads after surgery, was between 3 and 6 months, depending on the upper jaw or mandible. In this sense, the functional load was established according to the primary stability of the implants, the surgical technique, the volume and density of the bone, the type of occlusion, the material and macroscopic design (conical, spirals, etc.) and the surface of the implants. The primary stability can be assessed objectively by the insertion torque and the resonance frequency analysis.

## Conclusions

 All of these factors combined, determine the primary fixation of the alveolar bone and must be taken into account by the professional, above all, for the immediate and early loading protocols.

# Abstract: Advantages, disadvantages, and complications of immediate implants

**Authors:** S. Tristán Carbonell, M. Mayoral Estrada, I.A. Bueno Bianchi, N. Matos Garrido, J.L. Rondón Romero, A. Jiménez Guerra

**Affiliations:** *Máster de Implantología Oral. Universidad de Sevilla*

## Introduction

 The placement of dental implants at the time of tooth extraction, known as immediate implants, is a viable treatment protocol in implant dentistry.

## Objectives

 The objectives of immediate implants are the same as those of conventional staged treatment: to achieve primary implant stability, ensure adequate rigid fixation after healing, optimally position the implant for restoration, obtain an ideal aesthetic outcome, and better preserve peri-implant tissues.

## Results

 The popularity of immediate implant placement has increased because it allows these objectives to be met with fewer procedures, shorter treatment times, and reduced costs for the patient. However, immediate placement implants are more demanding and require specialized skills from the professional. Both the surgical procedure and the prosthetic rehabilitation are more complex, and there are multiple factors that can increase morbidity or complications.

## Conclusions

 Therefore, this poster will discuss the advantages, disadvantages, and complications associated with the use of these implants.

# Abstract: Zirconium implants as an alternative to titanium

**Authors:** M. Bandera Ortiz, D. Joaquín Vilanova, U. Alonso Uribarri, J.L. Rondón Romero, I. Ortiz Garcia, A. Jiménez Guerra

**Affiliations:** *Master of Oral Implantology. Sevilla University*

## Objectives

 A bibliographic review is presented on the advantages and disadvantages of using zirconia implants as an alternative to the use of titanium implants.

## Material and Methods

 A search was conducted in databases such as PubMed, Medline, and Scielo. They are discarded studies that were not relevant or not in English or Spanish and or they did not focus on the topic.

## Results

 Since the beginning of implantology, comparative studies have been carried out to find the materials that have the best biocompatibility and biomechanics for oral rehabilitation; reaching the conclusion, for many years, that titanium provided these characteristics. Currently, zirconium has been used as an alternative to metal alloys for tooth-supported and implant-supported prosthetic rehabilitations, given its better aesthetics. It has also been proven that modifying the surface of the titanium implant, with zirconia, improves its properties. Knowing the biocompatible and aesthetic advantages of zirconia, would it be a good alternative to use zirconia implants instead of titanium implants? Characteristics such as inherent biocompatibility, very little affinity for dental plaque, and greater resistance to bending and fracture than other ceramic materials have made it an interesting object of study.

## Conclusions

 Currently, zirconia implants are an alternative to consider.

# Abstract: Influence of soft tissues on peri-implant health

**Authors:** E. García Callejas, M. Caballero Parejo, P. Méndez Hernández, J.L. Rondón Romero, I. Ortiz Garcia, L. Monsalve Guil

**Affiliations:** *Master Oral Implantology. Sevilla University*

## Objectives

 The objective of this poster is to analyze the role that soft tissues play in the success of implants and to develop the most current techniques for their augmentation.

## Material and Methods

 A search was conducted in databases such as PubMed, Medline, and Scielo. They are discarded studies that were not relevant or not in English or Spanish and or they did not focus on the topic.

## Results

 The integrity of the peri-implant soft tissues plays a fundamental role not only in the aesthetic aspect, but also in the stability and maintenance of the implants in the long term. The lack of keratinized mucosa can make it difficult to control dental biofilm and cause peri-implant biological complications. Soft tissue grafts may be an option to prevent initial bone loss and improve the health of peri-implant tissues. Their main clinical indications include the gain of keratinized tissue and the increase in soft tissue volume. Soft tissue grafts can be of different types, such as connective tissue grafts, epithelial tissue grafts or mixed tissue grafts, depending on the needs of the patient and the anatomy of the location.

## Conclusions

 It is essential to carry out an adequate diagnosis and treatment plan to determine the need for soft tissue grafts and select the most appropriate technique for each individual case.

# Abstract: Fracture of prosthetic connection screws in oral implantology: causes, consequences and management

**Authors:** A.P. Nicias, M. El Alluch Al Hachemi, P. Méndez Hernández, N. Matos Garrido, A. Jiménez Guerra, E. Nuñez Marquez

**Affiliations:** *Master of Oral Implantology. Sevilla University*

## Objectives

 To analyze the causes, consequences and management of prosthetic connection screw fractures.

## Material and Methods

 A search was conducted in databases such as PubMed, Medline, and Scielo. They are discarded studies that were not relevant or not in English or Spanish and or they did not focus on the topic.

## Results

 Oral implantology has revolutionized the field of dentistry, offering long-lasting and functional solutions for tooth loss. However, one of the most critical challenges in this field is the fracture of implant connection screws that can affect the treatment outcome. This phenomenon can compromise the stability of the implant and cause significant clinical complications. Screw fracture in oral implantology is a complication that requires a deep understanding of its causes and a proactive approach to its prevention, the implementation of preventive strategies and adequate management to minimize the risk of these complications appearing.

## Conclusions

 Through proper material selection, meticulous planning, and ongoing monitoring, the risk of fractures can be decreased and the long-term success of dental implants ensured.

# Abstract: Design of the ideal emergence profile for implant restorations

**Authors:** J.J. Saúco Carballo, M.S. Labrass Zarouali, D. Joaquin Vilanova, I. Ortiz Garcia, J. Moreno Muñoz, E. Nuñez Marquez

**Affiliations:** *Master of Oral Implantology. Sevilla University*

## Introduction

 The design of the emergence profile is important to achieve stable peri-implant tissues and aesthetically favorable results with future restorations and can be influenced by factors such as implant position and surrounding soft tissues. Different aspects of the emergence profile have been described, identifying the different areas of the restoration and the different designs for each situation and type of implant.

## Objectives

 This bibliographic review aims to gather the most current information on the design of the emergence profile in implant restorations.

## Material and Methods

 A search was conducted in databases such as PubMed. They are discarded studies that were not relevant or not in English or Spanish

## Results

 To achieve adequate aesthetics and function, it is necessary to correctly design the emergence profile of our implant restorations. To do this, it is essential to know the different areas of the restoration and prepare them appropriately.

# Abstract: Efficacy of Using Hyaluronic Acid for Interdental Papila Reconstruction Alone or Combined with Injectable PRF

**Authors:** S. Ayuso Casado, J.M. Acuña Pedrosa, A. Boquete Castro

**Affiliations:** *JMAP Formación Acca Learning. Universidad Europea Miguel de Cervantes*

## Objectives

 To do an updated review of the literature about the use of hyaluronic acid (HA) versus Injectable Platelet Rich Fibrin (i-PRF), alone or combined for the reconstruction of the interdental papilla with a minimal invasive approach.

## Material and Methods

 a bibliographic search was carried in PubMed and Cochrane, using the following keywords: hyaluronic acid, PRF, black triangles and interdental papilla reconstruction. Comparative in vitro and in vivo studies published in the last 10 years were selected.

## Results

 Injected HA technique showed better results in comparison with saline solutions, when factors such as no adjacent implants, nor interproximal restorations, the absence of periodontal disease and a good oral hygiene were fulfilled. There are no published studies comparing the use of HA and i-PRF individually for papilla reconstruction. Instead, it exit some articles regarding the management of thin gingival phenotype, in which case the difference between HA and i-PRF was not statistically significant.On the other hand, studies in which HA and PRF were combined showed a statistically better result in the papillary filling and stability when compared with HA alone.

## Conclusions

 the use of HA is a good alternative when factors are favorable in contrast to more invasive techniques, and that its combination with i-PRF could be beneficial.

# Abstract: Influence of alveolar infection on the success of immediate implant treatment. Updated literature review

**Authors:** T. Fleitas Ellingson, S. Cacciacane, A. Boquete Castro

**Affiliations:** *Universidad Europa Miguel de Cervantes. Máster de Formación Permanente en Implantología y Rehabilitación Oral Internacional*

## Objectives

 A review is intended to investigate whether there is evidence that indicates a greater risk of failure in the placement of immediate dental implants.

## Material and Methods

 The search was carried out in the following scientific bases: Pubmed, Scopus, WOS and Cochrane. Inclusion criteria: Studies in the last 10 years, studies based on controlled clinical trials, consecutive cohort studies, retrospective cohort studies, meta-analysis, studies that included humans where the success/failure rate is compared between immediate implant placement in infected and healthy alveoli considering a minimum follow-up of 1 year.

## Results

 The results obtained show discrepancies between them. Some studies indicate that there is a significantly increased risk in immediate implant placement in the presence of infection, while other studies reach opposite conclusions, suggesting the absence of such risk. The lack of consensus on these findings highlights the controversy that exists in the scientific literature regarding the success rate and increased risk of failure of this technique in the context of the presence of alveolar infection.

## Conclusions

 Immediate placement of a dental implant in the infected site could increase the risk of implant failure compared to that placed in infection-free sockets. More long-term studies are necessary to draw accurate conclusions.

# Abstract: Treatment of a vestibular bone defect with immediate implants and biomaterials: a case report

**Authors:** M. Fernández Varela, J.M. Acuña Pedrosa, A. Boquete Castro

**Affiliations:** *Clínica Dental Montse Varela*

## Introduction

 Scientific evidence supports the idea that the reconstruction of vestibular bone defects, along with the immediate placement of implants using bone grafts and membranes, significantly improves the long-term prognosis of implant therapy. The aim of this study is to present a clinical case of the treatment of a vestibular bone defect using biomaterials, along with the immediate insertion of two implants and the positioning of a provisional prosthesis.

## Case Report

 A 65-year-old woman with stable periodontal disease, with no relevant medical history, attends for dental aesthetic improvement. She had mobility in the lower incisors. Under local anesthesia, an intrasulcular incision was made and a full-thickness gingival flap was raised from tooth 4.3 to 3.3. Atraumatic extractions of teeth 3.1, 3.2, 4.1, and 4.2 were performed, followed by alveolar and vestibular dehiscence inspection, removing the granulation tissue, and delimitating the bone defect. Local antibiotic therapy using minocin 100 mg mixed with serum was administered. Two c-tech implants of 4.4 x 11 mm were placed with a 35 ncm torque. The bone defect and the space between the implants were filled with bone graft granules (lifenet health®). A bovine collagen membrane (lyoplant®) was adapted. The provisional fixed prosthesis previously made was connected during surgery using a provisional titanium abutment and bis-acrylic material, freeing the area in contact with the graft and occlusion.

## Conclusions

 Treatment of vestibular bone defects with grafts and membranes, along with the immediate insertion of implants, is effective and predictable. This case highlights the importance of proper planning and the advantages of using biomaterials in implantology.

# Abstract: Peri-implant mucogingival surgery: a case study

**Authors:** A. López Miranda, S. Núñez Urrutia, J.M. Acuña Pedrosa, A. Boquete Castro

**Affiliations:** *Master's Program In Oral Surgery, Implantology, And Periodontics. September 2023 / November 2024.Jmap Training, Acca Learning. Seville*

## Introduction

 A band of adherent keratinized gingiva is necessary for the health of peri-implant tissues. With bone regeneration procedures, we modify the position of the mucogingival junction, resulting in loss of vestibular depth and low positioning of muscle insertions, generating functional and aesthetic defects. The free gingival graft (FGG) allows the transfer of palatal tissue to the recipient area, restoring the position of soft tissues and increasing the keratinized tissue around the implants.

## Objectives

 To demonstrate the advantages of properly restoring peri-implant structures through a clinical case of mucogingival surgery with FGG.

## Case Report

 A 77-year-old male with relevant medical history and chronic periodontitis presents with fractures in teeth 13 and 17 and root caries in tooth 18. The treatment consisted of extractions from teeth 13 to 18, followed by bone regeneration. Subsequently, prosthetically guided Galimplant (IPX) implants were placed in teeth 13, 15, and 17. Due to the patient's age, a second phase with a vestibular repositioning flap was chosen. During healing, 2 mm of keratinized gingiva was lost, exposing mucosal and muscular insertions near the cervical transition zone of the implants. This was considered incompatible with a stable peri-implant condition, confirming the need to reposition the tissues through vestibuloplasty with a soft tissue graft. Following Zucchelli's technique, the FGG was obtained from the donor site. The recipient site of the graft was prepared by performing vestibuloplasty using the Kazanjian technique. The graft was immobilized with simple and cross sutures, and the donor site was protected with reinforced suspensory sutures with Flow ("flow shield").

## Conclusions

 The FGG is the gold standard due to its effectiveness in increasing vestibular depth and the width of keratinized tissue, presenting high success and predictability rates.

# Abstract: Photogrammetry: pic sistem. Fit and precision in the crosshairs. A case report.university

**Authors:** C. Hurtado Gil, J.M. Acuña Pedrosa, A. Boquete Castro

**Affiliations:** *JMAP FORMACION SEVILLA. Proper master's degree in continuing education in oral surgery, implantology and periodontics. Miguel de cervantes european university*

## Introduction

 Oral rehabilitation of edentulous areas isone of the most demanded treatments. Currently, there is great progress of digitalization in the dental practice, which changes the way in which cases are planned and the way patients are treated.The scientific literature describes that the correct passive adjustment improves the success of long-term rehabilitations. The photogrammetry technique uses three-dimensional coordinates to be measured using two-dimensional frames and an external system of topographic reference points.

## Objectives

 The aim of this work is to present the technique of photogrammetry through the use of the pic dental system to take impressions over implants, through the presentation of a clinical case in which 6 immediate implants are placed and immediately loaded.

## Case Report

 64-year-old man, heavy smoker. No allergies or medical history. Presents advanced periodontal disease with movility of all the lower teeth. After the clinical and radiographic analysis, it was decided to establish the following treatment plan: extractions of the remaining teeth and insertion of 6 immediate galimplant implants (ipx of 3.5, 4 and 4.5 in diameter and between 10 and 12 mm in length) in positions 32, 34.36, 42.44 and 46. After that, and due to the high torque values achieved, multiunit abutments were connected and sutured. After performing the control x-ray with the abutments attached, the pic system was used to capture the positions of the implants. After 24 hours, a provisional screw-retained prothesis was connected.

## Conclusions

 Photogrammetry is more accurate than the use of intraoral scanners to carry out digital impression over implants. It offers predictability that ensures no.

# Abstract: Unitary implants with immediate loading

**Authors:** D.F. Andreolli Infante, S. Cacciacane, A. Boquete

**Affiliations:** *ESIRO BARCELONA UEMC*

## Introduction

 Unitary implants with immediate loading in anterior sectors have gained popularity in dentistry due to their ability to provide an aesthetic and functional restoration quickly and effectively. This approach seeks to reduce treatment time and improve patient satisfaction.

## Material and Methods

 To carry out this study on single implants with immediate loading, PubMed was used as the main database to search for relevant articles. Some common keywords used in these searches include "dental implants," "immediate loading," "anterior sector," "dental aesthetics," and "long-term results." 20 articles were selected to do this review and carry out this study that meet the inclusion criteria. Factors such as implant success rate, primary stability, aesthetics, and patient satisfaction were evaluated.

## Results

 The results showed that unitary implants with immediate loading in anterior sectors presented a high success rate, with good primary stability and satisfactory aesthetic results. Additionally, patients reported high satisfaction with the treatment and rapid restoration of their masticatory function. Clinical parameters indicated good peri-implant health, with low plaque indices and average probing depths less than 3mm. Marginal bone loss was insignificant, averaging 0.2mm.

## Conclusions

 Unitary implants with immediate loading in anterior sectors are a viable and effective option for the restoration of aesthetics and function in patients who require this type of treatment. Its high success rate, combined with patient satisfaction, supports its use in clinical practice as an alternative to consider in selected cases. Proper patient selection and correct surgical planning are essential for the success of this treatment. Future research should focus on long-term evaluation of these implants and comparison with afterloading protocols.

# Abstract: Vertical bone augmentation in the posterior mandibular region; block graft from the mandibular ramus incorporating autologous bone, xenograft, and PRGF

**Authors:** S. Torres González, S. Cacciacane, A. Boquete Castro

**Affiliations:** *Centro Dental Clínica Torres*

## Introduction

 The Khoury technique is indicated for patients with extensive bone defects prior to implant placement, especially when there is less than 5-6 mm of residual bone height from the mandibular canal to the bone crest. Using autologous bone maximizes biocompatibility and graft integration.

## Case Report

 A 58-year-old non-smoking woman without any pathological conditions required the reconstruction of a posterior mandibular defect. The treatment plan was to use the Khoury technique to achieve the necessary vertical volume for the subsequent placement of two implants. Radiological studies showed less than 6 mm of bone height from the crest to the mandibular canal. During the procedure, a bone plate of approximately 20 mm horizontally and 8 mm vertically was harvested from the same side as the defect, with the subsequent placement of fixation screws. Autologous bone was collected from the area using a safescraper, combined with Creos Xenogain xenograft and PRGF. The regeneration area was covered with a Creos Xenofirm resorbable collagen membrane fixed with pushpins. After 6 months, the regenerated area showed type II bone, providing a vertical increment of 6 mm with substantial bone stability when drilling with Densah Versah bone densification burs. Three months later, two Nobel Biocare Parallel implants, measuring 3.75 x 8.5 mm and 4.3 x 8.5 mm, were loaded from anterior to posterior.

## Conclusions

 In extreme cases of posterior mandibular vertical atrophy where proximity to the mandibular canal exists, the Khoury technique is an optimal solution. This is complemented by xenograft, PRGF, and bone densification burs to achieve greater stability in the regenerated area.

# Abstract: Treatment of Bone Defect in Type 22 Furca with Emdogain

**Authors:** A.M. García Martín, C. García Tena, J.M. Acuña Pedrosa, A. Boquete Castro

**Affiliations:** *Máster Propio de Formación Permanente en Cirugía Bucal, Implantología y Periodoncia. Septiembre 2023 / Noviembre 2024. JMAP Formación ACCA Learning. Sevilla*

## Introduction

 Emdogain is an enamel matrix-derived protein compound capable of inducing true regeneration of the attachment apparatus. Its main indication is the treatment of intraosseous defects, bone gain and reduction of probing depth with minimal gingival recession and stimulates growth factors. It is a minimally invasive procedure compared to conventional regeneration techniques. The main objective of this study was to evaluate the evolution and clinical and radiographic efficacy of the use of Emdogain plus autologous bone in a mandibular grade II furcation defect (46).

## Case Report

 44-year-old patient. No medical or family history of interest, with a grade II lingual furcation defect of the lower right first molar.Measurements were taken: probing depth and radiographic horizontal bone loss, prior to treatment, at one and two years. The following sequence was followed: • Wide intrasurcular incision, from distal 43rd to distal 47th. No discharge. • Scaling and root planing and removal of granulation tissue. • Harvesting of autologous bone from the mandibular ramus with bone scraping. • Application of 37% orthophosphoric acid and subsequent copious rinsing with water and saline. • Application of Emdogain together with the collected autologous bone (to support the space, to avoid collapse). • Suture.

## Conclusions

 after 24 months of follow-up, it is observed that the probing depth was less (from 6 mm initial probing depth, to 3 mm in the revision at one year and again at two years). And the X-rays show a horizontal bone gain of 3 mm, at one and two years. Therefore, it is corroborated that the use of Emdogain favours periodontal regeneration.

# Abstract: Guided bone regeneration with autologous dentin and hyaluronic acid in Implantology

**Authors:** J. Rodríguez Méndez, L. Ahamdanech, I. Meza Acuña, F. Rodríguez Escudero, A. Bowen Antolín

**Affiliations:** *Máster de Implantología Oral. Universidad Isabel I. Escuela internacional de postgrado*

## Objectives

 The objective of this work is to highlight the use of autologous dentin as one of the materials to take into account in the future of dentistry.

## Material and Methods

 Currently, different materials are used for bone regeneration in dental clinics: allogeneic, xenogeneic, alloplastic materials and autologous bone, due to their osteogenic, osteoinductive and osteoconductive properties. Within the latter we found autologous dentin, which was used for the first time by Kim et al in 2010 demonstrating its grafts have physicochemical characteristics similar to those of autogenous bone. The placement of dental implants in regenerated sockets with autologous dentin show high stability, low level of bone loss, and in some cases has shown signs of bone gain leading to better results compared to other materials.

## Results

 Using this system compared to conventional techniques, we manage to minimize the postoperative period since it is the same area to be operated on that donate material for its regeneration. Bone healing and soft tissue response with immediate attraction of osteogenic cells. Predictable bone remodeling with an excellent density and much less recession. No rejection, inflammation or transmission of diseases. Likewise, the combination of autologous dentin with hyaluronic acid (Hyadent BG®) will provide a better dentin management, achieving its conglomerate, a faster repair of the grafted bone tissue, will help reduce the risk of infection and improve the soft tissue healing.

## Conclusions

 All this means that the combination of these promising biomaterials can become widely used in daily dental practice.

# Abstract: Peri-implant mucogingival surgery: a case report

**Authors:** C. Sánchez Salado, J.M. Acuña Pedrosa, A. Boquete Castro

**Affiliations:** *Master's degree in permanent training in oral surgery, implantology and periodontics.september 023/november 2024.jmap training acca learning*

## Introduction

 Tooth loss results in progressive bone atrophy. Consequently, the collapse of the alveolar ridge alters the soft tissues, making the final outcome of our treatment more difficult. It is important to know the indications and techniques for soft tissue regeneration to address cases where defects are minor or there are small atrophies, without the need for bone regeneration. The "Roll Flap" (RF) technique described by Abrams in 1980 allows for the reconstruction of small defects in height and width with the aim of masking them, creating a natural and harmonious tissue emergence. It involves obtaining connective tissue from the palatal area of the defect to be treated and transferring it to the vestibular flap while maintaining vascularization. The objective of this work is to present a clinical case of rehabilitation with a dental implant and soft tissue augmentation using the Roll Flap technique.

## Case Report

 A 46-year-old woman with no systemic history presented to the clinic due to significant mobility in tooth 2.2. The treatment plan was: extraction of tooth 2.2. After healing, soft tissue augmentation was performed using the RF technique. Two partial-thickness incisions, mesial and distal, preserving the mesial papilla, were made and connected by a crestal incision. Next, the connective tissue from the palatal fibromucose was detached. Once obtained, it was transferred to the vestibular flap. An Avinent implant (4 x 10) was placed using osseodensification drills. After implant placement, the flap was sutured, and immediate provisionalization was performed.

## Conclusions

 The RF technique allows for the correction of vestibular defects, achieving proper emergence profiles and obtaining aesthetic and predictable results.

# Abstract: Rehabilitation with implants and immediate loading in the maxillary throught guided surgery.About a case

**Authors:** I. Díaz Suárez, S. Cacciacane, A. Boquete, A. Labanca Mitre

**Affiliations:** *ESIRO UEMC*

## Introduction

 In the last decade an approach has emerged in the field of rehabilitation of edentulous patients: Guided surgery and immediate loading of implants. The objective of this case report is to outline the diagnostic, surgical and prosthetic protocol used in the treatment of the edentulous maxilla using the technique of guided surgery and immediate loading with implants.

## Case Report

 A 57-year-old woman comes to the clinic to undergo implant treatment in the upper jaw. Cone beam computed tomography (CBCT) reveals sufficient bone volume for implant placement. CBCT data is used to evaluate and develop the treatment plan. Finally, a total of eight implants are proposed. The digital tomographic image data were sent to specialized software (Implant studio, 3 Shape) for the creation of the surgical splint and the prosthetically guided provisional prosthesis. According to the guided splint, the flapless implants were inserted with precise milling. The provisional immediate fixed prosthesis was made and adjusted on the castable abutments, evaluating its occlusion.

## Conclusions

 This paper indicates that guided implant dentistry may constitute a successful treatment of edentulous maxillarypatients.

# Abstract: Inmediate implant placement, GRB and customized healing abutment in esthetic zone with labial plate dehiscence defect. A clinical case

**Authors:** M. Belsa Partal, S. Cacciacane, A. Boquete Castro

**Affiliations:** *Master Esiro UEMC*

## Introduction

 Immediate post-extraction implants allow a rapid and efficient oral rehabilitation by reducing the overall treatment time, the number of surgical interventions, and preservs the post-extraction alveolar bone.

## Case Report

 A 52-year-old man came to the clinic with the complaint, "I want to repare my broken tooth." Initially, a clinical and radiographic evaluation was performed to establish if immediate implantation was suitable in this case. After the atraumatic extraction of the tooth, a 3.6 x 12mm Klockner implant was placed, ensuring its primary stability. To optimize implant integration and ensure long-term success, guided bone regeneration (GBR) was performed.The GBR involved a connective tissue graft from the patient's palate using the envelope technique, and XenoGraft bone graft material from Straumann filling the GAP. This procedure was crucial for reconstructing the absence of labial bone plate and ensuring an adequate base for the osseointegration of the implant.Subsequently, a customized healing abutment with flow resin was used to contain and protect the biomaterial used, and also for guide the soft tissue to achieve a good emergence profile. The clinical results and postoperative check-ups showed satisfactory healing without significant complications and adequate bone regeneration according to control radiographs.

## Conclusions

 The use of immediate post-extraction implants, along with guided bone regeneration and customized healing abutment, reduces the overall treatment time and ensuring adequate aesthetics. It also proves to be an effective solution in oral rehabilitation; providing predictable results and improving the patient's quality of life.

# Abstract: Surgical approach to a case report of an odontogenic developmental cyst

**Authors:** M. Martínez Rodríguez

## Introduction

 In our day to day life, we find cysts associated with pulp necrosis and dental infections, these root cysts are the most common of the cysts in maxillary bones, according to the most widely used classification which is that established by the WHO (1992).

## Case Report

 Following a case that came to consultation for a full mouth rehabilitation with implants, a radiotranslucent mass in the first quadrant of large dimensions was discovered in the panoramic and CBCT , in the exploration a soft area was palpable in the apical area of the piece 14, the patient did not refer pain. After clinical and radiographic examination, a diagnosis of presumed dental cyst was reached. A surgical approach was performed in which a full-thickness flap was made, the destruction of bone was observed through a window generated as a result of the cyst, which was used to access the encapsulated lesion, all the purulent liquid inside was aspirated, the bag in which the liquid was encapsulated was removed, this started in the alveolus of the premolar, (indicating the origin) the damaged area was curetted and eliminated.After ensuring that the entire area was cleaned, minocin 100mg, mixed cortical cancellous granule bone graft and collagen membrane were placed.

## Conclusions

 This type of root cyst, although benign, if left untreated when small in size can continue to grow and damage other structures that generate such growth, and in some cases can become malignant. A cystectomy and regeneration of the damaged area must be carried out in order to subsequently place implants, which in our case was initially the reason for the patient's consultation.

# Abstract: Autotransplantation of an immature mandibular third molar using donor tooth replica and digital planification

**Authors:** M. Almeda, J. Pradillos

**Affiliations:** *Cirugía maxilofacial*

## Introduction

 Despite the short follow up, the good soft tissue healing of the papilla and the absence of symptoms or signs of periodontal or pulpal complications made us hope for a good prognosis. Cad-Cam and IA assisted practices in endodontics, and in other dental specialties, demonstrated to be the tool of the present that help us achieve the best results according to mimic and respect the biological needs tohealth.

## Case Report

 A 16-year-old male patient that finished orthodontic treatment a month ago was referred to evaluate the current state of the 4.6. The medical history revealed no general problems. The clinical and radiographic examination showed a previously treated first molar with coronal filtration and decay around the composite obturation.Also showed an apical root resorption in the distal root, a possible perforation in the coronal third of the mesial root because of stripping in the danger zone and a fistula associated to a chronic apical periodontitis. Mandible third molars where in stage R1/2 and R3/4 in the mesial and distal root respectively according to Moorrees et al. (8)After discussing the treatment options with the patient and his mother, it was decided to extract the firs molar and perform an autotransplantation of the immature third molar located in the same quadrant of mandible.

# Abstract: Rehabilitation with Guided Surgery in a Partially Edentulous Periodontal Patient with Immediate Aesthetic Loading

**Authors:** C. Martínez Sotillo, S. Cacciacane Entreríos, A. Boquete Castro

**Affiliations:** *Escuela Superior de Implantología (ESIRO) Universidad Europea Miguel de Cervantes*

## Introduction

 Guided surgery offers the advantage of being a more precise technique with reliable and predictable results. The use of a surgical stent increases accuracy and reduces the size of the incision. Additionally, immediate loading improves aesthetics and adequately shapes the peri-implant tissues, offering greater satisfaction and comfort to the patient.

## Case Report

 A 59-year-old male with no relevant medical history, initially diagnosed with generalized stage IV grade C periodontitis, under a periodontal maintenance program. He presented after a year of periodontal stabilization, extraction of 1.2, 1.5, and 1.6, and sinus lift in the first quadrant to rehabilitate the first quadrant by placing three implants with immediate aesthetic loading. Using the overlay of the digital diagnostic wax-up from 1.2 to 1.6 and the CBCT, the placement of three prosthetically guided implants was planned with the SimplantPro 18.5 software (Dentsply Sirona). On the day of surgery, once the surgical stent was placed, the complete Astra Tech drilling protocol (Dentsply Sirona) was carried out in 1.3, 1.5, and 1.6 flapless, achieving a torque greater than 35Nw in all of them. At the 1.3 level, a connective tissue graft from the tuberosity was performed using an envelope technique to gain volume. 2mm transepithelial abutments were screwed in, and a post-surgical CBCT was performed to verify the final position of the implants. Once the implants were osseointegrated, the final prosthesis from 1.2 to 1.6 was fabricated. After a year of loading, peri-implant health was observed, as well as satisfactory aesthetics and function.

## Conclusions

 Digitally planned implant-prosthetic rehabilitation avoids vital anatomical structures, allows for minimally invasive flaps, and helps place implants in the most aesthetic and precise way possible. Additionally, it reduces chair time and allows for immediate prosthetic loading.

# Abstract: The effectiveness of osseodensification drilling protocol on implant stability. Case report

**Authors:** C. Navarro Lama, G. Sainz De La Maza, A. Tinoco, J. De Elío, M.A. Alobera, M. Del Canto

**Affiliations:** *Universidad de León*

## Introduction

 Primary stability in dental implants is an essential factor for achieving successful osseointegration. Drilling protocols and bone quality are some of the most important factors in ensuring primary stability of implants, which is essential to ensure the long-term success of them. Many different osteotomy procedures have been proposed in the literature for dental implant site preparation. Osseodensification is considered a novel, less invasive technique that aims at bone preservation and compaction through non subtractive bone drilling. By using this protocol, we can place implants while increasing quantity and density of periimplant bone. The aim of this poster is to compare the results of both techniques, by studying the stability right after the implant placement and months later to predict the effectiveness of osseodensification drilling protocol.

## Case Report

 This case report presents the treatment of a 32-year-old female who had missing molars in the mandible. After studying the case we decided to place two implants using osseodensification technique and two implants using the conventional drilling technique, all of them in the posterior mandible ridge.

# Abstract: Oral Rehabilitation with Vertical Dimension Increase A Case Report

**Authors:** J. Martínez Urrea, M. Prieto Álvarez, J.C. Salazar Antezán, A. Bowen Antolín

**Affiliations:** *Master's Program in Implantology and Periodontology, Universidad Isabel*

## Introduction

 The loss of vertical dimension (VD) affects a significant number of middle-aged and elderly patients, with a similar incidence in both men and women.

## Case Report

 In our case, we treated a 73-year-old male who exhibited a clear loss of vertical dimension, fractured implants in positions 15 and 16, a reconstructed upper anterior sector, and severe generalized wear. Additionally, he experienced significant and continuous muscular pain. We classified the patient, according to his biological system, as DECOMPENSATED and in an ACUTE PATHOLOGICAL state. The objective was to restore the patient's functionality, health, and aesthetics through a comprehensive treatment that included both a surgical phase and a rehabilitative phase. During the surgical phase, we had to explant the fractured implants, and in the same surgical procedure, we placed implants in positions 14 and 17. We regenerated with autologous dentin from the patient's extracted teeth (in this case, tooth 18), placed a resorbable collagen membrane, and A-PRF. Five months later, we placed another implant in position 15. The rehabilitative phase involved the use of advanced technology at the beginning and end of the treatment. EMG allowed us to estimate the calculation of the patient's lost VD. Based on this, we created an orthotic splint to verify tolerance to the increase in VD. We also conducted a DSD and Mock-up study to preview the final treatment outcome, thus achieving an aesthetically guided increase in VD. The results indicated a significant improvement in occlusal function, health, and aesthetics, demonstrating the effectiveness of this integrated approach.

## Conclusions

 Implantology is a key factor in the recovery of strategic lost teeth to ensure occlusal stability. For the treatment of these patients, a multidisciplinary approach is indispensable, the use of digital resources, and being guided by EMG, which provides predictability and reliability throughout the treatment.

# Abstract: Biconometric prosthesis on implants. A Case Report

**Authors:** K. Ruiz Lopez, C. Zarza Diestro, J. Herrera, A. Bowen Antolín

**Affiliations:** *Master's Program in Implantology and Periodontology, Universidad Isabel I*

## Introduction

 The main distinguishing feature of biconometric prostheses lies in their fixation system, using a friction mechanism. This system eliminates the need for additional elements, simplifying the placement process and reducing the risk of complications.

## Case Report

 The clinical case presents a 60-year-old male, with no significant history, who is rehabilitated on four implants with the placement of a biconometric prosthesis of immediate loading of the pieces 15 to 24.The planning was carried out using the 2 INGIS application, the surgery was performed using an open surgical guide and the immediate loading prosthesis was made of PMMA. The final prosthesis was made of Zirconia and the procedure was simple and effective.

## Conclusions

 The process followed reveals that biconometric prostheses can be a simple, natural and effective solution for the implantological process. The planning process and the prosthetic phase have specific characteristics that must be trained for the effectiveness of the treatment.

# Abstract: Immediate Implant Simultaneous with Bone and Connective Tissue Regeneration: A Case Report

**Authors:** C. Costa Lovera, S. Cacciacane, A. Boquete Castro

**Affiliations:** *Master Esiro UEMC*

## Introduction

 Careful planning is essential for the success of dental implants. Evaluating the patient's conditions with advanced techniques like CBCT ensures the correct selection of the implant and the need for additional procedures such as guided bone regeneration (GBR). Considering aesthetic and functional aspects, as well as the patient's habits and medical history, helps prevent complications and maintain appearance and function during healing.

## Case Report

 Clinical case of a 42-year-old male patient, classified as facial Class III, who smokes five cigarettes a day, with no history of allergies or significant systemic diseases. The patient requires the extraction of tooth 2.4 as it is non-restorable. Additionally, he presents with caries in teeth 3.6 and 4.6 and lacks lateral and anterior occlusal guides. The proposed treatment plan includes the restoration of teeth 3.6 and 4.6 with fillings and the extraction of tooth 2.4. Subsequently, an immediate implant placement in site 2.4 is planned. To optimize the implant outcome, guided bone regeneration (GBR) and a connective tissue graft (CTG) are planned. Additionally, an immediate provisional prosthesis will be placed in site 2.4 to maintain aesthetics and function during the healing period. During the preoperative evaluation, the suitability of the post-extraction bone for implant placement was determined. The CBCT study showed a bone width of 9.76 mm and a height of 21.1 mm, which is adequate for the planned implant insertion. A Dentium brand implant, measuring 4x12 mm, was chosen to ensure optimal primary stability and adequate integration with the alveolar bone.

## Conclusions

 This case highlights the importance of meticulous planning and a multidisciplinary approach for comprehensive patient treatment. The combination of advanced regeneration techniques and the use of immediate prostheses allows for successful functional and aesthetic rehabilitation, improving the patient's quality of life.

# Abstract: Transcrestal Sinus Lift with Osteotomes and Biological Drilling Combined with L-PRF. Clinical Result

**Authors:** A. España Guerrero, E. España Guerrero, J. España Guerrero, E. Velasco Ortega, G. Moreu Burgos, A. España López

**Affiliations:** *Máster de Implantología Oral. Universidad de Granada. Granada*

## Introduction

 The transcrestal sinus lift is a conservative technique that allows for the elevation of the maxillary floor using osteotomes, facilitating implant placement in patients with moderate to severe atrophy in the posterior maxilla. Implant insertion can be challenging due to the reduced height of the maxillary alveolar ridge combined with increased sinus pneumatization. Maxillary sinus lift techniques provide a bed for implant placement by modifying the residual ridge, inserting autologous bone or bone substitutes, or a combination of both between the residual maxillary ridge and the sinus mucosa.

## Case Report

 A 64-year-old patient with no significant medical history. Clinical examination revealed a single-tooth edentulism in space 27. Treatment planning included an atraumatic sinus lift. Biological drilling was performed to obtain high-quality autologous bone combined with PRF, a simple method developed to prepare fibrin gels without exogenously added supplements. Simultaneous placement of a Galimplant® implant and rehabilitation with a screw-retained prosthesis were carried out. The follow-up period was 10 years

## Conclusions

 The survival rate of implants placed using this transcrestal technique is over 90%, making it a predictable technique for dental implant placement when following the appropriate protocols.

# Abstract: Soft Tissue Management in Immediate Post-Extraction Implant. Palatal Rotation Flap

**Authors:** E. España Guerrero, A. España Guerrero, J. España Guerrero, E. Velasco Ortega, G. Moreu Burgos, A. España López

**Affiliations:** *Máster de Implantología Oral. Universidad de Granada. Granada*

## Introduction

 The placement of immediate post-extraction implants has shown significant evolution. The presence of a thick gingival biotype is important to minimize aesthetic risk. In its absence, several techniques exist to modify this gingival biotype, including subepithelial connective tissue grafts, free gingival grafts, or pedicle flaps, which also facilitate primary closure of the socket.

## Case Report

 An older, non-smoking patient underwent the placement of a 3.5x14 mm IPX Galimplant® immediate post-extraction implant after a thorough evaluation. The buccal wall had a significant defect. A palatal rotation flap was performed to increase the keratinized mucosa and ensure primary closure of the post-extraction socket simultaneously. The final restoration was completed after 3 months. A key advantage of immediate implants is that bone healing occurs simultaneously with osseointegration, reducing healing time. The palatal rotation flap is a useful and quick procedure for immediate post-extraction implants, facilitating complete, precise, and highly predictable coverage of the implant bed, even in cases of large defects requiring regenerative therapies and multiple implants. Although not essential for implant survival, a band of keratinized mucosa greater than two millimeters aids in maintenance and hygiene, preventing recessions.

## Conclusions

 Implant rehabilitation using the immediate post-extraction implant technique and closure with a palatal rotation flap is a highly predictable clinical procedure. The palatal rotation flap is a predictable technique to increase the keratinized mucosa around implants and facilitate primary closure of the socket.

# Abstract: Crestal sinus elevation using the osseodensification technique. What is the limit?

**Authors:** T. Albi Escolante, G. Herrera Márquez, V.O. Ruiz Olivares, A. Bowen Antolín

**Affiliations:** *Master's Program in Implantology and Periodontology, Universidad Isabel I*

## Introduction

 When we try to restore function in the posterior region of the maxilla, we mainly encounter two major barriers: the availability of bone in height due to the presence of the maxillary sinus and bone density. Dentistry in recent years has been evolving, changing the tendency when it comes to carrying out treatments, advocating respect for biology, tissues and being minimally invasive. The treatment of choice to perform a sinus lift with less than 4 mm of residual bone has traditionally been the lateral or open technique, however, technological advances allow us to perform the closed technique or crestal sinus lift in cases where the patient has 2-3 mm of residual bone with simultaneous implant placement. At the same time, the primary stability of the implants placed in this situation and the preservation of the existing tissue is considerably improved if we use the osseodensification technique.

## Case Report

 Two clinical cases are presented, a 40 year old male and a 56 year old male, that came to the clinic with the purpose of implant rehabilitation, both with less than 4 mm of bone height. In both cases, the Densah drills were used for sinus elevation. Continuous revisions were carried out, in one of the cases, a tomography was shown after 3 years of loading

## Conclusions

 Some studies have shown that crestal sinus lift with or without osedensification is viable for implant placement when the residual alveolar bone height is less than 4 mm, but more research is still needed to be able to affirm this premise.

# Abstract: Periodontal regeneration with enamel matrix-derived proteins (EMD) and xenograft in patients undergoing orthodontic treatment and monoclonal antibody treatment

**Authors:** M. Arabi Tihlili, A. Gonzalez Ruiz, A. Boquete Castro, S. Cacciacane, S. Udina Silvestre, Y. Garcia Sanjuan

**Affiliations:** *Máster Internacional Implantología y Rehabilitación Oral. Escuela superior de Implantología y Rehabilitación Oral.Universidad Europea Miguel de Cervantes*

## Introduction

 The tissues of the oral cavity are closely interrelated. The success of orthodontic treatment is going to depend on the integrity and health of periodontal tissues that must be kept healthy before, during and after orthodontic treatment (OT). In adult patients with periodontitis, OT is a challenge, due to the biomechanical conditions imposed by age and the state of periodontal involvement. Therefore, the maintenance of good oral hygiene and periodontal control throughout the periodontal treatment is a key point to avoid bone resorption and periodontal pocket formation. The aim of this work is to present a case of a 62-year-old female patient in TO, with stage II grade B periodontitis and great bone loss in 31,41 and 42 treated with periodontal regeneration surgery with enamel matrix-derived proteins (Emdogain®, Straumann) and xenograft.

## Case Report

 Woman with grade II mobility, pain and suppuration during TO at the anteroinferior level. She refers to having been treated with 4 biannual injections of monoclonal antibodies (PROLIA 60 mg), so she waited 9 months to perform surgery. To address the case, periodontal regeneration surgery of the intraosseous and interdental defect was performed using enamel matrix-derived proteins and xenograft. The minimally invasive surgical technique (M-MIST) and the modified papilla preservation technique (MPPT) were used. In the radiographic control there is bone neoformation around the affected teeth together with decreased mobility and total absence of suppuration.

## Conclusions

 Treatment of advanced periodontitis at localized sites using xenograft and enamel-derived proteins is effective. Treatment results appear to improve the condition of evolving periodontal tissues over the years.

# Abstract: Autogenous dentin as graft material in alveolar preservation

**Authors:** M. Costa Castillo, V. Schiavo Di Flaviano, J. López-López

**Affiliations:** *Máster de Medicina, Cirugía e Implantología Oral. Universidad de Barcelona Campus Bellvitge, C/ Feixa Llarga, s/n, 08907 L'Hospitalet de Llobregat, Barcelona*

## Introduction

 Randomized clinical trials and systematic reviews have suggested that autologous bone grafts harvested from permanent teeth can be an effective alternative material for alveolar preservation. Ten percent of the dentin matrix is composed of non-collagenous proteins involved in bone calcification and growth factors, providing teeth with osteoinductive properties.

## Case Report

 A 70-year-old male patient with no known allergies and a medical history of controlled Type 2 Diabetes, who quit smoking 30 years ago, presented for evaluation of upper tooth mobility. Clinical and radiological examination revealed advanced periodontal disease, teeth with grade 3 mobility, and implants in positions 1.6, 1.7, 2.6, and 2.7 with peri-implant issues. Initial prophylaxis was performed, followed by the explantation of implants in positions 1.6 and 1.7, and treatment of peri-implantitis in implants in positions 2.6 and 2.7. Subsequently, the upper teeth were extracted and processed for use as regenerative material in alveolar preservation. This process included cleaning and drying each tooth, followed by placement in a grinding chamber for three cycles of 20-second each. The teeth were then disinfected with sodium hydroxide and ethanol for 12 minutes, followed by two cycles of saline solution cleaning for 2-3 minutes each. Excess solution was removed with a pipette and gauze, and alveolar preservation was conducted. The surgery was completed with the placement of collagen sponges over each alveolus and cross-stitch sutures. After a four-month waiting period, a CBCT scan of the upper arch was performed to evaluate implants. Alveolar preservation successfully maintained the maxillary bone height, and four implants were placed to support an overdenture on a bar.

## Conclusions

 Based on our results, autogenous dentin may be an effective alternative biomaterial for alveolar preservation. However, further studies are needed to assess long-term stability.

# Abstract: Digital planning of subperiosteal implants and zygomatic implants: a clinical case

**Authors:** K. Ivaylova, D. Sahli, P. Maguregui, M. Castro, S. De La Vega, J. López-Quiles

**Affiliations:** *Postgrado de Especialización en Cirugía Bucal e Implantología, Facultad de Odontología, Universidad Complutense de Madrid*

## Introduction

 Subperiosteal implants are a treatment option for atrophic jaws in medically compromised patients, patients with sinus pathology and treat large maxillary defects. On the other hand, zygomatic implants are a valid option for the total rehabilitation of arches with aggravated alveolar resorption.

## Case Report

 A 75-year-old woman came to the master's clinic because "she wanted to replace the absent tooth". During the examination, the absence of 35, 36, 45 and 46 was observed. Subperiosteal implants were planned to be placed in positions 3.5, 3.6, 4.5 and 4.6. In the upper arch, two zygomatic implants were placed on each side and a single implant in the 2.1. position. For this purpose, the CBCT is sent to the company in charge of manufacturing the structure of the subperiosteal implants. The structure is then designed and validated, after which it is sent for 3D printing and shipment. After the detachment, the ostectomy guide splint is placed to make perforations with the 2.0 drill and place the anchorpins for stabilization. After the ostectomy, the structure is fixed with self-tapping screws and the soft tissue repositioning and suturing is performed. In the upper arch, incision and debonding is performed on both sides of the arch and the two zygomatic implants are placed on each side of the arch. In addition, a traditional implant is placed in position 2.1. to improve the stability of the prosthetic structure.

## Conclusions

 Subperiosteal implants are a valid option for the treatment of patients with large alveolar resorptions. Currently, digital planning is essential to be able to carry out this treatment. In upper jaw, when the resorption is substantial, it is necessary to think in other options like zygomatic implants that can provide the needed stability for such cases.

# Abstract: Step-by-Step for Minimally Invasive Surgery: Immediate Loading

**Authors:** D. Sahli, A. Garafulic, P. Maguregui Ortiz, P. Revuelta Cortés, C. Madrigal, M. Castro Janeiro

**Affiliations:** *Department of Specialization in Oral Surgery and Implantology, Faculty of Dentistry, Complutense University of Madrid*

## Introduction

 Guided dental implant surgery is increasing in popularity nowadays, particularly due to the advances in, and increased usage of, Cone Beam Computed Tomography (CBCT) and the development of dental implant treatment planning software that allows for a three dimensional assessment of the implant site. Preoperative planning of the implant position, as part of a comprehensive prosthetic and surgical approach, is becoming increasingly important regarding function and esthetics.

## Case Report

 A 59-year-old woman sought consultation for upper arch rehabilitation. She presented with missing teeth at positions 14, 15, 16, and 25, along with generalized root resorption following five years of orthodontic treatment. The treatment plan involved complete fixed implant-supported rehabilitation using guided surgery and immediate loading with the Medicalfit protocol. This included extraction of remaining teeth and placement of implants at positions 16, 14, 12, 22, 25, and 26. Subsequently, a CBCT scan and digital wax-up were performed and a surgical guide was fabricated. Implants were placed using guided surgery, and post-surgical records were taken to generate an STL file for the provisional PMMA prosthesis fabrication.The prosthesis was placed shortly after surgery, effectively restoring both function and aesthetics, and the patient reported increased satisfaction.

## Conclusions

 Immediate functional loading of full-arch implant-supported prostheses represents a predictable solution for the rehabilitation of edentulous patients. This protocol maximizes the success of guided surgery techniques, offering numerous benefits such as shorter treatment times and increased patient comfort.

# Abstract: Double full-arch implant-supported fixed dental prostheses. A case report

**Authors:** N. Torres Velázquez, P. Roca Obis, P. De Marcos Sabata, A. García Pérez, J. López-López

**Affiliations:** *Master of Medicine, Surgery and Implantology - University of Barcelona – Faculty of Medicine and Health Sciences [Dentistry]. C/ Feixa Larga s/n. Hospitalet de Llobregat, Barcelona, 00907 *

## Introduction

 With wide spread use of dental implants for the restoration of lost dentition, rehabilitation of completely edentulous arches with dental implants has become quite common. Full arch rehabilitation can be done either by fixed prosthesis or removable overdenture prosthesis. All on 6 implant procedure is a widely followed method to restore fully edentulous dental arches. Full arch implant rehabilitation requires correct case selection, astute surgical procedure, and careful prosthetic planning for a successful outcome.

## Case Report

 A 46-year-old man, with an allergy to penicillin and a medical history of hypertension and hyperthyroidism, with partial upper and lower edentulousness, comes for consultation because he wants to rehabilitate both arches with a fixed prosthesis. With advanced chronic periodontitis and generalized type II and III mobility, extraction of all remaining teeth is proposed to perform alveolar preservation using the Smart Dentin Grinder. After three months, re-entry is performed, and six upper and lower implants are placed for subsequent fixed upper and lower rehabilitation with metal-ceramic prosthesis. The resin prosthesis is maintained for 6 months and after this time the definitive metal-ceramic prosthesis is made.

## Conclusions

 Fixed implant-supported prosthesis is a good option for patients for rehabilitation of edentulous arches. The successful treatment outcome requires correct diagnosis and accurate implant planning.

# Abstract: Sinus occupation pre implant placement, clinical case report

**Authors:** V. Robles Oneto, A. Torrejon Moya, M. Costa Castillo, V. Schiavo Di Flaviano, C. Arranz Obispo, J. López-López

**Affiliations:** *University of Barcelona Dental Hospital, Bellvitge, Barcelona*

## Introduction

 Maxillary sinus pathology is common, studies have reported that 5% to 38% are caused by odontogenic reasons. Sinus lift augmentation procedure has been gaining more acceptance among dental professionals, because of its successful rate (98,3%) to elevate the bone availability in posterior maxillary edentulous areas before rehabilitating with dental implants. Therefore, presurgical evaluation with cone beam computed tomography (CBCT) has become an essential tool for diagnosis, surgical planning, and the study of sinus anatomy. Before performing a sinus lift, the attention should be on anatomical features, like the presence and/or patency of the ostium, the thickness of cortical bone and Schneiderian membrane, and the possibility of finding sinus pathology which needs to be previously treated.

## Case Report

 A 48-year-old female patient, with no relevant medical history, consulted for rehabilitation with dental implants. An edentulous area of the first quadrant was observed with poor bone availability, therefore sinus elevation is indicated prior to implant placement. In the CBCT, occupied maxillary sinuses were observed, so it was decided to be treated with oral antibiotics and antihistamines, plus nasal corticosteroids for 7 days. In the re-evaluation, unoccupied maxillary sinuses were observed, and surgery was planned.

## Conclusions

 Dentists contemplating sinus augmentation should know how to diagnose and treat sinus pathology, whether related or unrelated to the teeth. In addition to creating potential liability, lack of knowledge may also affect patient treatment, leading to over or undertreatment.

# Abstract: Peri-implant connective tissue augmentation using the Omega Roll Envelope Flap Technique: A case report

**Authors:** F. Arisa Petitbò, A. Torrejon Moya, E. Jané Salas, A. Estrugo Devesa, A. Mari Roig, J. López-López

**Affiliations:** *Màster de Medicina,Cirurgia i Implantologia Oral Universitat de Barcelona (UB)*

## Introduction

 Procedures based on flap rotation or grafting from another donor area stand out in the treatment of soft tissue deficiencies. Many techniques have been described to increase the thickness of the peri-implant soft tissue, among them the Omega Roll Envelope Flap Technique. This modification of the classic rotating pedicle flap of the palate was described by Dr. Pandolfi in 2018 and allows to increase and optimize the soft tissue avoiding obtaining autologous connective tissue from another donor area. Current evidence suggests a minimum 2 mm thickness of keratinized mucosa perimplantaria for long-term favorable and stable results. Correction of the defect was evidenced by improving aesthetics in a minimally invasive manner, reducing invasiveness and morbidity.

## Case Report

 A 45-year-old male patient presents with no medical history of interest or known allergies. Clinical and radiological examination showed the absence of 1.3 and lower posterior sectors. When evaluating the case, Biohorizons TRX 3.8x12mm implant placement was performed. In the second surgical phase, a volume defect of the periimplant soft tissue was observed. It was decided to perform the "Omega Roll Envelope Flap" technique to increase the thickness of peri-implant soft tissue, thus improving the long-term survival of the implant, as well as aesthetics. The follow-up at 7, 15, and 21 days showed correct healing so it was decided to start the definitive prosthetic rehabilitation.

## Conclusions

 The use of the "Omega Roll Envelope Flap" technique has efficiently demonstrated advantages in increasing the thickness of peri-implant soft tissue as it maximizes and optimizes the amount of connective tissue that can be found inside the vestibular flap.

# Abstract: Block bone grafts for horizontal dimension augmentation in relation to two clinical cases

**Authors:** A. Bauer González, A.F. Stran Lo Giudice, J. Bartolomé Lechuga, R. Serrano Zamora, L. Diaz Olivares

**Affiliations:** *Universidad Complutense de Madrid*

## Introduction

 Tooth loss results in horizontal and vertical resorption of the alveolar ridge, leading to a reduction in the volume of alveolar bone, which can complicate subsequent implant rehabilitation. To address this issue, various bone regeneration techniques are available, including block bone grafts, guided bone regeneration, and alveolar distraction osteogenesis. Block bone grafts are a technique that enables the recovery of bone dimensions both vertically and horizontally, yielding predictable and stable long-term results. These grafts can be autologous, heterologous, or allogeneic, with autologous bone currently considered the gold standard due to its osteogenic, osteoinductive, and osteoconductive properties. The objective of this study is to describe and compare the bone regeneration technique using intraoral block grafts for subsequent rehabilitation with dental implants, supported by the description of clinical cases.

## Case Report

 A 53-year-old female patient, classified as ASA I, presented to the Oral Surgery and Implantology Department at the Complutense University of Madrid, seeking to replace teeth 11 and 21 with fixed implant-supported prostheses. Clinical examination revealed a deficiency of horizontal bone, which was subsequently confirmed by CBCT imaging. It was decided to perform a chin block graft to address the horizontal bone deficiency for the subsequent placement of two implants after the osteogenesis period.

## Conclusions

 Intraoral block grafts are a predictable technique for horizontal bone augmentation, with similar outcomes in terms of bone gain, resorption, and implant survival. However, higher complication rates are observed with chin grafts, necessitating individualization of each case.

# Abstract: Surgical technique with quad Zygoma in an atrophic maxilla: a case report

**Authors:** L. Campo Fernández, L.A. Chauca, M.D. Reboiras López, E. Otero Rey, A. Blanco Carrión

**Affiliations:** *Máster Medicina Oral, Cirugía Oral e Implantología de la Universidad de Santiago de Compostela*

## Introduction

 At present there are predictable and reproducible surgical techniques for treatments in patients with maxillary rim atrophy, zygomatic implants are one of the alternatives. The interdisciplinary work between the oral rehabilitator and the maxillofacial surgeon is fundamental for the success of the patient's rehabilitation.

## Case Report

 The present clinical case reports a 60 year old female patient, with no medical history, where atrophy of the alveolar ridge was observed in the conebean. Therefore, four zygomatic implants were planned with the purpose of a hybrid rehabilitation. The study was done virtually making guide for 3D surgery. The surgical procedure was performed under general anesthesia and with intraoral infiltration of local anesthetic. An incision is made in the paracrestal area, performing distal discharges towards vestibular. Lifting is carried out mucoperiosteal flap and bone exposure of maxillary alveolar ridge, anterior wall of sinus maxilla, emergence of infraorbital nerve and butress maxillary zygomatic to zygomatic arch. Surgical guide is placed and fixed with 2.0 x 9mm screws, after virtual planning, where it was decided to perform extra sinus reaming. Milling started with a round bur to create a guide. Then, we use the initial lancet bur. A sequence of milled from the 2.8mm diameter milling cutter ensuring penetration of the zygomatic bone, continuing with the bur 3.2mm and finish with the 3.6mm drill. Finally, the implants were placed at 25 rpm and with a torque of 30N. The flaps were repositioned and sutured. In addition, a complete prosthesis was adapted.

## Conclusions

 One of the treatment alternatives in maxillae with atrophic rims is the placement of zygomatic implants together with a good digital planning. Several studies have described that the success rate of these implants is very high, due to the fact that the technique is safe, reproducible and generates high patient satisfaction.

# Abstract: Single Implant in Second Premolar: Minimum Requirements for Bone and Prosthetic Space

**Authors:** M. Mesquida Alcover, M. Blazquez Hinarejos, A. Torrejón Moya, V. Schiavo Di Flaviano, N. Torres Velázquez, J. López-López

**Affiliations:** *Master's Degree in Medicine, Surgery, and Oral Implantology – University of Barcelona*

## Introduction

 The planning and placement of single implants require precise considerations regarding the minimum dimensions of bone and prosthetic space to ensure successful and long-lasting results. Several studies have established evidence-based guidelines for these measures. The minimum measures for the placement of a single implant include a buccal and palatal bone thickness of at least 1 mm, with 2 mm being ideal for the preservation of marginal bone height. It is recommended that the distance from the implant thread peak to the outer edge of the buccal bone be at least 0.75mm. For premolars, a mesio-distal prosthetic space of 4 to 4.6mm between the implant and the adjacent teeth is suggested. The minimum bone thickness required in the buccal-palatal direction should be 1.6 mm for upper premolars and 2.6 mm for lower molars, with an ideal thickness of 2 mm to prevent bone resorption and ensure implant stability.

## Case Report

 A 42-year-old male patient presents to the clinic with a fractured 2.5. The patient has no allergies or relevant medical history. Additionally, he has no toxic habits. The patient has a bone height of 9.6 mm and a width of 7.50mm. Therefore, it is decided to place a Biohorizons internal hexagonal connection implant of 3.8 x 9mm. Autologous bone was placed in contact with the implant along with xenograft, secured with a membrane on the buccal side.

## Conclusions

 Highlighting the importance of adhering to these minimum measures for the long-term success of dental implants. Although specific evaluation should be done on a case-by-case basis, considering the anatomical conditions and bone quality of each patient.

# Abstract: Dental autotransplantation: Case report

**Authors:** R. Ezzeddine Doughan, F. Garcia, C. Arasanz Àvila, A. Torrejon Moya, S. Egido Moreno, J. López-López

**Affiliations:** *Master of Medicine, Surgery and Implantology - University of Barcelona – Faculty of Medicine and Health Sciences [Dentistry]. C/ Feixa Larga s/n. Hospitalet de Llobregat, Barcelona, 00907*

## Introduction

 Tooth loss is an adverse consequence of oral diseases and traumatic dental injuries. Among the solutions to tooth loss are removable prostheses, dental implants, orthodontic closure and autotransplantation. Dental autotransplantation consists of the extraction of a tooth from its original position and its subsequent placement in a different position in the dental arch of the same patient.

## Case Report

 We present an 18-year-old woman, with no relevant medical history or known allergies, who visits the clinic referred for extraction of the 3.7 tooth. After a clinical and radiological examination, an autotransplant is planned, using the third molar. To perform dental autotransplantation, donor teeth that are erupted, impacted or partially impacted can be used, regardless of the degree of root formation. This may serve as a promising treatment alternative in cases of tooth loss not only in children and adolescents but also in adult patients. An autotransplanted tooth, unlike an osseointegrated implant, preserves the periodontal ligament, preserves the adjacent bone and, therefore, has greater resistance to occlusal loads. Thanks to digital planning we can select the most suitable donor tooth according to the morphology of the socket, and know the ideal 3D position and the dimensions required in the socket during surgery. On the other hand, the use of replicas helps to reduce the extraoral time of the donor tooth and its possible injury during the adjustment to the recipient's alveolus, thus increasing the success of the treatment.

## Conclusions

 Autotransplantation is a surgical technique with a high success and survival rate that can serve as an alternative to replacing a lost tooth. Proper planning and careful execution of the procedure are important to achieve optimal long-term results.

# Abstract: Importance of non-surgical treatment in the management of peri-implantitis: A case report

**Authors:** P. Suarez Perez, C. Jorva García de Casasola, B. González Navarro, V. Schiavo, A. Torrejon Moya, J. López-López

**Affiliations:** *Faculty of Medicine, Health Sciences, Dentistry, University of Barcelona. Master of Oral Medicine, Surgery and Implantology*

## Introduction

 Peri-implantitis is defined as a pathological condition that occurs in the tissues surrounding dental implants and is characterized by inflammation in the peri-implant connective tissue and progressive loss of supporting bone. It is a destructive process initiated by biofilm and mediated by the host that is influenced by modifiable and non-modifiable local, systemic, and environmental factors. For its treatment, it is recommended to start with a non-surgical approach and after a re-evaluation, consider surgical treatment.

## Case Report

 A 66-year-old female patient with no medical history, allergies, or toxic habits came to the clinic due to discomfort in the area of the 4.6 implant placed in 2016. After the first year of maintenance, the patient did not attend subsequent visits. Intraoral examination showed a probing depth of 4 mm on the entire lingual surface and a depth of 5 mm and 7 mm on the mesiobuccal and midbuccal surfaces respectively, with bleeding on probing throughout its circumference. The periapical radiograph showed a greater loss of bone support on the mesial surface of the implant. Non-surgical treatment was performed after unscrewing the prosthesis using instrumentation with teflon curettes and irrigation with 10% H2O2 and 0.12% Chlorhexidine. During follow-up and at the last maintenance at 9 months, an improvement in clinical parameters was observed, as well as recovery of marginal bone and greater radiopacity of the bone defect.

## Conclusions

 Non-surgical treatment is always considered the first step in the management of peri-implantitis. Although it has certain limitations, especially in advanced cases, it has been shown that instrumentation together with chemical decontamination of the implant can be successful in mild or moderate cases, highlighting the importance of early diagnosis.

# Abstract: Surgical peri-implant treatment with implantoplasty: A clinical case

**Authors:** E. Arcas, C. Arasanz, J. Luengo, A. Torrejón, S. Sardañés, J. López

**Affiliations:** *Department of Odontostomatology, Faculty of Medicine and Health Sciences (Dentistry). Bellvitge Campus, University of Barcelona, Barcelona Spain. Pavelló de Govern, 2nd floor, C/Feixa Llarga, s/n, 0897 L'Hospitalet de Llobregat, Barcelona*

## Introduction

 Resective surgery on implants is a surgical treatment option for the treatment of peri-implantitis to decontaminate the surface of the implant, creating an anatomy of the peri-implant hard and soft tissue that allows correct oral hygiene. The combination of ostectomy and osteoplasty with implantoplasty represents an effective therapy to reduce or maintain peri-implantitis.

## Case Report

 A 69-year-old woman with chronic bronchitis and wearing a fixed prosthesis on 6 implants attends the master's degree in Oral Medicine, Surgery, and Implantology to evaluate the implant in position 1.4. A fistula and suppuration and radiographic bone loss of 50% the implant was observed. Mucoperiosteal flap elevation was performed and granulation tissue was removed until healthy surrounding bone was observed. A thin layer of bone was observed on the buccal wall of 1.4. Therefore, implantoplasty was performed eliminating the threads of the implant, with a tungsten carbide bur and removing the titanium particles that remain adhered to the soft tissue. Subsequently, primary closure was performed with non-absorbable suture. Proper healing of the soft tissues was observed, exposure of the implant 1.4 and good access for oral hygiene.

## Conclusions

 Resective surgery is a therapeutic option that allows stabilization of peri-implant disease. Implantoplasty is a complementary technique in that should be used in specific cases. It is not advisable to perform it on narrow implants because it can reduce the biomechanics of the implant or in aesthetic areas due to soft tissue recession.

# Abstract: Implants in esthetic zones: bone regeneration techniques - a case study

**Authors:** L. Maftei Rusu, M. Costa Castillo, J. Parra Moreno, A. Torrejon Moya, V. Schiavo di Flaviano, J. López-López

**Affiliations:** *Faculty of Medicine and Health Sciences (Dentistry). University of Barcelona (UB). Master of Oral Medicine, Surgery and Implantology (MCIO). University of Barcelona Dental Hospital*

## Introduction

 Dental implants constitute an optimal treatment choice for the rehabilitation of edentulous areas in anterior regions. These treatments pose a true challenge, as they must restore not only the functionality of the stomatognathic system but also the esthetics of the tissues. Bone availability is a fundamental requirement for the safe placement and achievement of a competent esthetic result. It has been reported that up to 50% of bone volume is lost following tooth extraction within the first year. This physiological resorption significantly affects bone availability for dental implant placement, making it common to encounter clinical situations where a bone regeneration procedure is indicated.

## Case Report

 A 64-year-old female patient presented to the Master's program in Medicine, Surgery, and Oral Implantology at the Hospital Odontològic Universitat de Barcelona for the rehabilitation of tooth 2.3 with a reduced prognosis, without the possibility of restoration, and with a vestibulo-distal bone defect. It was decided to perform a simultaneous bone regeneration technique during the implant placement (tapered internal implant 3.0x10.5mm Biohorizons®) using bovine xenograft (Cerabone®) and a collagen membrane (Collprotect® membrane).

## Conclusions

 There are multiple factors that can influence the choice of one type of regenerative procedure over another, such as the primary stability of the implant, the adequate three-dimensional position of the implant, the location of the bone defect in the esthetic zone, the remaining bone volume, the type of bone defect, and the amount of space to be restored. In fixed rehabilitations using implants in esthetic zones, the correct three-dimensional placement of an osseointegrated implant, accompanied by an adequate volume of soft and/or hard tissues, is essential.

# Abstract: Immediate Implant Placement Vs. Early Implant Treatment in the Esthetic Area: A Case Report

**Authors:** N. El Ouaghmiri, M. Costa Castillo, B. Tejedor Coll, R. Aceves Arguemí, J. López-López

**Affiliations:** *Master of Medicine, Surgery and Oral Implantology. Faculty of Medicine and Health Sciences (School of Dentistry). University of Barcelona*

## Introduction

 In the last two decades, the early and immediate placement of dental implants has sparked vigorous debate within the field of implantology. While some argue that immediate placement reduces interventions and treatment duration with reduced invasiveness, enhancing aesthetics by preserving soft tissue morphology, others voice concerns about potential complications like gingival recession, long-term tissue stability, and implant survival rates. Consequently, they advocate for early placement to ensure predictable aesthetic outcomes and enhanced tissue stability, assessed through the Pink Aesthetic Score (PES), thereby achieving a harmonious blend of treatment efficiency and safety in implantology.

## Case Report

 The case of a 65-year-old male patient with benign prostatic hyperplasia treated with tamsulosin and without known allergies is presented. He comes to the clinic for fixed rehabilitation on teeth 1.4, 3.5, 4.6 and 4.7. Immediate placement of the dental implant at the site of tooth 1.4 was initially planned, but due to an active fistula and loss of the buccal table, the intervention was postponed for 1.5 months. The procedure included extraction of tooth 1.4, preparation of the bone bed, and insertion of implant 15 measuring 3.8 x 10.5 mm, with Cerabone® 0.5 bone graft covered by Collprotect membrane® 15x20 resorbable collagen membrane for protection and regeneration, secured with thumbtacks. Postoperative care and close follow-up were scheduled to ensure treatment success.

## Conclusions

 The immediate and early placement of dental implants offers advantages in tissue conservation and aesthetics. However, immediate placement may carry risks of gingival recession and peri-implantitis, and although significant failures are not observed in the first year, careful patient selection and surgeon experience are crucial. In comparison, early placement ensures better aesthetic results and lower risk of complications, highlighting the importance of a thorough prior evaluation for successful treatment.

# Abstract: Short Monophasic Implants in Severe Vertical Atrophy of the Mandible, a Case Report

**Authors:** R. Prat Riera, C. Arasanz Àvila, E. Arcas Castellà, A. Roselló Camps, C. Polis Yanés, J. López-López

**Affiliations:** *Facultad de Medicina y Ciencias de la Salud [Odontología] - Máster Medicina, Cirugía e Implantología Oral - Universitat de Barcelona*

## Introduction

 Oral implantology has undergone continuous evolution since the introduction of dental implants. Numerous improvements in various aspects of implants have been observed. However, two main designs have remained clearly distinct: two-piece implants, introduced and developed by Brånemark, and one-piece implants by Schroeder. Monobloc or monophasic implants are defined as implants in which the intraosseous portion, the transepithelial portion and the prosthetic abutment are manufactured as a single unit with no discontinuity between them. Although scientific evidence is limited, the survival rate of monophasic implants in the literature is 96.79% in 5 years of follow-up. They involve rapid functional rehabilitation with reduced operating time, little involvement of the surrounding tissues and less morbidity for the patient, with no gap between implant and connection. Mechanically, it avoids screw loss and attachment fracture. However, it involves a limited prosthetic range. As the literature indicates, survival is significantly higher with short implants compared to regular implants in augmented bone over a short observation period of up to 1 year.

## Case Report

 A 71-year-old female patient allergic to penicillin, cephalosporin and erythromycin, with no medical history of interest, comes to the surgery department to assess a fixed rehabilitation of the lower arch. He presented a vertical bone defect in the posterior sector with an interoculsal space of 12mm and 13mm in the third and fourth quadrants, respectively. We opted for the placement of short monophasic implants in both quadrants, with immediate provisionalisation for the patient's rehabilitation, obtaining an immediate result and involving less morbidity than a regenerative process.

## Conclusions

 Short monophasic implants are an effective and comfortable alternative solution to guided bone regeneration for patients with a large vertical bone defect, especially in selected cases.

# Abstract: Custom healing abutments as a technique to preserve the emergence profile

**Authors:** M. Costa Tort, P. Polis-Yanes, C. Cadenas Sebastián, M. Allegue Allegue, S. Egido Moreno, J. López-López

**Affiliations:** *Máster de Medicina, Cirugía e Implantología Oral. Universidad de Barcelona Campus Bellvitge*

## Introduction

 Bone and soft tissue remodeling following tooth loss is a natural physiological process. Immediate dental implants with custom healing abutments (CHAs) can stabilize emergence profiles and reduce tissue volume loss. This study aims to present two clinical cases demonstrating this technique for chair-side creation of CHAs in immediate implants.

## Material and Methods

 Two cases of lower molar implants were treated in-clinic using titanium abutments and flowable composite with this technique. This approach offers an alternative to prefabricated titanium or polyetheretherketone (PEEK) CHAs. The discussion includes other techniques for CHA creation.

## Results

 The creation of CHAs was efficient, and patients experienced favorable postoperative recoveries. Following osseointegration, peri-implant tissue appearance and bone levels were optimal for definitive crown placement.

## Conclusions

 We can conclude that this technique is an effective method for chair-side creation of CHAs using provisional titanium abutments, offering predictable outcomes at a low cost. Comparative studies are warranted to establish definitive protocols.

# Abstract: Clinical management of PTFE membrane exposure: a report of three clinical cases

**Authors:** L. Dumanova, V. Schiavo Di Flaviano, M. Domingo Mesegué, C. Arranz Obispo, J. López-López

**Affiliations:** *Máster Medicina, Cirugía e Implantología - Universitat de Barcelona – Facultad de Medicina y Ciencias de la Salud [Odontología]. C/ Feixa LLarga s/n. Hospitalet de Llobregat, Barcelona, 00907*

## Introduction

 Guided bone regeneration (GBR) is a well-established and generally predictable method for repairing alveolar ridge defects and preparing edentulous sites for implant placement. Since using nonresorbable membranes in GBR, membrane exposure has been categorized as one of the major complications associated with the procedure. Polytetrafluoroethylene membranes (PTFE) have a long history of use in GBR and different protocols for classification and management of their exposure have been published throughout these years.

## Case Report

 We present three different cases of exposure of PTFE membranes in the GBR procedure and a protocol for the management of this type of complications. In our clinical cases a single exposure of the membrane with no suppuration of about 4-6 mm in diameter was observed at the first-second week of control followed by 4-5cm exposures during next weeks. In all of these cases we applied our protocol for the management of the exposure of PTFE membranes and continued with controls until week 14-20 when the membrane was successfully removed.

## Conclusions

 After the follow-up of these patients, we propose a protocol for the management of the exposure of PTFE membranes reinforced with titanium in bone regeneration: weekly follow-up visits (biweekly maximum); control photographs; topical application of antiseptics and a formula composed of Neomycin (2.5mg/g), triamcinolone (1mg/g) and nystatin (100,000IU/g). Our goal is to avoid the need to remove the membrane and the graft and to increase the possibility of the subsequent placement of implants in the regenerated area.

# Abstract: Implanto-Supported Obturator Prosthesis as an Alternative to Palatal Defect After Oncological Surgery. Clinical Case

**Authors:** H. Slimane, C. Omaña Cepeda, A. Marí Roig, C. Moreno Soriano, E. Jané Salas, J. López-López

**Affiliations:** *Máster en odontología en pacientes oncológicos e inmunocomprometidos, Universidad de Bellvitge*

## Introduction

 Malignant neoplasms of the head and neck are the sixth most common cancer worldwide, with approximately 10,000 new cases per year in Spain alone. Treatment typically includes surgical resection of the neoplasm, as well as radiotherapy and chemotherapy, depending on the tumor type, location, extent, and stage. Due to the anatomical characteristics of the area and the need for surgical techniques with significant safety margins, patients often suffer from substantial aesthetic and functional sequelae, compromising their quality of life. The design of a rehabilitation using pre-existing implants restored aesthetics and occlusion for the patient, which are key elements in assessing the quality of life for patients with post-surgical sequelae from head and neck oncological pathology. In cases where palatal defects are extensive, dental implants are the primary alternative to achieve retention of obturator prostheses.

## Case Report

 This report presents a clinical case of a 71-year-old female patient with a history of oropharyngeal carcinoma. Treatment included hemimandibulectomy, uvulectomy, excision of the soft palate and base of the tongue, followed by 33 sessions of radiotherapy. Post-treatment, the patient could not swallow and was fed through a PEG tube, alongside experiencing significant aesthetic consequences. At the time of oncological diagnosis, she was using a hybrid prosthesis, which was removed during surgery. Prosthetic rehabilitation of the upper jaw was proposed using implants to cover the palatal defect and restore aesthetics and function. An overdenture was designed with Equator® type attachments, resulting in favorable aesthetic outcomes but limited functional improvement due to the extent of the palatal defect and the lower jaw's characteristics.

# Abstract: Prosthetic rehabilitation using digital flow in a patient with a history of oral cancer, clinical case

**Authors:** F. Alegría Riquelme, C. Omaña Cepeda, C. Moreno Soriano, P. Castañeda Vega, E. Jané Salas, J. López-López

**Affiliations:** *Máster en Odontología en paciente oncológicos en inmunocimpormetidos - Universitat de Barcelona – Facultad de Medicina y Ciencias de la Salud [Odontología]. C/ Feixa LLarga s/n. Hospitalet de Llobregat, Barcelona, 00907*

## Introduction

 Head and neck cancer patients usually present significant sequelae after their treatments, whether surgical due to the direct consequences of resective surgeries, or as a consequence of the radiotherapy and/or chemotherapy normally used: mucositis, dry mouth, trismus, among others; These "inputs" usually make conventional prosthetic rehabilitation difficult, in terms of prosthesis design and use of techniques to achieve it, making it a real challenge. Digital flow emerges as a valid alternative to rehabilitate these patients in a satisfactory and predictable way.

## Case Report

 A case of a 78-year-old male patient with a history of oral squamous cell carcinoma of the floor of the mouth treated by subtotal lingual excision with margins + bilateral cervical lymph node dissection + reconstruction with anterolateral fascio-musculocutaneous microsurgical flap is presented. left thigh, without prior or subsequent radiotherapy or chemotherapy. The lower jaw is rehabilitated using an implant-supported prosthesis (overdenture) on 6 implants and a micro-milled bar with threaded locators®, and a hybrid prosthesis with a micro-milled bar on 4 implants in the upper jaw, through the use of digital flow, obtaining satisfactory aesthetic and functional results, and in a comfortable way for the patient.

# Abstract: Autogenous Dentin and Alveolar Preservation: A Case Report

**Authors:** F. Elwy Khaled, V. Schiavo Di Flaviano, A. Torrejón Moya, E. Jané Salas, S. Egido Moreno, J. López-López

**Affiliations:** *Facultad de Medicina y Ciencias de la Salud [Odontología]. C/ Feixa LLarga s/n. Hospitalet de Llobregat, Barcelona, 00907. Máster Medicina, Cirugía e Implantología Oral, Universitat de Barcelona*

## Introduction

 Recently, extracted teeth are increasingly being used as bone grafts instead of being discarded. In treatments of alveolar bone defects and post-extraction socket preservation, various graft materials are used. Common options include autogenous grafts, allografts, xenografts, and alloplasts. Clinically, dentin is used in block or particle form, demineralized or mineralized according to its degree of demineralization.

## Case Report

 A 65-year-old woman, smoking 10 cigarettes/day and with no relevant medical history, attended the Master's program in Oral Medicine, Surgery, and Implantology at the University of Barcelona for a review and placement of a fixed prosthesis. During the intraoral examination, a significant misfit of the fixed prosthesis with inflamed areas and fistulas was observed. The panoramic radiograph showed the poor condition of the upper prosthesis, with leaks and residual root fragments, indicating the need to extract the upper residual roots 1.7, 1.6, 1.3, 1.2, 1.1, 2.1, 2.2, 2.3, 2.6, and 2.7. These roots were used as autogenous dentin, previously disinfected with a sodium hydroxide and ethanol solution for 12 minutes, and cleaned with phosphate-buffered saline for 6 minutes. It was planned to regenerate the first quadrant with beta-tricalcium phosphate and the second quadrant with autologous dentin, and to place 6 implants 6 months after alveolar preservation. In summary, in one of the alveoli, in the initial situation and in the following check-ups immediately after surgery and 6 months later, alveolar preservation with some alveolar resorption was observed in both groups, being greater in the autologous dentin group.

## Conclusions

 Autogenous mineralized dentin is a promising option as a graft material in alveolar preservation procedures due to its osteoconductive and osteoinductive properties.

# Abstract: Transcrestal sinus lift. Regarding two cases

**Authors:** C. Jorva García de Casasola, D. Sánchez Gimeno, J. Luengo Torrent, V. Schiavo di Flaviano, A. Torrejón Moya, J. López-López

**Affiliations:** *Máster de Medicina Cirugía e Implantología Oral. Facultad de Medicina y Ciencias de la Salud, UFR Odontología, Campus Bellvitge. Pavelló del Govern, Feixa Llarga s/n, 09807. Hospitalet de Llobregat, Barcelona, España*

## Introduction

 When placing osseointegrated implants in the posterior maxilla, the lack of bone availability can lead the professional to perform techniques to elevate the floor of the maxillary sinus. In orther to this, there are two main techniques, differentiating according to whether the approach is lateral to the alveolar process, or trancrestal. The transcrestal technique was described by Dr Linkow in 1960, which was based on the placement of blunt-tipped implants that allowed them to be introduced into the sinus, elevating the Schneiderian membrane with no damage.

## Case Report

 Firstly, a 60-year-old female patient with a history of carcinoma in situ in the lower lip is presented, who comes to rehabilitate tooth 2.7. The bone availability was 7mm in height and 8.80mm in width. In this case, it was decided to elevate the sinus floor. Secondly, a 53-year-old male patient with a history of hypothyroidism is presented, who were entrusted to us to rehabilitate tooth 1.6. Having 7mm in height and 8.40mm in width, it was decided to lift the sinus floor using the osteotome technique for the placement of a 10.5mm implant.

## Conclusions

 The transcrestal elevation technique has certain advantages over the lateral window approach, such as reduced morbidity and complications Even so, it is not exempted of risks and it is necessary to know its indications and limitations for occurring in the technique in a proper way.

# Abstract: Survival rate of immediate implants in periodontal patients: clinical case

**Authors:** X.X. Qin, V. Schiavo Di Flaviano, A. Torrejon Moya, E. Jané Salas, C. Arranz Obispo, L. López-López

**Affiliations:** *Facultad de Odontología. Universitat de Barcelona (UB). Campus Bellvitge, Calle de la Feixa Llarga, s/n, 08907 Hospitalet de Llobregat, Barcelona*

## Introduction

 Immediate implant placement into a fresh extraction socket has been developed as a consistent treatment, allowing for a reduction in the duration of time necessary for prosthetic rehabilitation and preventing the reduction of alveolar bone volume loss after tooth extraction. The presence of periodontal infection compromises the success of immediate implant placement. Despite this, the literature suggests that it can be successful with proper antiseptic protocols.

## Case Report

 A 60-year-old male patient comes to the clinic to continue with an unfinished treatment. The patient has a medical history of high blood pressure and hypercholesterolemia, treated with medications. He has 6 upper implants with a poorly adjusted provisional metal-resin screwed prosthesis. In the mandible with generalized advanced chronic periodontitis with type 2 and 3 mobility in remaining teeth. It is decided to extract all teeth of the lower arch and rehabilitate with 6 implants. All implants were inserted simultaneously into fresh sockets after the teeth extraction, followed by the immediate load of implants with provisional fixed dental prostheses. After 3 months of osteointegration, the patient is treated with definitive metal–acrylic resin (hybrid) in the maxillae and the mandible.

## Conclusions

 Several clinical studies demonstrated the long-term stability of the immediate functional loading of implants in periodontal patients. However, maintenance is essential for those patients to reduce biological and mechanical complications.

# Abstract: Rehabilitative treatment with dental implants in patients medicated with antiresorptives. About a case

**Authors:** J.R. Merino López, M. Costa Castillo, A. Torrejon Moya, V. Schiavo Di Flaviano, A. Estrugo Devesa, J. López-López

**Affiliations:** *Master of Oral Medicine, Surgery and Implantology (MCIO). Faculty of Medicine and Health Sciences, Dentistry. Campus Bellvitge, Calle de la Feixa Llarga , s/n, 08907 Hospitalet de Llobregat, Barcelona*

## Introduction

 Antiresorptive drugs are a class of drugs that affect bone homeostasis by inhibiting the differentiation and function of osteoclasts. The successful placement and longevity of dental implants largely depends on achieving osseointegration during wound healing, taking into account that osseointegration is a dynamic process that requires the normal functioning of the biological activities that occur during bone remodeling. , specifically the resorption of old bone by osteoclasts and the formation of new bone by osteoblasts. Therefore, medications that interfere with bone remodeling and angiogenesis may compromise osseointegration and lead to premature implant loss.

## Case Report

 76-year-old female patient with a medical history of Osteoporosis, Fibromyalgia, Typhus and Breast Cancer treated with radiotherapy, with allergy to Miolastan , polymedicated (Prolia , Morphine, Hydroferol , Calcium, Optovite B12, Ramipril , Sertraline, Simbastatin , Adiro , Emconcor , Fero gradumet, Phosphate and Omeprazole) with a surgical history of gastric bypass, coronary stent and cholecystectomy, comes to the consultation stating that he cannot eat. The clinical and radiological examination revealed limited bone availability; however, sufficient space was identified to perform an overdenture with bars in both the upper and lower jaw. Upper implants at the level of incisors and between 4 and 5, Lower implants in the intermental region.

## Conclusions

 The current literature still leaves gray areas in terms of safety of dental implant placement in patients with a history of therapy with antiresorptive or antiangiogenic drugs. General dentists and specialists must carefully select patients with due regard to medication.

# Abstract: The shocked Shield or partial extraction technique that consists of trying to preserve the Bundle Bone

**Authors:** C. Bautista Christensen, J.M. Acuña Pedrosa, M. Hilda, M. González de San Miguel, A. Boquete Castro

## Introduction

 The shocked Shield or partial extraction technique that consists of trying to preserve the Bundle Bone, a dento-dependent bone so that if a tooth is eliminated we will have yes or yes a loss of said bundle bone and, consequently, a collapse of the vestibular cortical and even of the total periphery of the alveolus. The objective of this work is to present a clinical case of socket shield technique in the jaw.

## Case Report

 57-year-old patient, controlled diabetic; no other pathologies; no allergies; non-smoker. It has a root remainder of 35 and goes to request replacement of that piece. We carried out the technique. Tugstene strawberries were used to obtain the partial extraction of the tooth. Next, a diamond strawberry was used to fine-tune the shield and its most coronal part. Subsequently, the implant bed was milled according to the indications of the commercial house and a 3.5 x 12 mm IPX model GALIMPLANT implant with a 4 mm transepithelial pillar was placed. After 4 months he has integrated we will proceed to his rehabilitation in the month of July.

## Conclusions

The socket shield technique is a predictable and adequate technique to preserve the vestibular cortical, although it is an operator-dependent technique so it is recommended that it be performed by professionals with enough surgical experience in order to avoid possible complications.

# Abstract: Guided surgery and immediate loading in maxilla implant rehabilitation

**Authors:** F. Monje Valladolid, J.A. Arazola Graña, V. Maroto Ocaña, A. Hernández Suarez, L. Monsalve Guil, E. Velasco Ortega

**Affiliations:** *Máster en Implantología Oral. Universidad de Sevilla*

## Introduction

 Currently, patients demand favorable function and high aesthetics in treatment with dental implants. The placement of immediate post-extraction implants and their immediate loading with an implant-supported restoration allows successful clinical results to be achieved. The new guided surgery protocols improve the planning of these cases in partially and totally edentulous patients. An individualized approach must be carried out, and each case must be studied since not all patients are candidates for the technique. The objective of this clinical case is to show the benefits of the technique with the latest digital advances.

## Case Report

 A 58-year-old male, he presents generalized bone loss in most of his teeth due to chronic periodontitis. The treatment plan includes the extraction of upper teeth and the insertion of implants in the same act through guided surgery and the placement of a fixed rehabilitation through immediate loading. 6 IPX implants (Galimplant®) were inserted through guided surgery. At the end of the surgery, an immediate load previously designed with the correct 3D location was placed.

## Conclusions

 The technique proves to achieve good functional and aesthetic results with the emergence profiles of the teeth, achieving a restoration with a natural appearance.

# Abstract: Anterior postextraction implants with immediate aesthetics

**Authors:** G.A. Porras Ferri, P. Garcia Gutierrez, A. González Bento, A. Hernandez Suarez, J. Moreno Muñoz, E. Nuñez Marquez

**Affiliations:** *Master of Oral Implantology. University of Seville*

## Introduction

 After tooth extraction, there is a bone remodelling process that results in a vertical and horizontal loss of the alveolar process, which causes soft tissue retraction that compromises the aesthetics of implant-supported restorations in the anterior sector. The technique of post-extraction implants with immediate aesthetics aims to minimise these hard and soft tissue changes in order to achieve a successful replacement of the lost tooth with function and aesthetics. The aim of this clinical case is to show the results obtained with the immediate post-extraction implant technique and the aesthetics achieved.

## Case Report

 A 30 year old, non-smoker patient came to the clinic and was assessed for implant treatment. The treatment plan included exodontia of the upper right central incisor due to horizontal fracture after trauma and placement of a post-extraction implant and immediate loading. Atraumatic exodontia of the fractured tooth is performed, as well as an exhaustive curettage of the alveolus. After drilling in a correct three-dimensional position, the implant (Nobel Parallel ®) is placed, measuring 3.75 mm x 11.5 mm. Subsequently, the gap is filled with heterologous bone (Apatos ®) and a connective tissue graft is placed to maintain the soft tissues and to manage the emergence profile with a screw-retained provisional prosthesis (30 Nw). Once the osseointegration time has elapsed, the definitive prosthesis is fitted.

# Abstract: Importance of soft tissues around implants

**Authors:** B.R. Ramirez Ruiz, D.M. Bellido Crespo, M. Garcia Gallego, P. Garcia Gutierrez, A. Hernández Suarez, A. Jiménez Guerra

**Affiliations:** *Máster en Implantología Oral. Universidad de Sevilla*

## Introduction

 The soft tissues around dentals implants play a crucial role in the long-term success of them, being essential for protection, aesthetics and function. It is recommended that there be 2 mm of keratinized mucosa around the implants, ideally, a thick biotype of 2-3 mm and a vertical height of 3-4 mm. The aim of this case was to show the results and importance of the soft tissues around dental implants.

## Case Report

 76-year-old female patient, totally edentulous in both arches and wearing a complete upper and lower prosthesis. Personal history: lactose intolerance; chronic cough and hepatitis. The treatment plan includes two surgical phases. In the first surgery, 5 implants (Zimmer®) are placed in positions of 45, 43, 41, 33 and 35 as well as immediate loading prostheses. Due to the existing scarcity of keratinized mucosa around these implants, in a second surgery, a free gingival graft is performed. Once the healing period has passed, rehabilitation is performed with an implant-supported hybrid prosthesis.

## Conclusions

 This clinical case shows the importance of soft tissues around implants, playing an important functional and aesthetic role.

# Abstract: Immediate implants in posterior area

**Authors:** M. Romero Barquilla, D.N. Tejada Medina, S. Haidar Wehbe, L. Monsalve Guil, J. Moreno Muñoz, E. Nuñez Marquez

**Affiliations:** *Máster de Implantología Oral. Universidad de Sevilla*

## Introduction

 Immediate implants are implants that are placed immediately after tooth extraction. In recent times, numerous methods have been investigated to increase the success of immediate implants.

## Case Report

 This clinical case presents a technique based on the placement of immediate implants using the dental root space, placing them in the palatal root for upper molars and in the distal root for lower molars, always ensuring the preservation of the septum. It is essential that tooth extraction is carried out carefully so as not to compromise the condition of the remaining bone in the alveolus, especially the vestibular table. Curettage of the alveolus is avoided in order to preserve the cells of the periodontal ligament, given its osteoinductive character. Curettage should only be performed in cases of acute infection. It is essential that the implant has contact with blood to promote bone regeneration and formation. This is a procedure with a favourable prognosis as it preserves bone height, promotes osteoformation thanks to the preservation of the periodontal ligament cells, reduces the number of surgical interventions, as well as the time until the restoration is placed, and thus reduces the migration of adjacent and antagonist teeth

# Abstract: Treatment with dental implants using the immediate loading technique

**Authors:** M. Pérez Chaves, A. González Bento, G.A. Porras Ferri, N. Matos Garrido, L. Monsalve Guil, E. Velasco Ortega

**Affiliations:** *Master of Oral Implantology. Sevilla University*

## Introduction

 Immediate loading protocols have reduced patient treatment time with a high success rate. The objective of this clinical case was to show the results of the treatment through the immediate loading of dental implants inserted with the use of biomaterials in the jaw.

## Case Report

 59-year-old partially edentulous woman evaluated for implant treatment. The treatment plan includes extractions of permanent teeth in the lower jaw, plus implant placement and bone regeneration. In the first phase, the lower extractions are performed along with bone regeneration with xenograft (Osteobiol ®), the insertion of 6 Radhex ® implants, the taking of impressions for the screw-retained lower provisional fixed prosthesis and the placement of the prosthesis within the first 24 hours. A week later, a review was performed with orthopantomography and CBCT to confirm the positive evolution of the treatment. The definitive placement of the lower rehabilitation in this case was carried out after 8 months since at the same time the upper prosthetic rehabilitation was being carried out, waiting for the osseointegration of the implants, so that the success of the treatment was ensured prosthetically. After a period of 12 months there have been no surgical or prosthodontic complications.

## Conclusions

 This clinical case suggests that prosthodontic treatment through immediate loading of dental implants is an implantological therapeutic option that has proven to be clinically successful.
